# Asian-Pacific clinical practice guidelines on the management of hepatitis B: a 2015 update

**DOI:** 10.1007/s12072-015-9675-4

**Published:** 2015-11-13

**Authors:** S. K. Sarin, M. Kumar, G. K. Lau, Z. Abbas, H. L. Y. Chan, C. J. Chen, D. S. Chen, H. L. Chen, P. J. Chen, R. N. Chien, A. K. Dokmeci, Ed Gane, J. L. Hou, W. Jafri, J. Jia, J. H. Kim, C. L. Lai, H. C. Lee, S. G. Lim, C. J. Liu, S. Locarnini, M. Al Mahtab, R. Mohamed, M. Omata, J. Park, T. Piratvisuth, B. C. Sharma, J. Sollano, F. S. Wang, L. Wei, M. F. Yuen, S. S. Zheng, J. H. Kao

**Affiliations:** Department of Hepatology, Institute of Liver and Biliary Sciences, New Delhi, India; Division of Gastroenterology and Hepatology, Humanity and Health Medical Centre, Hong Kong SAR, China; Department of Hepatogastroenterlogy, Sindh Institute of Urology and Transplantation, Karachi, Pakistan; Institute of Digestive Disease, The Chinese University of Hong Kong, Hong Kong, China; Genomics Research Center, Academia Sinica, National Taiwan University, Taipei, Taiwan; Department of Internal Medicine, National Taiwan University College of Medicine, Taipei, Taiwan; Graduate Institute of Clinical Medicine, National Taiwan University College of Medicine, Taipei, Taiwan; Department of Internal Medicine, National Taiwan University Hospital, Taipei, Taiwan; Liver Research Unit, Chang Gung Memorial Hospital and University, Chilung, Taiwan; Department of Gastroenterology, Ankara University School of Medicine, Ankara, Turkey; New Zealand Liver Transplant Unit, Auckland City Hospital, Auckland, New Zealand; Department of Infectious Diseases and Hepatology Unit, Nanfang Hospital, Guangzhou, China; Department of Medicine, Aga Khan University, Karachi, Pakistan; Beijing Friendship Hospital, Capital Medical University, Beijing, China; Seoul, Korea; Department of Medicine, University of Hong Kong, Hong Kong, China; Internal Medicine Asan Medical Center, Seoul, Korea; Division of Gastroenterology and Hepatology, National University Health System, Singapore, Singapore; Research and Molecular Development, Victorian Infectious Diseases Reference Laboratory, Melbourne, Australia; Bangabandhu Sheikh Mujib Medical University, Dhaka, Bangladesh; Department of Medicine, Faculty of Medicine, University Malaya, Kuala Lumpur, Malaysia; Yamanashi Hospitals (Central and Kita) Organization, 1-1-1 Fujimi, Kofu-shi, Yamanashi 400-8506 Japan; Department of Internal Medicine, Institute of Gastroenterology, Yonsei University College of Medicine, Seoul, Korea; NKC Institute of Gastroenterology and Hepatology, Prince of Songkla University, Songkhla, Thailand; Department of Gastroenterology, G.B. Pant Hospital, New Delhi, India; Department of Medicine, University of Santo Tomas, Manila, Philippines; The Institute of Translational Hepatology, Beijing, China; Treatment and Research Center for Infectious Diseases, Beijing 302 Hospital, Beijing, China; Peking University Hepatology Institute, Beijing, China; Division of Gastroenterology and Hepatology, Department of Medicine, University of Hong Kong, Pofulam, Hong Kong; Department of Hepatobiliary and Pancreatic Surgery, Collaborative Innovation Center for Diagnosis and Treatment of Infectious Diseases, Key Laboratory of Combined Multi-organ Transplantation, Ministry of Public Health, First Affiliated Hospital, Zhejiang University School of Medicine, Hangzhou, 310003 Zhejiang Province China; Graduate Institute of Clinical Medicine and Hepatitis Research Center, National Taiwan University College of Medicine, National Taiwan University Hospital, Taipei, Taiwan

**Keywords:** HBV, Guidelines, Acute hepatitis

## Abstract

Worldwide, some 240 million people have chronic hepatitis B virus (HBV), with the highest rates of infection in Africa and Asia. Our understanding of the natural history of HBV infection and the potential for therapy of the resultant disease is continuously improving. New data have become available since the previous APASL guidelines for management of HBV infection were published in 2012. The objective of this manuscript is to update the recommendations for the optimal management of chronic HBV infection. The 2015 guidelines were developed by a panel of Asian experts chosen by the APASL. The clinical practice guidelines are based on evidence from existing publications or, if evidence was unavailable, on the experts’ personal experience and opinion after deliberations. Manuscripts and abstracts of important meetings published through January 2015 have been evaluated. This guideline covers the full spectrum of care of patients infected with hepatitis B, including new terminology, natural history, screening, vaccination, counseling, diagnosis, assessment of the stage of liver disease, the indications, timing, choice and duration of single or combination of antiviral drugs, screening for HCC, management in special situations like childhood, pregnancy, coinfections, renal impairment and pre- and post-liver transplant, and policy guidelines. However, areas of uncertainty still exist, and clinicians, patients, and public health authorities must therefore continue to make choices on the basis of the evolving evidence. The final clinical practice guidelines and recommendations are presented here, along with the relevant background information.

## Methodology of guideline development

These APASL clinical practice guidelines represent an update of the last APASL guidelines published in 2012. The 2015 guidelines were developed by a panel of Asian experts chosen by the APASL. The clinical practice guidelines are based on evidence from existing publications or, if evidence was unavailable, on the experts’ personal experience and opinion after deliberations. Manuscripts and abstracts of important meetings published through January 2015 have been evaluated. The evidence and recommendations in these guidelines have been graded according to the Grading of Recommendations Assessment Development and Evaluation (GRADE) system (Table 1). The strength of recommendations reflects the quality of the underlying evidence, which has been classified into one of three levels, according to the GRADE system: high (A), moderate (B) or low (C). The GRADE system offers two grades of recommendation: strong (1) and weak (2) [1, 2] (Table 1). Thus, the higher the quality of evidence, the more likely a strong recommendation is warranted; the greater the variability in values and preferences, or the greater the uncertainty, the more likely a weaker recommendation is warranted. Grades are not provided for definitions.

**Table 1:** Grading of evidence and recommendations (adapted from the GRADE system) [1, 2]

	Notes	Symbol
Grading of evidence		
High quality	Meta-analysis or randomized trials without important limitations or double-upgraded observational studies^a^. Further research is very unlikely to change our confidence in the estimate of effect	A
Moderate quality	Downgraded randomized trials; upgraded observational studies^a^. Further research is likely to have an important impact on our confidence in the estimate of effect and may change the estimate	B
Low	Double-downgraded randomized trials; observational studies^a^	C
Very low quality	Triple-downgraded randomized trials; downgraded observational studies; case series/case reports^a^ Further research is very likely to have an important impact on our confidence in the estimate of effect and is likely to change the estimate. Any estimate of effect is uncertain	
Grading of recommendation		
Strong recommendation warranted	Factors influencing the strength of the recommendation included the quality of the evidence, presumed patient-important outcomes, and cost	1
Weaker recommendation	Variability in preferences and values or greater uncertainty: more likely a weak recommendation is warranted. Recommendation is made with less certainty; higher cost or resource consumption	2

^a^Cohort, cross sectional, and case–control studies are collectively referred to as observational studies. Limitations that reduce the quality of evidence of randomized controlled studies include study limitations (such as lack of allocation concealment, lack of blinding, large losses to follow-up, failure to adhere to an intention-to-treat analysis, stopping early for benefit, or failure to report outcomes), inconsistent results, indirectness of evidence, imprecision, and publication bias. Factors that can increase the quality of evidence of observational studies include large magnitude of effect, plausible confounding that would reduce a demonstrated effect, and dose–response gradient

## 1 Introduction

An estimated 240 million persons worldwide are chronically infected with hepatitis B virus (HBV) [3], placing them at increased risk of developing cirrhosis, hepatic decompensation, and hepatocellular carcinoma (HCC). Although most chronically HBV-infected subjects will not develop hepatic complications, 15–40 % will develop serious sequelae during their lifetime.

### Why this update was needed?

New data have become available since the previous APASL guidelines for management of HBV infection were published in 2012. These new data and information relate to new terminology, natural history of hepatitis B, diagnosis, assessment of the stage of liver disease using invasive and noninvasive methods, and the indications, timing, choice and duration of treatments in noncirrhotic and cirrhotic patients and in special situations like childhood, pregnancy, coinfections, renal impairment and pre- and post-liver transplant. In the current guidelines, policy recommendations for support and directions for HBV prevention and eradication in Asian countries have also been provided. The 2015 guidelines are an update to the 2012 APASL guidelines, and reflect new knowledge and evidence regarding HBV infection.

## 2 Context of guidelines

### 2.1 Epidemiology and public health burden of chronic HBV infection in Asia Pacific

HBV infection is a serious global public health problem. It is estimated that at least two billion people, or one-third of the world's population, have been infected with HBV. Approximately 240 million people, or about 6 % of the world's population, are chronically infected with HBV [3]. The prevalence of HBV infection is highly heterogeneous throughout the world, with an intermediate to high prevalence in the Asia-Pacific region, representing three-quarters of chronic HBV-positive subjects worldwide [4]. In addition, the Western Pacific region (defined by the World Health Organization as 37 countries including China, Japan, South Korea, Philippines, and Vietnam) accounts for nearly 50 % of all chronic HBV infection globally, although it has less than one-third of the world's population [5].

Prior to implementation of the HBV vaccination program, the Asian-Pacific region was divided into three categories in terms of HBsAg prevalence [6]. High-prevalence (>8 %) regions included mainland China, the Hong Kong special administrative region (SAR), Taiwan, Korea, Mongolia, Philippines, Thailand, Vietnam, and the South Pacific island nations. Intermediate-prevalence (2–8 %) regions included central Asia, the Indian subcontinent, Indonesia, Malaysia, and Singapore. Low-prevalence (<2 %) regions included Australia and New Zealand, although prevalence has increased in recent years due to immigrants from high-prevalence countries [7, 8].

Universal HBV vaccination in newborns has dramatically changed the epidemiology of chronic HBV infection. A systematic review published by WHO experts in 2012 showed a decrease in prevalence of chronic HBV infection from 1990 to 2005 in most regions of the world [3].

For example, in Taiwan, where universal vaccination of newborns was started in 1983–1985, HBsAg prevalence in children younger than 15 years of age decreased from 9.8 % in 1984 to 0.7 % in 1999, and was further reduced to 0.5 % in 2004 [9]. This has also resulted in a marked decline in the incidence of infant fulminant hepatitis, mortality associated with chronic liver disease and HCC in those born since advocacy of HBV vaccination began [10].

In mainland China, a national survey of HBV seroepidemiology has already shown a decrease in the general prevalence of HBsAg, from 9.75 % in 1992 to 7.18 % in 2006, and a decrease in children <5 years of age, from 9.67 % in 1992 to 0.96 % in 2006 [11].

In Korea, the prevalence rates of chronic HBsAg positive subjects were 4.61 % in 1998 and 2.98 % in 2010; among teenagers (10–19 years), it decreased from 2.2 % in 1998 to 0.12 % in 2010 [12].

A study conducted in Taiwan showed relative risk of HCC of 9.6 % for males who were positive for HBsAg alone, but the risk increased to 60.2 % in males who were both HBsAg- and HBeAg-positive [13]. It is estimated that approximately one-third liver cirrhosis cases and more than half of the HCC cases in the Asian region are attributable to HBV [14]. Indeed, chronic HBV infection is the dominant risk factor for HCC in most areas of Asia-Pacific. More than 700,000 new HCC cases were diagnosed in 2008, with an age-adjusted incidence of 10.8 per 100,000 worldwide [15]. Most HCC cases (>80 %) occur in eastern Asia and sub-Saharan Africa, where the incidence is >20 per 100,000 individuals [16], and is higher in males than females. For example, in Korea, the age-standardized incidence rate of HCC is 47.1 per 100,000 persons for males and 11.4 per 100,000 persons for females. In Thailand, the annual incidence is 38.6/100,000 persons for males and 17.2/100,000 persons for females, and in China it is 37.9/100,000 and 14.2/100,000 for males and females, respectively [16].

In India, where a large study in 1987 of approximately 8575 pregnant women had shown a 3.7 % incidence of HBV infection [17], a recent study of 20,104 pregnant women revealed a prevalence of around 1.1 %. The precise reasons for the decreased incidence of HBV infection could be the introduction of the HBV vaccination [18] and the wide availability of antiviral drugs to treat the primary infection in infected subjects. A large number of past studies have shown a reduction in the prevalence of HBV infection in the Indian subcontinent.

### 2.2 Terminology in chronic HBV infection

Various clinical terms relating to HBV infection have been adopted worldwide for diagnosis, staging of the disease, natural history, and treatment strategies. These can be classified into five categories:

1. Related to HBV infection
2. Related to natural history of chronic HBV infection
3. Related to response to antiviral therapy
4. Related to resistance to nucleo(s)tide analogues (NAs)
5. Occult HBV infection

#### Terminologies related to HBV infection

1. *Alanine aminotransferase (ALT) level* Determination of serum ALT level is important for starting antiviral treatment as well as for follow-up of patients with chronic HBV infection. Serum ALT level is termed as high normal serum ALT if it is between 0.5 and 1× the upper limit of laboratory reference (ULN); as low normal serum ALT if the level is ≤0.5× ULN; as minimally raised serum ALT if between ULN and 2× ULN of ALT level; and as raised ALT if >2× ULN [19]. Some authors have suggested lower values be used to define the ULN for an ALT level of 30 U/l for male and 19 U/l for female [20]. While it would be worthwhile to have the lower ALT values for early identification of liver injury and treatment of patients chronically infected with HBV, at present, the majority of countries in Asia are using ALT of 40 IU/ml as the upper limit of normal. Although there is data to suggest that patients with ALT values >0.5 times the upper limit of normal but <1.0 of ULN still have liver disease [21], there is little data to show that patients belonging to such a sub-group, if treated, respond to antiviral therapy. Due to these reasons, after due deliberations, the APASL guidelines committee suggested the use of a conventional ALT level of 40 IU/ml rather than the lowered values of 30 and 19 IU/ml for males and females, respectively (Table 2).

**Table 2:** Terminologies related to HBV infection

Terminology	Definition
ALT level	
High normal	Serum ALT between 0.5 and 1× upper limit of laboratory reference (ULN)
Low normal	Serum ALT ≤0.5× ULN
Minimally raised	Serum ALT between ULN and 2× ULN
Raised	Serum ALT 2× ULN
Chronic HBV infection	HBsAg seropositive status beyond 6 months
Low replicative chronic HBV infection	HBsAg(+), HBeAg(−) anti-HBe(+) status with persistent normal serum ALT, HBV DNA <2000 IU/ml and no evidence of liver injury
Incidentally detected HBsAg positive subject (IDAHS)	An asymptomatic individual who has been found to be HBsAg positive on routine blood screening. Such a subject could have different levels of HBV DNA and could have no evidence of liver disease to varied stages of liver disease, and hence needs to be worked up
Chronic hepatitis B	Chronic necroinflammatory disease of liver caused by persistent infection with hepatitis B virus. It can be subdivided into HBeAg-positive and HBeAg-negative chronic hepatitis B
Resolved hepatitis B infection	Previous HBV infection, but now HBsAg(−) and anti-HBs(+)
Acute exacerbation or flare of hepatitis B	Intermittent elevations of aminotransferase to more than 5 times the upper limit of normal and more than twice the baseline value
Reactivation of hepatitis B	Reappearance of active necroinflammatory disease of liver in a patient known to have the inactive chronic HBV infection state or resolved hepatitis B infection
HBeAg clearance	Loss of HBeAg in a person who was previously HBeAg positive
HBeAg seroconversion	Loss of HBeAg and detection of anti-HBe in a person who was previously HBeAg positive and anti-HBe negative
HBeAg reversion	Reappearance of HBeAg in a person who was previously HBeAg negative, anti-HBe positive
Hepatic decompensation	Defined as significant liver dysfunction as indicated by raised serum bilirubin (more than 2.5 times the upper limit of normal) and prolonged prothrombin time (prolonged by more than 3 s), or INR >1.5 or occurrence of complications such as ascites and hepatic encepahalopathy
Undetectable serum HBV DNA	Serum HBV DNA below detection limit of a PCR-based assay
2. *Chronic HBV infection* is defined as HBsAg seropositive status at 6 months or beyond.
3. *Low replicative chronic HBV infection* is defined as HBsAg(+) anti-HBe(+) with persistent normal serum ALT (PNALT) and HBV DNA <2000 IU/ml and no evidence of liver injury. This phase is also known as “inactive carrier” and “inactive chronic HBV infection.” However, the use of ‘low replicative chronic HBV infection’ term is preferred, as it explains the state of HBV infection. The term “inactive carrier” should be avoided, as HBV infection is a dynamic interaction between the host and the virus, and the inactive state could change at different time points and gives the individual an undue false sense of security.
4. *Chronic hepatitis B* is defined as chronic necroinflammatory disease of the liver caused by persistent infection with HBV. It can be subdivided into HBeAg-positive and HBeAg-negative chronic hepatitis B (CHB).
5. *Resolved hepatitis B* is defined as previous HBV infection with a current state of HBsAg(−) and anti-HBs(+)
6. *Acute exacerbation or flare of hepatitis in chronic HBV-infected patient* is defined as intermittent elevations of serum aminotransferase level to more than five times the upper limit of normal and more than twice the baseline value [22].
7. *Reactivation of hepatitis B* Reactivation of HBV replication should be defined as a marked increase in HBV replication (≥2 log increase from baseline levels or a new appearance of HBV DNA to a level of ≥100 IU/ml) in a person with previously stable or undetectable levels, or detection of HBV DNA with a level ≥20,000 IU/ml in a person with no baseline HBV DNA [22, 23]. In one earlier study, HBV DNA level of >20,000 IU/ml had a positive predictive value of 98 % in diagnosing reactivation of reactivation of HBV [23].
8. *HBeAg clearance* is defined as loss of HBeAg in a person who was previously HBeAg positive.
9. *HBeAg seroconversion* is defined as loss of HBeAg and detection of anti-HBe in a person who was previously HBeAg positive and anti-HBe negative.
10. *HBeAg reversion* is defined as reappearance of HBeAg in a person who was previously HBeAg negative and anti-HBe positive.
11. *Hepatic decompensation* is defined as significant liver dsyfunction as indicated by raised serum bilirubin (more than 2.5 times the upper limit of normal) and prolonged prothrombin time (prolonged by more than 3 s), or occurrence of complications such as ascites and hepatic encephalopathy [24].
12. *Undetectable serum HBV DNA* is defined as a serum HBV DNA level below the detection limit (<12 IU/ml) of a sensitive validated quantitative PCR-based assay.

#### Terminologies related to natural history of chronic HBV infection

Please refer to the section on natural history below.

#### Terminologies related to response to antiviral therapy

Responses can be divided into biochemical, serological, virological and histological responses. All responses can be estimated at several time points during and after therapy. The definitions of virological responses vary according to the timing (on or after therapy) and type of therapy. Two different types of drugs can be used in the treatment of CHB: immune modulators such as conventional or pegylated interferon alpha (IFN or PEG-IFN), and antiviral agents such as nucleoside/nucleotide analogues (Table 3).

**Table 3:** Terminologies related to response to antiviral therapy and resistance to NAs

Terminology	Definition
Biochemical response	Normalization of serum ALT level
Serological response	
For HBeAg	HBeAg loss and seroconversion to anti-HBe in patients with HBeAg-positive chronic HBV infection
For HBsAg	HBsAg loss and seroconversion to anti-HBs
Virological response on IFN/Peg-IFN therapy	
Virological response	HBV DNA levels below 2000 IU/ml
Sustained virological response	HBV DNA levels below 2000 IU/ml for at least 12 months after the end of therapy
Virological response on NA therapy	
Primary nonresponse	Reduction of serum HBV DNA <1 log IU/ml at 12 weeks of oral antiviral therapy in an adherent patient
Suboptimal or partial virological response	Reduction of serum HBV DNA >1 log IU/ml but still detectable at 24 weeks of oral antiviral therapy in an adherent patient
Virological response	Undetectable serum HBV DNA during therapy
Virological breakthrough	Increase of serum HBV DNA >1 log IU/ml from nadir of initial response during therapy, as confirmed 1 month later
Secondary treatment failure	Viral breakthrough in an adherent patient (due to drug resistance)
Sustained off-treatment virological response	No clinical relapse during follow-up after stopping therapy
Complete response	Sustained virological response with HBsAg seroclearance
Viral relapse	Serum HBV DNA >2000 IU/ml after stopping treatment in patients with virological response
Clinical relapse	Viral relapse along with ALT >2× ALT
Histological response	Decrease in histology activity index by at least two points and no worsening of fibrosis score compared to pre-treatment liver biopsy or fibrosis reduction by at least one point by Metavir staging
Drug resistance	
Genotypic resistance	Detection of mutations in the HBV genome that are known to confer resistance and develop during antiviral therapy
Phenotypic resistance	Decreased susceptibility (in vitro testing) to inhibition by antiviral drugs; associated with genotypic resistance
Cross resistance	Mutation selected by one antiviral agent that also confers resistance to other antiviral agents

##### Biochemical response (B)

Biochemical response is defined as normalization of ALT levels. It can be evaluated at several time points during therapy, and at the end and after the end of therapy. Since ALT activity often fluctuates over time, a minimum follow-up of at least 1 year post-treatment with ALT determinations at least every 3 months is required to confirm sustained off-treatment biochemical response. The rates of sustained off-treatment biochemical responses may sometimes be difficult to evaluate, as transient (usually <3 months duration) ALT elevations before long-term biochemical remission may occur in some CHB patients within the first year after treatment discontinuation. In such cases, additional close ALT follow-up of at least 2 years after ALT elevation seems to be reasonable in order to confirm sustained off-therapy biochemical remission [25]. However, biochemical responses may not correlate with DNA responses.

##### Serological response for HBeAg

Serological response for HBeAg applies only to patients with HBeAg-positive CHB and is defined as HBeAg loss and seroconversion to anti-HBe.

##### Serological response for HBsAg

Serological response for HBsAg applies to all CHB patients and is defined as HBsAg loss and development of anti-HBs (any titers).

#### Virological responses on IFN/PEG-IFN therapy

Responses to Peg-IFN therapy are defined differently than responses to NA therapy.

*Primary non-response* has not been well established.

*Virological response* is defined as an HBV DNA concentration of <2000 IU/ml. It is usually evaluated at 6 months and at the end of therapy, as well as at 6 and 12 months after the end of therapy.

*Sustained off-treatment virological response* is defined as HBV DNA levels below 2000 IU/ml for at least 12 months after the end of therapy.

#### Virological responses on NA therapy

*Primary non-response* is defined as <1 log 10 IU/ml decrease in HBV DNA level from baseline at 3 months of therapy.

*Suboptimal or partial virological response* is defined as a decrease in HBV DNA of more than 1 log10 IU/ml, but with HBV DNA detectable after at least 6 months of therapy in compliant patients.

*Virological response* is defined as undetectable HBV DNA by a sensitive PCR assay. It is usually evaluated every 3–6 months during therapy, depending on the severity of liver disease and the type of NA.

*Virological breakthrough* is defined as a confirmed increase in HBV DNA level of more than 1 log10 IU/ml compared to the nadir (lowest value) HBV DNA level on therapy (as confirmed 1 month later); it may precede a biochemical breakthrough, characterized by an increase in ALT levels. The main causes of virological breakthrough on NA therapy are poor adherence to therapy and/or selection of drug-resistant HBV variants (resistance).

*Sustained off-treatment virological response* NA(s) may be discontinued in some patients. Sustained off-treatment virological response may be defined as no clinical relapse during follow-up after stopping therapy.

*Viral relapse* is defined as serum HBV DNA >2000 IU/ml after stopping treatment in patients with virological response.

*Clinical relapse* is defined as viral relapse along with ALT >2× ALT.

*Complete response* is defined as sustained off-treatment virological response, together with loss of HBsAg.

*Histological response* is defined as a decrease in histology activity index by at least two points and no worsening of fibrosis score compared to pre-treatment liver biopsy, or fibrosis reduction by at least one point by Metavir staging.

*HBV resistance to NA(s)* is characterized by selection of HBV variants with amino acid substitutions that confer reduced susceptibility to the administered NA(s). Resistance may result in primary non-response or virological breakthrough on therapy.

*Genotypic resistance* is defined as detection in the HBV genome of mutations that are known to confer resistance and develop during antiviral therapy.

*Phenotypic resistance* is defined as decreased susceptibility (in vitro testing) to inhibition by antiviral drugs associated with genotypic resistance.

*Cross resistance* is defined as mutation selected for by one antiviral agent that also confers resistance to other antiviral agents.

### 2.3 Natural history of chronic HBV infection

A number of phases of chronic HBV infection are recognized, reflecting the dynamic interaction between the virus and the human host immune system. Once HBV infection has become chronic, its subsequent course largely consists of four phases of the underlying liver disease, of variable duration and outcome. All phases have been pathogenetically linked to the level of HBV replication and the strength and targets of the host immune reactivity against the replicating HBV. Transition from one phase of chronicity to the next is not recognizable in all patients, either because it may not be an obligatory step in the overall natural course of the infection, or because it is of very short duration.

#### Importance of age of acquisition of the virus

Patients who acquire HBV infection either at birth or within the first 1–2 years of life (i.e., either “vertical” or “horizontal” transmission) typically have a prolonged immune-tolerance phase, followed by an often equally prolonged immune-clearance phase. These individuals include nearly all Asian and African patients and some from the Mediterranean countries, accounting for a majority of the world’s HBV-infected population. About 70–85 % of HBeAg seroconverters remain in sustained remission, but HBeAg-negative hepatitis occurs in the remaining HBeAg seroconverters; the latter is a critically important subgroup in which progression of liver disease often continues [26]. In fact, the majority (75 %) of cirrhosis complications and HCC occur in this population of HBeAg-negative, chronic HBV-infected people [27]. An additional complexity is that HBV can cause HCC even in patients who do not develop cirrhosis.

By contrast, patients who acquire the virus after early childhood generally do not experience the immune-tolerant phase. The disease typically becomes quiescent after the immune-clearance phase, characterized by HBeAg seroconversion to anti-HBe and HBV DNA that remains at a relatively low level or becomes undetectable.

#### Phases of chronic HBV infection following vertical transmission

##### Immune-tolerant phase

In patients with perinatally acquired HBV infection, the first phase (immune tolerance) is characterized by the absence of biochemical symptoms of liver disease (i.e., elevated transaminase levels), despite evidence of active HBV replication denoted by the presence of HBeAg and HBV DNA in serum. During this phase, which may last 1–4 decades in different populations and individuals, spontaneous and treatment-induced HBeAg seroconversion is infrequent (<5 %/year). Liver biopsy during immune tolerance often reveals an absence of inflammation and scarring.

###### *Diagnosis of immune-tolerant phase*

The differential diagnosis of immune tolerance and immune clearance depends mainly on sequential determinations of serum ALT levels. However, a slightly increased serum ALT level, even though it is within the normal range, has been reported to be significantly associated with risk of liver-related mortality in the general population [28]. Therefore, some have proposed lowering the upper limit of normal (ULN) to 30 IU/l for male and 19 IU/l for female [29], although this still remains controversial. The immune tolerant phase is defined as persistence of HBeAg-positive HBV infection without significant ongoing necroinflammatory disease of the liver. Some authors have suggested that the immune-tolerant phase can be defined as having HBeAg positivity, persistently normal serum ALT levels, and serum HBV DNA >2 × 10^7^ IU/ml, with liver biopsy examination showing only minimal histological changes [30, 31]. Two important questions are: (1) What should the cutoff HBV DNA levels be for considering the patients to be in the immunotolerant phase of infection; and (2) how to predict histology without liver biopsy, based on ALT and HBV DNA levels? In two studies on HBeAg-positive patients with normal ALT and HBV DNA >2 × 10^6^ IU/ml, including 57 and 40 Asian patients, liver biopsy showed only mild disease in all, and no patient had a histological fibrosis score of >1 [32, 33]. However, in a Korean study, 28 % of HBeAg-positive patients with normal ALT and HBV DNA >2 × 10^4^ IU/ml had significant histology [34]. Also, in an Indian study of 73 HBeAg-positive patients with persistently normal ALT, 40 % had significant fibrosis. Of these patients, 23 had HBV DNA levels of ≥2 × 10^6^ IU/ml and 50 had HBV DNA levels of <2 × 10^6^ IU/ml. The median (range) of fibrosis scores among HBeAg-positive patients with persistently normal ALT was comparable between patients with HBV DNA levels ≥2 × 10^6^ IU/ml [1.0 (0.0–3.0)] and HBV DNA levels of <2 × 10^6^ IU/ml [1.0 (0.0–4.0); *p* = 0.649]. The area under ROC curve (AUROC) to determine whether there is a HBV DNA level that could differentiate patients with fibrosis from without any fibrosis on liver biopsy was 0.424, indicating that HBV DNA is a poor surrogate for fibrosis on liver biopsy [21, 35]. Thus, liver fibrosis cannot be predicted based on HBVDNA levels and ALT alone [35].

More important than defining the immune-tolerant phase is to identify patients with histological evidence of liver disease. Recent studies have found an association between even low levels of HBV DNA and CHB complications, especially in Asian patients who acquire the virus early in life [36].

The duration of the immune-tolerant phase is variable. In vertical HBV transmission from HBeAg-positive mothers, it may last for more than three decades, while under other conditions, such as in horizontal HBV spread among children, it appears to be very short and is hardly recognizable.

A study from Taiwan followed 240 patients (54 % male, mean age 27.6 years) who presented in this phase, and found that only 5 % progressed to cirrhosis and none to HCC during a follow-up period of 10.5 years [26]. These findings indicate that prognosis is generally favorable for patients who are in the immune-tolerant phase.

###### *Transition from immune tolerance to immune clearance phase*

Spontaneous HBeAg seroconversion generally occurs before 40 years of age in more than 90 % of HBsAg positive patients [37]. However, loss of immune tolerance occurs at a rate of 10–15 %/year, and patients who progress to the immune-clearance phase often face disease progression [33]. The duration of the immune tolerance phase is related to such factors as age of infection (younger > older), mode of infection (vertical > horizontal), immune status (suppressed > competent), ethnicity (Asians > non-Asians), HBV genotype C > B, D > A, baseline biochemical and histological activity (higher > lower), and ALT flare during follow-up (present > absent) [30].

##### Immune-reactive phase

During the immune-reactive phase (also known as immune active/immune clearance/HBeAg-positive CHB/HBeAg clearance phase), symptoms of liver disease may appear for the first time, as the host immune response leads to hepatocyte lysis with a flare in aminotransferase levels. Increased immune pressure on the virus during this phase is reflected by suppression of serum HBV DNA levels and accelerated clearance of HBeAg with seroconversion to anti-HBe positivity. This phase is characterized by the presence of HBeAg, high or fluctuating serum HBV DNA levels, persistent or intermittent elevation in serum aminotransferases, and active inflammation on liver biopsy. These flares may precede HBeAg seroconversion, but many flares only result in transient decreases in serum HBV DNA levels without loss of HBeAg, and some flares may lead to hepatic decompensation. More typically, the flare subsides after a variable period of time, although the associated liver injury may not regress and fibrosis can result [38]. The annual rate of spontaneous HBeAg clearance in this phase ranges from 3 to 12 %. Factors associated with higher rates of spontaneous HBeAg seroconversion include older age, higher aminotransferase levels, and HBV genotypes (A, B, D, F, B > C) [39, 40]. Genotype C is also associated with more liver injury at the time of seroconversion [41]. In a study from Alaska, it was found that after losing HBeAg, those with genotypes C and F were more likely to revert to the HBeAg-positive state as compared to those with other genotypes (A, B, D) (*p* < 0.001) [40].

This phase may end not only in HBeAg seroconversion, but also in HBsAg clearance and seroconversion to anti-HBs. However, in a number of patients, HBV replication continues despite HBeAg loss and the development of anti-HBe antibodies. The duration of this phase, and the frequency and severity of the flares, correlates with the risk of cirrhosis and HCC [42]. Recurrent flares occur more commonly in males and may explain why HBV-related cirrhosis and HCC are more common in males than in females.

HBsAg titer has been found to be higher during the immune tolerance phase than during the immune clearance phase, as well as being higher in HBeAg(+) than in HBeAg(−) patients [43, 44].

##### Low replicative phase

Although the previous phase of immune reactivity against HBV may have unfavorable outcomes, with progression of the underlying liver necroinflammation and fibrosis to cirrhosis and even to development of HCC and death, it largely terminates sooner or later in HBeAg clearance and transition to a low replicative phase. This phase is characterized by absence of HBeAg, presence of anti-HBe, persistently normal aminotransferase levels, and low or undetectable serum HBV DNA. Liver biopsy usually shows mild hepatitis and minimal fibrosis, but inactive cirrhosis may be observed in patients who had accrued severe liver injury during the preceding “immune clearance” phase. However, in 45–65 % of cases, ALT activity can fluctuate with long periods of normal ALT levels. This phase has also been referred to as the “inactive HBsAg carrier” state, but this is an erroneous label for a fair proportion of patients, given that the potential for further disease flares exists and other complications such as HCC can supervene. Indeed, for patients with infection acquired at an early age, the majority of complications occur after HBeAg seroconversion.

###### *HBV DNA levels in HBeAg-negative patients with normal ALT*

It has traditionally been believed that patients who are HBeAg negative with normal ALT have low HBV DNA levels. However, recent studies have shown that this may not always be true. Among 414 HBeAg-negative Taiwanese CHBV-infected patients with persistently normal serum ALT levels, compared to CHBV-infected patients with low–normal ALT (<0.5× ULN), those with high-normal ALT (0.5–1× ULN) had a greater frequency of serum HBV DNA levels >2000 IU/ml and a higher prevalence of core promoter mutations [45]. In another study from India, 35 % of HBeAg-negative patients with persistently normal ALT for at least 1 year had HBV DNA ≥2 × 10^6^ IU/ml. Even when the recently updated ULN values (30 IU/l for male and 19 IU/l for female) were used, 42 % of such patients had HBV DNA ≥2 × 10^6^ IU/ml [21].

###### *Histology in HBeAg-negative patients with normal ALT*

Elevated ALT has been considered to be associated with active liver disease on histology, while normal ALT has been considered to be associated with inactive histology. Many initial studies showed that among patients with chronic HBV infection with normal ALT, about 50–90 % had either minimal or mild changes (chronic persistent hepatitis) on biopsy [46–48]. Recent studies have described higher prevalence of liver injury in such patients. Among 58 Indian HBeAg-negative patients with persistently normal ALT who were biopsied, median (range) HAI was 3.0 (1.0–10.0), fibrosis score was 1.0 (0.0–3.0) and 14 % had significant fibrosis (F ≥2). In patients with persistently normal ALT as defined by updated criteria, HAI was 3.0 (1.0–81), fibrosis score was 1.0 (0.0–2.0), and distribution of fibrosis stages (0/1/2/3/4) were 35/46/19/0/0 %, respectively. Twenty-one percent of HBeAg-negative patients with persistently normal ALT (PNALT) and HBV DNA <2 × 10^4^ IU/ml had histologically active liver disease [histological activity index (HAI) ≥3 and/or fibrosis stage ≥2]. Of the 58 patients who had baseline initial liver biopsy, 28 underwent repeat liver biopsy after median 50 months (range 36–68). The median change in the Hepatic Activity Index (HAI) from initial biopsy was 2.0 (range 0–4). Six (21 %) subjects had no change in HAI, eight (29 %) had a one-point change, six (21 %) had a two-point change, six (21 %) had a three-point change, and two (7.1 %) had a four-point change. The median change in fibrosis score from initial biopsy was 1 (0–1). Eight (29 %) subjects had no change in fibrosis score and 20 (71 %) had a one-point change [21, 49, 50]. Spontaneous ALT flares occurred at 4.3 %/year among patients who were HBeAg negative with persistently normal ALT, so that cumulative probability for ALT flare was 47 % at 10 years [50]. Other studies have also found that 30–40 % of patients who exhibited normal serum ALT for more than 6 months had significant histological findings [51, 52].

###### *Long*-*term prognosis of HBeAg-negative patients with normal ALT*

Many studies have shown that although the rate of liver disease progression was associated with higher ALT levels, most cases of cirrhosis and HCC occurred in patients with ALT <45 U/l [53–55]. In another study of 3233 Chinese patients with chronic HBV infection who were grouped on the basis of ALT at presentation and followed for 4 years, it was found that the group with ALT values that were one to two times the ULN (range of comparison 0.5–6 U/l times the ULN) was at highest risk of complications of cirrhosis and HCC. However, the risk of cirrhosis and HCC was greater for the group of patients with ALT >0.5–U/l× ULN than for the group with ALT <0.5 U/l× ULN. More than two-thirds of the patients who experienced complications were already HBeAg negative when the complications occurred [27]. In a report from REVEAL study group, 1932 HBsAg-seropositive and HBeAg seronegative participants with low serum levels of HBV DNA (<2 × 10^4^ IU/ml) and 18,137 HBsAg-seronegative and anti-HCV-seronegative participants were compared. All of them had serum ALT levels <45 U/l and no HCC or cirrhosis diagnosed before or within 1 year after study entry. The multivariate-adjusted hazard ratio (95 % confidence interval) was 4.6 (2.5–8.3) for HCC incidence and 2.1 (1.1–4.1) for liver-related death for those with low replicative chronic HBV infection compared to controls [36].

##### Reactivation phase

The previous anti-HBe-positive low replicative phase is not always equivalent to a permanent termination of replication and of HBV-induced chronic liver damage. Although many patients remain in the low replicative phase for a long period of time and may also lose HBsAg (around 2 %/year), others retain or redevelop, over time, significant HBV replication and progressive liver damage [18, 19, 25]. This state of HBV-induced liver damage was first referred to as the reactivation phase, or “HBeAg-negative/anti-HBe positive chronic hepatitis B” [54]. In one study of 283 Taiwanese patients followed for a median of 8.6 years after spontaneous HBeAg seroconversion, 67 % had sustained remission, 4 % had HBeAg reversion, and 24 % had HBeAg-negative CHB. Cirrhosis developed in 8 % and HCC in 2 %, the risk being higher in those who had active hepatitis after HBeAg seroconversion [55].

It is important to differentiate patients in the low replicative phase from patients who remain at risk of progressive disease. Differentiation between these two categories of patients has been based on a HBV DNA cutoff of 2000 IU/ml [56, 57]. However, this level remains controversial. In a recent study, it has been shown that HBsAg ≥1000 IU/ml could be used to identify patients with high risk of reactivation [58]. In one Asian study, it was reported that in patients with HBV DNA <2000 IU/ml, a HBsAg level below 1000 IU/ml was associated with a 2 % incidence of HCC at 20 years, which increased to 8 % with an HBsAg level above 1000 IU/ml. This association between HBsAg and the development of HCC is not observed if HBV DNA is above 2000 IU/ml [59]. It is therefore worthwhile to reconsider whether terminologies such as inactive HBV carrier are appropriate or should be abandoned.

The reactivation phase is characterized by negative or positive HBeAg, positive anti-HBe, detectable HBV DNA, elevated aminotransferases, and continued necroinflammation. Whereas most patients reach this phase after a variable duration of low replicative state, some progress directly from HBeAg-positive chronic hepatitis to HBeAg-negative chronic hepatitis. Patients in this phase are usually older and have more advanced liver disease, as this represents a later phase in the course of chronic HBV infection. Serum HBV DNA levels are lower than in HBeAg-positive patients, but may be high. The high levels of serum HBV DNA result from a spontaneous mutation in the core or core promoter region of the viral genome [60]. The precore mutation produces a stop codon in a region of the HBV genome that prevents the formation of HBeAg, whereas the basal core promoter (BCP) mutation affects HBeAg transcription. These mutations, either singly or in combination, permit HBV replication in the absence of HBeAg. The hallmark of this phase is its fluctuating course. In a study of 164 anti-HBe-positive patients who were monitored at monthly intervals for a median period of 21 months, 64 % had fluctuating ALT levels, including 44 % whose ALT levels were intermittently normal [61]. Several investigators have attempted to define cutoff HBV DNA levels that would differentiate patients with HBeAg-negative chronic hepatitis from inactive carriers, but in view of the fluctuating course, serial testing is more reliable than a single test [62].

A recent study found that reactivation of hepatitis B following HBeAg seroconversion correlated significantly with genotype C (*p* = 0.003), male sex (*p* = 0.03), ALT levels >5× upper normal limit during the HBeAg-positive phase (*p* = 0.02), and age at HBeAg seroconversion ≥40 years (*p* = 0.002) [63].

HBeAg-negative CHB was originally reported in Mediterranean countries, but has now been reported in all parts of the world. Currently, HBeAg-negative CHB represents the most common type of CHB, particularly in European, African and Middle East countries of the Mediterranean Basin.

Spontaneous HBsAg seroclearance has been reported to occur at a rate of 0.5–1 %/year in patients with chronic HBV infection [64]. HBsAg seroclearance is generally accompanied by undetectable serum HBV DNA, normalization of liver biochemistries, and improved liver histology [65]. However, HCC has been reported in a small percent of patients, the risk being higher in those with cirrhosis, HCV coinfection, or older age at the time of HBsAg seroclearance [66].

HBsAg levels are important in predicting HBsAg loss during follow-up. One Asian study found that in HBeAg(−) patients with persistently normal ALT, a decline ≥1 log10 IU/ml during a 2-year time period or a single measurement below 200 IU/ml are the best predictors of HBsAg loss [positive predictive value (PPV) 100 %] [66]. Also, a threshold of HBsAg decline ≥0.3 log10 IU/ml/year identifies patients with high probability of HBsAg loss with a negative predictive value (NPV) of 95 % and a PPV of 85 % [58].

#### Phases of chronic HBV infection following horizontal transmission

Horizontally acquired disease also evolves through a number of phases with active replication and hepatic necroinflammatory activity in the early months and years of chronic HBV infection. With time, replication often diminishes and host immune pressure results in HBeAg/anti-HBe seroconversion. This is followed by a quiescent phase of infection with lessened liver injury and evolution into an inactive HBV infection state. Certain patients appear to suffer little morbidity after HBeAg seroconversion. For instance, studies of HBsAg-positive Italian patients in the inactive infection state, who were initially identified when they were rejected as blood donors, showed that these individuals experienced no appreciable increase in liver-related morbidity over many years [64]. This observation reflects the benefit of HBeAg seroconversion following adult acquisition of HBV; that is, this event typically leads to a durable decrease in viral activity and liver damage.

#### Predictors of disease progression in chronic HBV infection

##### Chronic HBV infection and cirrhosis

The annual incidence of cirrhosis has been estimated to be 2–6 % for HBeAg-positive and 8–10 % for HBeAg-negative patients. The higher rate of cirrhosis among HBeAg-negative patients is related to older age and more advanced liver disease at presentation. Among HBeAg-positive patients, the rate of cirrhosis development is higher in those who remained HBeAg positive during follow-up. Additional factors have been identified to be associated with progression to cirrhosis: habitual alcohol intake, concurrent infection with hepatitis C virus (HCV) or human immunodeficiency virus (HIV), high levels of HBV replication, and patients who had HBeAg reversion, HBV genotype (C > B) [67, 68] and a higher proportion (>45 %) of BCP mutataion [69]. In one study of 3774 HBsAg chronic HBV-infected subjects aged 30–65 years, the adjusted relative risk of cirrhosis for patients with baseline serum HBV DNA >10^4^ and >10^6^ copies/ml was 2.3 (95 % CI 1.6–3.5) and 9.3 (95 % CI 6.5–13.1), respectively [70]. Collectively, these data suggest that persistent high levels of HBV replication (with accompanying hepatitis) increase the risk of cirrhosis, but the prognostic significance of a high serum HBV DNA level at a single time point in a young HBV-infected subject (<30 years old) is unclear.

##### Chronic HBV infection and HCC

The annual incidence of HCC has been estimated to be <1 % for noncirrhotic chronic HBV-infected patients and 2–3 % for patients with cirrhosis. Additional risk factors for HCC include coinfection with HCV, a family history of HCC [71], habitual alcohol intake, high levels of HBV replication HBV genotype C > B) [72], and core promoter mutations [73], as well as obesity, diabetes, and smoking [74].

### 2.4 Clinical significance of HBV genotypes and common mutants

Based on the extent of divergence in the entire HBV genomic sequence, at least ten HBV genotypes (A–J) and several subtypes have been identified: >8 % for genotypes and 4–8 % for subtypes. Genotype A is highly prevalent in sub-Saharan Africa, Northern Europe, India and Western Africa. Genotypes B and C are common in Asia. Genotype C mainly exists in East and Southeast Asia. Genotype D is prevalent in Africa, Europe, the Mediterranean region and India. Genotype E is restricted to West Africa. Genotype F is found in Central and South America. Genotype G has been reported in France, Germany, and the United States. Genotype H is found in Central America [75]. Geographic distribution of HBV genotype may correlate with the modes of transmission. For example, genotypes B and C are prevalent in highly endemic areas where perinatal or vertical transmission plays an important role in the viral spreading, whereas the remaining genotypes are frequently found in areas where horizontal transmission is the main mode of transmission.

In a study from Japan, the persistence of HBV infection after acute hepatitis B was higher in patients with genotype A (23 %) than in those with genotype B (11 %) or C (7 %) infection [76]. The rate of chronicity after acute genotype D infection has also been reported to be relatively high [77].

HBV genotype C patients may experience delayed HBeAg seroconversion and a lengthier period of active HBV replication than genotype B patients. With these unfavorable features, genotype C patients are more prone to develop advanced fibrosis, cirrhosis, and even HCC than genotype B patients [78–80].

Compared with genotypes C and D patients, genotype A and B patients had a higher rate of spontaneous HBsAg seroclearance [81, 82].

Genotype C infections conferred a higher frequency of BCP A1762T/G1764A mutation than genotype B, and HBV viral load was higher in genotype C than in genotype B patients [72]. Similarly, genotype D-infected patients who had more progressive liver disease had a higher prevalence of BCP A1762T/G1764A mutation than those with genotype A infection [83]. Frequency of pre-S deletion was significantly higher in genotype C patients than in genotype B patients, and pre-S deletion is associated with higher risk for HCC development [84].

HBV genotype A has better responses to IFN-a treatment than genotype D patients, regardless of HBeAg status. Further, HBV genotype B has a higher response rate to IFN-a treatment than genotype C in HBeAg-positive patients [85].

There is no significant association between HBV genotype and response to nucleos(t)ide analogues [85].

## 3 Guidelines

### 3.1 Screening for chronic HBV infection

The impact of vaccination has been profound in reducing the global burden of HBV, particularly in children and young adults, but millions of chronic HBV-infected patients remain. Seroprevalence studies have been widely performed and show that chronic HBV infection continues to be a major health problem; a representative case was that of China, where the seroprevalence rate in 1992 was 9.8 % and was reduced to 7.2 % in 2006 after vaccination. While these optimistic trends do indicate an eventual eradication of the virus, this would appear to be many decades away. In the interim, there is good established treatment for patients chronically infected with HBV that can reduce liver-related outcomes [86], although HBsAg clearance is still not a realistic goal. With the World Health Organization (WHO) resolution on viral hepatitis, the WHO has launched a number of initiatives [87], which include the Global Hepatitis Network and a Framework for Action, in order to tackle these issues. It is recognized that one of the major obstacles to action remains the large burden of undiagnosed cases of chronic HBV infection around the globe. However, estimates of such a hidden burden of disease are poorly documented. In a large cross sectional study screening for hepatitis B amongst Asian Americans in San Francisco (*n* = 3163), 65 % of those who tested HBsAg positive were unaware they had had chronic HBV infection—either they had never been tested before or had not been previously diagnosed [88]. In a US-based insurance cohort study, the difference in the proportion of patients who tested positive for HBsAg compared to the expected number estimated from the NHANES study was 21 % [89]. A study from Italy showed that based on HBV prevalence data of 1.29 % from the Ligurian region, there should be 20,438 chronically infected patients, but only 445 (2.2 % of the estimated chronic HBV infection population) were actually chronically infected on follow-up [90]. European estimates indicate that three-quarters of those infected with chronic HBV infection are unaware of their infection [91]. In Asia, a Japanese study on HBV and HCV prevalence examined patients, such as first time blood donors and those having a periodic health examination, who were unaware of their hepatitis status. The prevalence of HBV in this population was estimated to be 0.63 % or 68,792 persons [92]. In general, there are few studies that examine this issue of under-diagnosis of chronic HBV infection, and approaches that can resolve the issue. It is estimated that 45 % of people living with CHB remain undiagnosed, resulting in poor health outcomes and risk of transmission [93].

#### Principles of screening

In a key article published over 40 years ago, the World Health Organization established several principles for health screening [94]. In this article, the criteria were:

1. Screening should be directed towards an important health problem
2. There should be a simple, safe, precise and validated screening test
3. Treatment started at an early stage should be of more benefit than treatment initiated later
4. There should be evidence that the screening test is effective in reducing mortality and morbidity
5. The benefit of screening should outweigh the physical and psychological harm caused by the test, diagnostic procedures and treatment
6. The opportunity cost of the screening program should be economically balanced in relation to expenditure on medical care as a whole
7. There should be a plan for managing and monitoring the screening program and an agreed set of quality assurance standards
8. Potential screening participants should receive adequate information about benefits and disadvantages of participation
9. Case finding should be a continuing process and not a once-and-for-all project

Chronic HBV infection clearly falls into this category; consequently, screening to detect those with CHB infection is a justifiable exercise.

#### Screening and linkage to care

A large number of studies of epidemiology of chronic HBV infection only examine those who are detected to be HBsAg seropositive, but little is known of screening uptake (% of patients who agree to take the test), and of these, how many were referred and evaluated as requiring therapy. Consequently, screening to detect seropositive patients is insufficient as a management strategy, without proper linkage to care. The Institute of Medicine recommendations [95], while specific to the US, can be broadly applied to many other countries as well. They found that the US infrastructure for management of chronic viral hepatitis was poor, and broadly recommended three important initiatives: increased disease surveillance, improved provider and community education, and integration and enhancement of viral hepatitis services. In particular, the viral hepatitis services should encompass five core elements in a coordinated and comprehensive manner—outreach and awareness; prevention of new infections; identification of infected people; social and peer support; and medical management of infected people, as otherwise newly diagnosed patients will be lost and will not receive the benefit of potential therapy that may be lifesaving.

Consequently, the logistic chain of screening begins with information and education, followed by agreement to undergo testing, testing itself, and then evaluation; it ends with treatment in those who need it. A good example of the approach to screening and linkage to care is the Hepatitis Outreach Network, which combines the expertise and resources of the Mount Sinai School of Medicine, the NYC Department of Health and Mental Hygiene, and community-based organizations [96]. A similar study was undertaken in Sheffield in the UK [97]. Consequently, many stakeholders need to come together and coordinate efforts and resources in order for this strategy to be effective. However, screening itself is a major exercise.

#### Evidence for screening

While it seems sensible and rational to perform screening for chronic HBV infection, a screening strategy needs to have evidence of efficacy, based on evidence that screening reduces mortality or complications of disease. Some screening strategies are potentially harmful, particularly in the case of cancer screening, when there are false negative or positive results, adverse events of labeling or early diagnosis and adverse effects of treatment or investigation [98]. Consequently, proof of efficacy relies on randomized control trials of screening using one of two designs [98]—the first is randomized to screening versus no screening, with treatment of those screened and found to be suitable for therapy; the second is where all participate in screening and those with positive test results are randomized to treatment or no treatment. In both scenarios, a significant difference in outcome (e.g., liver cancer, cirrhosis or mortality) then favors the screening arm. Unfortunately, no such studies have been performed in chronic HBV infection, and it would seem that such studies are unlikely, since the lead time to development of such complications would take many decades. Secondly, the second screening strategy of not treating if there is a positive result may be ethically difficult to carry out, if patients fulfill treatment criteria. Consequently, evidence for screening is largely based on observational data. In the REVEAL study [53], 164 cases of HCC were detected during follow-up. In evaluation of cirrhosis, during the initial screening, 436 cases of cirrhosis were found, and a further 365 cases were discovered during follow-up [99]. There was also a significant increase in liver-related mortality [100]. Most screening studies did not examine clinical outcomes, but rather, the number of patients screened and the number of positive HBsAg cases found. As the largest and most comprehensive screening program, the BFreeNYC program reached 11,000, screened approximately 9000 people, and diagnosed and managed six cases of HCC and 22 of end-stage liver failure [101]. These studies show that screening does pick up significant cases of advanced liver disease and their complications. While screening may potentially detect such complications, whether screening followed by treatment would prevent such complications has not yet been demonstrated. Treatment for chronic HBV infection has reduced outcomes in patients with significant liver fibrosis or advanced liver disease, and treatment of chronic HBV infection for those without cirrhosis has shown to improve surrogate markers such as LFTs, liver histology and HBeAg seroconversion [102]. While cancer screening programs can have potentially harmful consequences due to nonspecificity of tests (leading to anxiety and unnecessary testing), this does not appear to be the case with screening for hepatitis B. In addition, in the screening test for hepatitis B, HBsAg has a high level of sensitivity and specificity [103], making false positives or negatives extremely low. However, social issues, including discrimination and stigmatization of the patients, need to be addressed adequately before embarking on screening programs.

#### Types of screening

There are several types of screening: mass screening or population screening involves screening a large population, multiphasic health screening involves a battery of screening tests on the same occasion, and opportunistic screening refers to screening offered to patients who attend a health practitioner for some other reason.

Population-based screening is where a test is offered systematically to all individuals in the defined target group within a framework of agreed policy, protocols, quality management, monitoring and evaluation. This involves considerable infrastructure and protocols. Such a scheme does not appear to have been established for chronic HBV infection in most countries. Establishment of a screening strategy then requires deliberation on the mode in which the strategy is delivered. Such interventions have to be tested in randomized control trials to determine which have the best outcomes in terms of proportion of patients taking up screening, proportion of patients that test positive and proportion who require treatment.

Opportunistic screening is less organized and generally less effective, as it relies on the healthcare worker to remember to initiate the process, to provide information and education, and to inform about the testing process and consequences if tested positive, and options for therapy, all of which involve considerable time and effort. In an excellent systematic review of community screening strategies for chronic HBV infection, Robotin and George [104], reviewed strategies that specifically excluded screening conducted by state and local public health departments. They categorized programs into four models:

(A) Community clinic model with screening integrated into routine primary care services. Screening is based on risk factors and doctors provide counseling and testing referrals
(B) Community outreach model, which involves screening in community settings (e.g., health fairs) and volunteers providing logistic support
(C) Partnership and contract model, where screening is outsourced to a general health screening company
(D) Outreach and partnership model, which contains elements of (B) and (C), where screening occurs in community setting with a community organization that has direct links to the target community

The systematic review found that screening uptake was highest for programs using an outreach and partnership model (C), while the community outreach model (B) had less uptake, and screenings offered by clinical experts had low uptakes (1–2 %). Successful linkage to care was offered by some programs, but many programs had high dropout rates. No data on the proportion of patients requiring treatment or referral for treatment was provided. The overall evaluation was that these screening programs had at best screened modest numbers of patients, considering the global burden of disease. The authors felt that the most successful programs achieved significant buy-in from target communities, delivering culturally appropriate educational initiatives and offering comprehensive care packages, not just screening alone.

Whichever screening strategy is employed, the logistics of implementation need to be established. A key aspect of this is the consent and information to be provided to the patient. Counseling is crucial to educate and inform patients about chronic HBV infection, the consequences and sequelae of chronic infection and the treatment options available. Also, advice on what is to be done if the test is positive and the linkage to care need to be established. Aids such as flyers, leaflets, websites, trained counselors and trusted community contacts can be used to help patients understand this better. Proper clinical studies are needed to test whether such methods are useful in increasing screening uptake.

#### Risk factor screening

Certain groups are at higher risk of acquisition of HBV and of becoming chronically infected. There is a need for targeted screening for HBV infection in high-risk individuals because the infection remains asymptomatic in a vast majority of infected individuals, especially those who acquire infection at birth or during childhood. Moreover, chronic infection leads to the development of cirrhosis, liver failure, or HCC. Identification of a HBV-infected person is helpful to [7, 105]:

- detect and evaluate stage of the liver disease and extent of liver damage;
- plan antiviral therapy which can delay or reverse the progression of liver disease;
- permit ultrasound surveillance to detect HCC at a potentially treatable stage;
- counsel to avoid excessive alcohol use;
- take measures to reduce risk of transmission to others;
- avoid unnecessary vaccination, as vaccination is *not* beneficial for persons already chronically infected and is unnecessary for persons already immune (either through prior vaccination or a previous resolved acute infection;
- vaccinate unprotected individuals.

The prevalence of HBV varies markedly between different countries of the Asia Pacific region. The prevalence of chronic infection ranges from 10 % of the population in China to <2 % in Australia [6]. So there are areas of high, medium, and low endemicity based on a prevalence of HBsAg positivity of ≥8, 2–7, and <2 %, respectively [106].

In countries with high endemicity, >90 % of new infections occurred among infants and young children as the result of perinatal or household transmission, while in countries of low endemicity (i.e., HBsAg prevalence of <2 %), the majority of new infections occur among adolescents and adults as a result of sexual and injection-drug use exposures. In countries of intermediate HBV endemicity, multiple modes of transmission operate, i.e., perinatal, household, sexual, injection-drug use, and health-care related.

Screening of the general population may be cost effective in finding new cases in countries with high prevalence, but it is not in regions with low prevalence. In countries with intermediate prevalence, it would depend upon the socioeconomic status. However, it is worth doing screening of ‘high-risk groups’ irrespective of prevalence and socioeconomic status.

The following groups should be tested for HBV infection [7, 107–110]:

- Persons with liver disease
- Persons needing immunosuppressive or cancer chemotherapy
- Injection drug users (IDU)
- Persons who have received unsafe injections (used syringes or needles)
- Men who have sex with men (MSM)
- Persons with multiple sexual partners or history of sexually transmitted infection
- Family members, household contacts and sex partner of a person with hepatitis B
- Inmates of correctional facilities
- Dialysis patients
- HCV- or HIV-infected individuals
- Pregnant female (preferably during the first trimester to vaccinate unprotected mothers)
- Infants born to females with chronic HBV
- Blood or organ donors
- Health care workers

#### Screening in special populations

Antenatal screening for hepatitis B in pregnant females to identify newborns who require prophylaxis against perinatal infection is a well-established, evidence-based standard of practice [111]. This has become even more important, as new strategies to even further reduce perinatal transmission using nucleos(t)ide analogues in the last trimester of pregnancy haves been established through randomized control trials [112, 113]. However, the effectiveness of such screening programs in real life is not ideal. In a large prospective study [114], the impact of the GAVI project on reducing perinatal HBV infection was evaluated. This included a proportion of pregnant females screened for HBV. Between 2002 and 2009, using a cluster sampling methodology in Eastern, Central and Western regions of China, 244 facilities were assessed with 71,694 live births in 2002 and 125,874 live births in 2009. The HBV screening rate increased from 64 % in 2002 to 85 % in 2009. Consequently, there is still room for improvement. With regard to blood safety, this is clearly an important area to ensure high compliance. A recent report in Morbidity and Mortality Weekly Report (MMWR) [115] indicates that the number of countries in Africa and sub-Saharan Africa testing at least 95 % of donations for HBV increased from 76 to 94 %. Nucleic acid testing (NAT) is not widely available in the developing world [116], and is now considered a standard of care in blood safety. In Asia, there are few audits of blood safety measures in developing countries; consequently, it is unclear to what extent is blood safety is established.

##### Tests used for screening

Screening tests are inexpensive and cost effective in populations at higher prevalence, as cost per case identified decreases, and they have the potential to reduce HBV-associated morbidity and mortality [117]. The individuals found to be negative during the screening should be vaccinated, and cases identified should be counseled and treated.

The HBsAg test is the primary way to definitively diagnose chronic HBV infection. The anti-HBs test will tell if your patient is protected against HBV. Anti-HBs antibody can be produced in response to vaccination, recovery from an acute hepatitis B infection, or the presence of less common pre-S mutants [118].

The total hepatitis B core antibody (total anti-HBc) test tells if a person has been previously exposed to HBV [119]. The test by itself does not indicate whether immunity or chronic infection has developed as a result of exposure. This test can be utilized for screening, but anti-HBc positive individuals should be further tested for both HBsAg and anti-HBs to differentiate infection from immunity. However, both HBsAg and ant-HBs may be negative. In such a case, patients with immunity show anamnestic response after one dose of HBV vaccine, while patients with occult infection do not [120]. This test may be false-positive in low prevalence areas. Patients with false-positive results will need a full course of vaccine to have an immune response. Anti-HBc antibody is also positive during the window phase of acute hepatitis B, i.e., after the disappearance of HBsAg and before the anti-HBs develop. Individuals with past HBV infection (anti-HBc reactive) should not donate blood even if they have recovered.

3.1 Recommendations (screening for chronic HBV infection).
  3.1.1 Screening for hepatitis B infection is an important tool to discover new cases of chronic infection (A1).
  3.1.2 There is insufficient evidence to recommend any specific screening strategy for CHB and further research is needed in this crucial area (C1).
  3.1.3 Existing screening strategies in antenatal care and blood supply should be strengthened (A1).
  3.1.4 Screening in high-risk populations should continue to be a high priority (A1).
  3.1.5 Strategies to enhance screening acceptance and uptake should be undertaken (C1).
  3.1.6 High-risk persons who are most likely to be infected with HBV and should be tested for chronic HBV infection include (B1):
    Persons with liver disease
    Family members, household contacts, infants, sex partners of a person infected with hepatitis B
    Persons needing immunosuppressive or cancer chemotherapy
    Injection drug users (IDU)
    Persons who receive unsafe injections (used syringes or needles)
    Persons who have sex with males (MSM), with multiple sexual partners, STDs
    Inmates of correctional facilities
    Dialysis patients
    HCV- or HIV-infected individuals
    Pregnant females (preferably during the first trimester, to vaccinate unprotected mothers)
    Health care workers
    Blood or organ donors
  3.1.7 Testing should include a serological assay for HBsAg (A1), anti-HBs (B2) and total anti-HBc (B2).
  3.1.8 Screening should be linked to appropriate counseling and referral for further care including clinical evaluation, need for treatment and vaccination (if found to be negative for HBV infection) (C1).

### 3.2 Counseling and prevention of transmission of hepatitis B from individuals with chronic HBV infection

Patients with chronic HBV infection should be counseled regarding lifestyle modifications and prevention of transmission, as well as the importance of lifelong monitoring. No specific dietary measures have been shown to have any effect on the progression of CHB. However, heavy use of alcohol (>20 g/day in female and >30 g/day in male) may be a risk factor for the development of cirrhosis [121].

Persons chronically infected with HBV should be counseled regarding transmission to others (Table 4). Household members and steady sexual partners are at increased risk of HBV infection and therefore should be vaccinated if they test negative for HBV serological markers. For sex partners who have not been tested or have not completed the full immunization series, barrier protection methods should be employed.

**Table 4:** Recommendations for infected persons regarding prevention of transmission of HBV to others

Have sexual contacts vaccinated
Use barrier protection during sexual intercourse if partner not vaccinated or naturally immune
Do not share toothbrushes or razors
Cover open cuts and scratches
Clean blood spills with detergent or bleach
Do not donate blood, organs or sperm
Can participate in all activities including contact sports
Children should not be excluded from daycare or school participation and should not be isolated from other children
Can share food, utensils, or kiss others

The risk of infection after blood transfusion and transplantation of nonhepatic solid organs (kidneys, lungs, heart) from persons with isolated anti-HBc is low: 0–13 % [122]. The risk of infection after transplantation of liver from HBsAg-negative, anti-HBc-positive donors has been reported to be as high as 75 % and is related to the HBV immune status of the recipients [123]. If anti-HBc-positive donor organs are used for HBV seronegative recipients, antiviral therapy should be administered to prevent de novo HBV infection. While the optimal duration of prophylactic therapy has not been determined, a limited duration, such as 6–12 months, may be sufficient for transplantation of non-hepatic solid organs. For transplantation of livers, life-long antiviral therapy is recommended, but whether HBIG is necessary is unclear [124].

HBsAg-positive female who are pregnant should be counseled to make sure they inform their providers so that appropriate decisions regarding administering hepatitis B immune globulin (HBIG) and hepatitis B vaccine can be made for their newborn immediately after delivery. HBIG and concurrent hepatitis B vaccine have been shown to be 95 % efficacious in the prevention of perinatal transmission of HBV; the efficacy is lower for mothers with very high serum HBV DNA levels (>7–8 log10 IU/ml) [125, 126]. In a recent analysis comparing the cost-effectiveness of HBV control strategies combining universal vaccination with hepatitis B immunoglobulin (HBIG) treatment for neonates of chronically HBV-infected mothers, it was concluded that HBIG treatment for neonates of HBsAg positive mothers is likely to be a cost-effective addition to universal vaccination, particularly in settings with adequate health care infrastructure. Targeting HBIG to neonates of higher risk, HBeAg-positive mothers may be preferred where willingness to pay is moderate. However, in very resource-limited settings, universal vaccination alone is optimal [127].

Transmission of HBV from infected health care workers to patients may occur in rare instances (see “3.13.4 Health care workers” section).

3.2 Recommendations: counseling and prevention of transmission of hepatitis B from individuals with chronic HBV infection:
  3.2.1 Chronic HBV-infected persons should be counseled regarding prevention of transmission of HBV (Table 4) (A1).
  3.2.2 Sexual and household contacts of chronic HBV-infected persons who are negative for HBV seromarkers should receive hepatitis B vaccination (A1).
  3.2.3 Abstinence of alcohol is recommended in chronic HBV-infected subjects (A1).
  3.2.4 Chronic HBV-infected subjects should not be discriminated and stigmatized in the society or in their work place (A1).
  3.2.5 HBV-infected children should not be isolated in the educational and social environment (A1).

### 3.3 Assessment of persons with chronic HBV infection

The initial evaluation of an individual with HBV infection should include a detailed history and physical examination. Alcohol consumption, family history of HBV and HCC, and assessment of risk factors to determine the likely mode of HBV acquisition and possible superinfection with other hepatitis virus(es) should be part of the history taking. Comorbidities such as obesity, diabetes mellitus and metabolic syndrome should be assessed. Hepatic steatosis in individuals with CHB is related to co-existent metabolic factors rather than being virally induced [128, 129]. The physical examination focuses on identifying presence of cirrhosis or decompensated liver disease, as it has an impact on prognosis. A complete blood count, biochemical tests, serological and virological markers of HBV, and hepatic ultrasound should be part of the initial evaluation. The biochemical tests include ALT, AST, GGT, alkaline phosphatase, serum albumin and prothrombin time. The virological assessment consists of HBeAg, anti-HBe antibodies and Hepatitis B DNA measurement, the latter being the best marker of viral replication [130]. A real-time PCR quantification assay should be used to measure serum HBV DNA levels [131, 132].

Other causes of chronic liver disease should be systematically looked for, including coinfections with HDV, HCV and/or HIV. Comorbidities, including alcoholic, autoimmune, and metabolic liver disease with steatosis or steatohepatitis should be assessed.

In addition, all first-degree relatives and sexual partners of patients with chronic HBV infection should be advised to get tested for HBV serological markers (HBsAg, anti-HBc, anti-HBs) and to be vaccinated, if they are negative for these markers.

In subjects with chronic HBV infection, accurate assessment of the extent of hepatic fibrosis and/or the severity of necroinflammatory activity is essential for choosing therapeutic strategies and for monitoring the responses to anti-viral or anti-fibrotic treatment. Knowledge of the underlying histology can help guide therapeutic decisions when patients do not meet the clinical practice guidelines and treatment may be helpful. Aminotransferase levels may fluctuate with time, and single measurements of ALT and AST do not indicate disease stage. Usually, the ALT concentrations are higher than those of AST, but with disease progression to cirrhosis, the AST/ALT ratio may be reversed. A progressive decline in serum albumin concentrations, rise in bilirubin and prolongation of the prothrombin time are characteristically observed as decompensated cirrhosis develops. In chronic HBV infection, a liver biopsy is usually recommended to determine the stage of fibrosis and/or the grade of activity in patients with a high viral load and high-normal or minimally raised ALT levels and in those older than 30 years without clinical evidence of cirrhosis. Liver biopsy is considered the reference standard for the histological evaluation of liver disease. However, it is important to remember that a liver biopsy represents just ~1/50,000 of the entire liver, and that liver injury is typically irregularly distributed in the liver. Thus, liver biopsy is an imperfect reference standard; taking into account a range of accuracies of the biopsy, even in the best possible scenario, an area under the receiver operating characteristic (AUROC) >0.90 cannot be achieved even for a perfect marker of liver disease [133]. The diagnostic accuracy of liver biopsy decreases because it is often subject not only to sampling error, but also to intra- and inter-observer variability in histological interpretation [134]. Moreover, even if it is generally accepted to be a safe procedure, it is invasive and can be associated with rare but potentially serious complications, including hemorrhage, pneumothorax, and procedure-related mortality. Thus, although there is still an important role for liver biopsy among chronic HBV infection, there is an obvious need to develop and use noninvasive, accurate, and reproducible tests for detecting liver injury. For example, noninvasive tests are helpful in assessing the stage of fibrosis in chronic HBV infection with no clear indication for a liver biopsy, or in those who require follow-up assessment of the stage of fibrosis during or after treatment.

Several noninvasive tests based on serum fibrosis markers or radiographic techniques have been introduced, and they are being increasingly used to assess the severity of liver disease in clinical practice. These include serum biochemical parameters, such as the ratio of aspartate aminotransferase (AST) to ALT, the fibrosis score-4 (FIB-4), the AST to platelet ratio index (APRI), the age-spleen-platelet index, the Forns index, and the Hui index. Specialized tests include Fibrotest, Hepascore, the enhanced liver fibrosis test and, for elasticity imaging, magnetic resonance (MR) elastography and transient elastography (TE) [135, 136].

The APRI is a simple test that is readily available, is inexpensive, does not require particular expertise in interpretation, and can be performed in an outpatient setting. APRI uses two cutoff points for diagnosing specific fibrosis stages, as the use of a single cutoff would result in suboptimal sensitivity and specificity. A high cutoff with high specificity is used to diagnose persons with a particular stage of fibrosis, and a low cutoff with high sensitivity (i.e., fewer false-negative results) is used to rule out the presence of a particular stage of fibrosis. Some persons will fall in the indeterminate range of test results (i.e., their score will be between the low and the high cutoff) and will need future re-testing and evaluation. Most commonly reported cutoff values for APRI for the detection of significant fibrosis and cirrhosis are as follows: For significant fibrosis (METAVIR ≥F2), low and high cutoffs for APRI are 0.5 and 1.5; and for cirrhosis (METAVIR F4), low and high cutoffs for APRI are 1.0 and 2.0. Sensitivity, specificity, PPV and NPV for diagnosing significant fibrosis (METAVIR ≥F2) were 71–84, 50–69, 52–61 and 76–84 % for APRI low cutoff; and 28–45, 90–95, 68–81 and 65–72 % for APRI high cutoff. Sensitivity, specificity, PPV and NPV for diagnosing cirrhosis (METAVIR F4) were 55–73, 70–80, 18–28 and 93–97 % for APRI low cutoff; and 22–49, 81–94, 19–34 and 91–94 % for APRI high cutoff [137].

Emerging technologies utilizing ultrasound and MR imaging platforms, such as acoustic radiation force impulse imaging and diffusion-weighted MR imaging have been developed as well. These approaches make up for the weak points in the liver biopsy by improving the histology results, but they also reduce the need for liver biopsy.

Liver stiffness measurement using TE (Fibroscan^®^) was first developed in 2003 and is the most extensively evaluated method of this type. Following vigorous validations in many studies, TE was shown to be a reliable and accurate surrogate for liver biopsy in assessing the severity of liver fibrosis [138–140]. In recent years, many patients in Asia-Pacific countries have been evaluated by TE, resulting in extensive accumulated experience. The performances of TE in diagnosing significant fibrosis (≥F2 stage) and cirrhosis (F4 stage) are good, with AUROC of 0.81–0.95 and 0.8–0.98, respectively. Most studies report estimated cutoff ranges of 6.3–7.9 and 9.0–13.8 kPa for the diagnosis of significant fibrosis and cirrhosis, respectively. However, although TE has displayed reliable diagnostic accuracy in this setting, it can be influenced by factors such as necroinflammation, edema, food intake, and cholestasis, resulting in an overestimation of TE values. Because of the complex natural history of chronic HBV infection, which frequently presents as fluctuating patterns associated with necroinflammatory activity, serum levels of ALT and bilirubin must be considered as a potential confounder when interpreting the TE values of chronic HBV-infected patients.

Liver fibrosis is a dynamic process. Beyond the cross-sectional studies, recent evaluations of noninvasive tests have focused on their ability to predict the risk of disease progression or liver-related death, and on their use in monitoring the treatment response during long-term, follow-up longitudinal assessments [141, 142]. A major advantage of noninvasive tests is that they allow repeated serial measurements of liver fibrosis. Indeed, the role of noninvasive tests is no longer confined to the detection of the severity of liver fibrosis; rather, noninvasive approaches provide a surveillance tool that predicts clinical outcome and long-term prognosis, thus helping to determine treatment strategies. Furthermore, to improve the overall diagnostic performance, the advantages of combining TE and serum markers have been established in several studies [143–145], but further validation is still required.

Neither noninvasive testing nor liver biopsy alone is sufficient to make a definitive decision in clinical practice, and regardless of specific methodological advances, all of the available clinical and biological data must be taken into account in therapeutic decision-making. The utilization of noninvasive tests for assessing liver histology can significantly reduce, but not completely replace, the need for liver biopsy and should be seen as a complementary tool in the management of chronic HBV-infected patients.

#### Use of risk calculators

Chronic HBV infection remains an important cause of HCC development. HCC causes poor quality of life and shortened survival, and is thus regarded as a major health challenge. The risk of CHB progressing to HCC may be reduced by antiviral therapy [146], and surveillance with abdominal ultrasonography and serum alpha-fetoprotein tests can be used to screen patients for early HCC treatment. Although, the global number of individuals infected with CHB is extensive, especially in endemic areas such as Asian-Pacific and sub-Saharan African regions, only a small number of patients develop end-stage liver diseases. Therefore, the identification and triage of patients who are at high risk of HCC development is important. Several factors, such as gender, age, family history of HCC, presence of hepatic inflammation/fibrosis, alcohol consumption, elevated viral load, hepatitis Be antigen (HBeAg) positivity, and specific HBV genotypes (e.g., genotype C), have been identified to be independently associated with elevated risk of HCC development [13, 67, 147]. These factors, including patient, viral, and environmental factors, interact with one another and lead to HCC development in patients with chronic HBV infection. From the individualized medicine point of view, these factors should be used to reveal the future risk of HCC progression in patients with viral hepatitis so that preventive measures can be applied to those at high risk [148].

##### Risk calculators for HCC in chronic HBV-infected patients without antiviral treatment

Many Asian study groups established prediction models that incorporated several clinical variables to estimate HCC risk for chronic HBV-infected patients. These included IPM from Korea (hospital based using gender, HCV infection, HBV infection, AFP levels, chronic hepatitis, cirrhosis, alcohol use and ALT levels) [149]; GAG-HCC risk score from Hong-Kong (hospital based using gender, age, HBV DNA levels, core promoter mutations and cirrhosis) [150]; CUHK clinical scoring system from Hong-Kong (hospital based using age, albumin, bilirubin, HBV DNA levels and cirrhosis) [151]; and REVEAL nomograms from Taiwan (community based using gender, age, ALT levels, family history of HCC, alcohol consumption, HBV DNA levels, HBeAg and HBV genotype) [152]. The most important issue with these was the lack of external validation. All these groups then collaborated to develop a HCC risk score (REACH-B) incorporating gender, age, serum alanine transaminase (ALT) concentration, HBeAg status, and serum HBV DNA level as the predicting parameters [153]. This study derived a 17-point risk model from 3584 treatment-free and cirrhosis-free CHB patients in a community-based Taiwanese cohort (REVEALHBV), and validated its use in a composite hospital-based cohort (*n* = 1505) from Hong Kong and Korea. This risk score could predict HCC with a wide range of risks, ranging from 0.0 to 23.6 % at 3 years, 0.0 to 47.4 % at 5 years, and 0.0 to 81.6 % at 10 years for patients with the lowest through the highest scores. Although the derivation and validation cohorts were quite different in their distributions of sex, age, HBeAg serostatus, ALT concentration, HBV DNA level, and cirrhosis, the risk score developed from the derivation cohort accurately and reliably estimated the HCC risk at 3, 5 and 10 years of follow-up in the validation cohort. The area under the receiver operating characteristic curve (AUROC) and the corresponding 95 % CI were 0.811 (0.790–0.831), 0.796 (0.775–0.816), and 0.769 (0.747–0.790), respectively, in predicting 3-, 5- and 10-year HCC risk, indicating a fair discriminatory capability. The performance of the risk score was improved when cirrhotic patients were excluded from the validation cohort [153].

With recent studies showing utility of quantitative serum HBsAg levels (which are reproducible and low cost) in providing additional predictability of HCC, especially in patients with low levels of HBV DNA (<2000 IU/ml) [154], the original REVEAL nomograms were upgraded by incorporating qHBsAg into the HCC risk prediction model [155]. In addition to HCC, this study also provided a prediction model for predicting the long-term development of cirrhosis. The risk prediction model for HCC included age, sex, family history of HCC, and a combined variable encompassing HBeAg serostatus, serum HBV DNA and ALT levels, quantitative serum HBsAg level, and HBV genotype as the predicting parameters. The projected 5-, 10-, and 15-year HCC risk for each score was pre-calculated and depicted in a nomogram. This upgraded HCC risk calculator was internally validated using a third of the population from which the model was derived, and showed excellent prediction accuracy and discriminatory ability.

Since serum HBV DNA measurement is relatively expensive compared to all other risk predictors in the risk calculator, a risk calculator might be generated in which quantitative serum HBsAg levels can be used in lieu of serum HBV DNA levels.

The REACH-B scoring system has been used to classify anti-viral treatment eligibility of CHB patients according to the 2012 Asian Pacific Association for the Study of the Liver (APASL) treatment guidelines [156]. In this study, a total of 904 noncirrhotic CHB patients were enrolled, and it showed that for patients to be eligible for anti-viral treatment, the minimal REACH-B score should be 7 and 6, respectively, for HBeAg-seropositive and HBeAg-seronegative patients. Additionally, in HBeAg-seronegative patients, the REACH-B score could predict treatment eligibility, with an adjusted OR (95 % CI) of 1.78 (1.61–1.98). In HBeAg-seropositive patients, however, this same score-dependent eligibility of treatment was not observed. In this study, the authors also showed that the REACH-B score was excellent in discriminating treatment eligibility for young (<40 years) HBeAg-seropositive patients (AUC 0.903) and in both young (<45 years; AUC 0.907) and older (≥45 years; AUC 0.883) HBeAg-seronegative patients; but the discriminatory capability for older (≥40 years) HBeAg-seropositive patients was poor (AUC 0.664). They also found that 46.4 % of HBeAg-seropositive patients older than 40 years of age with high risk of HCC, as estimated by a REACH score ≥11, would be erroneously excluded from treatment, mainly because their ALT levels never exceeded 2× ULN, even after frequent blood tests during follow-up.

These risk calculators can be used for evidence-based decisions during clinical management of chronic HBV-infected patients. Based on patient’s personalized HCC risks, their follow-up intervals, surveillance patterns, and referral strategies can be tailored. Also, timely antiviral therapy in high-HCC-risk patients may lead to improvement in quality of life and prolonged survival. The potential cutoff risk and corresponding management strategies still remain an issue.

Although the risk calculators are easy-to-use and the REACH-B predictive score was externally validated to be an applicable tool for HCC risk estimation, several precautions are warranted. Because surveillance strategies derived from a Taiwanese population might not apply globally, further validation is still needed in patients of different ethnicities, geographical areas, ages at infection, genetic background, HBV genotypes or species, comorbidities, and exposures to environmental factors such as aflatoxin and alcohol [157]. It has been shown that the applicability and predictability of HCCrisk scores developed in Asians are poor or modest in Caucasian CHB patients, for whom different risk scores are required [158].

Since current HCC risk prediction tools were generated from a natural history cohort without history of antiviral therapy, the inference of predicted risks under circumstances of antiviral therapy should theoretically be inappropriate; although these risk calculators have been also used for predicting HCC risk among patients on anti-virals [159].

Besides HCC, several other clinical outcomes and milestones of chronic HBV infection, such as cirrhosis, and liver-related mortality, as well as the seroclearance of HBeAg, HBsAg, and HBV DNA, can also be suitable for the development of risk prediction tools.

3.3 Recommendations (assessment of persons with chronic HBV infection)
  3.3.1 The initial evaluation of an individual with HBV infection should include assessment of the level of viremia, degree of inflammation and the presence and stage of liver disease. A detailed history to investigate the possible source of HBV transmission, as well as physical examination, biochemical tests [including aspartate aminotransferase (AST) and ALT, gamma-glutamyl transpeptidase (GGT), alkaline phosphatase, bilirubin, and serum albumin and globulins, and prothrombin time], complete blood count and hepatic ultrasound should be performed (A1).
  3.3.2 Measurement of HBV DNA is essential for the diagnosis, assessment for initiating treatment and subsequent monitoring of infected subjects (A1).
  3.3.3 Other causes of chronic liver disease should be looked for, including coinfections with HDV, HCV and/or HIV (A1).
  3.3.4 Comorbidities, including alcoholic, autoimmune, metabolic liver disease with steatosis or steatohepatitis should be assessed (A1).
  3.3.5 Accurate assessment of the degree of fibrosis is essential not only to determine prognosis, but also to identify patients who require antiviral treatment (AI).
  3.3.6 A liver biopsy is recommended to determine the stage of fibrosis and/or the grade of activity in patients with a high viral load and high-normal or minimally raised ALT levels without clinical evidence of cirrhosis (AI).
  3.3.7 Noninvasive tests such as transient elastography can be a useful, reliable and practical tool for the diagnosis, and for decision-making for treatment and monitoring clinical outcome (BI).
  3.3.8 Transient elastography is especially useful in the assessment of liver fibrosis in patients with normal ALT and bilirubin levels (AI). In a patient infected with hepatitis B, a liver stiffness measurement <6 generally excludes a significant liver disease, above 8 indicates significant fibrosis (F ≥2 by METAVIR fibrosis score) and above 11 raises suspicion of cirrhosis. These cutoffs may have regional and population variations (A1).
  3.3.9 Risk calculators may be used to assess HCC risk in chronic HBV-infected patients and make decisions to manage such patients (B2).
  3.3.10 Specific risk calculators need to be developed and validated in patients of different ethnicities, geographical areas, ages at infection, genetic backgrounds, HBV genotypes, comorbidities, and exposures to environmental factors such as aflatoxin and alcohol (B1).

### 3.4 Goals and endpoints of therapy in chronic HBV infection

#### Goal of therapy

The ultimate goal is global eradication of HBV infection by various strategies, including vaccination, treatment and prevention of transmission. The goal of therapy for chronic HBV infection is to improve quality of life and survival of the infected person by preventing progression of the disease to cirrhosis, decompensated cirrhosis, end-stage liver disease, HCC and death; and prevention of transmission of HBV to others. This goal can be achieved if HBV replication can be suppressed in a sustained manner. Then, the accompanying reduction in histological activity of CHB lessens the risk of cirrhosis and decreases the risk of HCC, particularly in noncirrhotic patients. However, chronic HBV infection cannot be completely eradicated due to the persistence of covalently closed circular DNA (cccDNA) in the nucleus of infected hepatocytes, and also, the HBV genome integrates into the host genome and might favour oncogenesis and the development of HCC [160].

#### Endpoints of therapy

Therapy must ensure a degree of virological suppression that will lead to biochemical remission, histological improvement and prevention of complications. The ideal endpoint in both HBeAg-positive and HBeAg-negative patients is sustained off-therapy HBsAg loss, with or without seroconversion to anti-HBs. This is associated with a complete and definitive remission of the activity of CHB and an improved long-term outcome. This endpoint, however, is infrequently achievable with the currently available anti-HBV agents. A more realistic endpoint is the induction of sustained or maintained virological remission [25]. Induction of sustained off-therapy virological response in both HBeAg-positive (with sustained anti-HBe seroconversion) and HBeAg-negative patients is a satisfactory endpoint, because it has been shown to be associated with improved prognosis. If sustained off-therapy response not achievable, then a maintained virological remission (undetectable HBVDNA by a sensitive PCR assay) under long-term antiviral therapy in HBeAg-positive patients who do not achieve anti-HBe seroconversion and in HBeAg-negative patients is the next most desirable endpoint.

Health-related quality of life (HRQOL) is significantly affected in CHBV patients, particularly in those with more severe forms of the disease. Prevention of disease progression with early treatment or liver transplantation can certainly improve HRQOL. Even though some antiviral medications decrease HRQOL during the acute treatment period, the HRQOL of CHBV patients improves after completion of antiviral treatment [161]. In order to improve HRQOL of CHB patients, attention should be paid to the reduction of patients’ treatment cost burden and the provision of early health education accompanied with proper treatments [162]. A recent Chinese study evaluated the effect of comprehensive intervention on health-related quality of life and provided guidance on improving HRQOL for patients with CHB. Comprehensive intervention included government support, technical guidance from the Chinese Center for Disease Control and Prevention, standardized medical care, and community involvement. HRQOL before and 1 year after intervention was measured with the Short Form 36 and HBV-specific health surveys. After comprehensive intervention, the HRQOL in patients with CHB showed significant improvements in body pain, vitality, social functioning, and mental as well as physical and mental component score (*p* < 0.05). Family and social support increased, and financial concerns decreased (*p* < 0.05) [163].

3.4 Recommendations: goals and endpoints of therapy in chronic HBV infection
  3.4.1 The overall goal is global eradication of HBV infection by various strategies including vaccination, treatment and prevention of transmission (A1).
  3.4.2 The goal of therapy for CHB is to improve quality of life and survival of the infected person by preventing development of disease, progression of the disease to cirrhosis, decompensated cirrhosis, end-stage liver disease, HCC and death; and by prevention of transmission of HBV to others (A1).
  3.4.3 The ideal endpoint in both HBeAg-positive and HBeAg-negative patients is sustained off-therapy HBsAg loss, with or without seroconversion to anti-HBs (A1).
  3.4.4 Induction of sustained off-therapy virological response in both HBeAg-positive (with sustained anti-HBe seroconversion) and HBeAg-negative patients is a satisfactory endpoint (A1).
  3.4.5 If sustained off-therapy response is not achievable, then a maintained virological remission (undetectable HBV DNA by a sensitive PCR assay) under long-term antiviral therapy in HBeAg-positive patients who do not achieve anti-HBe seroconversion, and in HBeAg-negative patients, is the next most desirable endpoint (A1).

### 3.5 Indications of therapy in chronic HBV infection

The indications for treatment are generally based mainly on the combination of three criteria: serum HBV DNA levels, serum ALT levels and severity of liver disease (assessed by clinical evaluation, liver biopsy or noninvasive methods). Indications for treatment should also take into account age, health status, family history of HCC or cirrhosis and extrahepatic manifestations (Table 5).

**Table 5:** Treatment indications for chronic HBV-infected patients

HBsAg positive patient	HBV DNA (IU/ml)	ALT	Treatment
Decompensated cirrhosis	Detectable	Any	Treat. Histology not needed. Consider LT of no stabilization
Compensated cirrhosis	>2000	Any	Treat. Histology should be obtained or assess fibrosis noninvasively^a^
Severe reactivation of chronic HBV	Detectable	Elevated	Treat immediately
Noncirrhotic HBeAg-positive chronic hepatitis B	>20,000	>2× ULN	Observation for 3 months if no hepatic decompensation concerns. Treat. Histology should be obtained or assessed noninvasively^a^
		1–2× ULN	Assess fibrosis noninvasively. Monitor every 3 months. Biopsy if noninvasive tests suggest evidence of significant fibrosis, ALT is persistently elevated, age >35 years or family h/o HCC or cirrhosis. Treat if moderate to severe inflammation or significant fibrosis^a^
		Persistently normal (age <30) (immune tolerant phase)	Assess fibrosis noninvasively. Monitor every 3 months. Biopsy if noninvasive tests suggest evidence of significant fibrosis, or there is a family h/o HCC or cirrhosis. Treat if moderate to severe inflammation or significant fibrosis^a^
	2000–20,000	Any ALT	Rule out other causes of elevated ALT.Assess fibrosis noninvasively. Monitor every 3 months. Biopsy if noninvasive tests suggest evidence of significant fibrosis, age >35 years, ALT is persistently elevated, or there is a family h/o HCC or cirrhosis. Treat if moderate to severe inflammation or significant fibrosis^a^
	<2000	<ULN	Assess fibrosis noninvasively. Monitor every 3 months. Biopsy if ALT becomes elevated, noninvasive tests suggest evidence of significant fibrosis, age >35 years or with family h/o HCC or cirrhosis. Treat if moderate to severe inflammation or significant fibrosis
		>ULN	Rule out other causes of elevated ALT. Assess Fibrosis noninvasively. Monitor every 3 months. Biopsy if noninvasive tests suggest evidence of significant fibrosis, ALT is persistently elevated, age >35 years or with family h/o HCC or cirrhosis. Treat if moderate to severe inflammation or significant fibrosis^a^
Noncirrhotic HBeAg-negative chronic hepatitis B	>2000	>2× ULN	Observation for 3 months if no hepatic decompensation concerns. Treat. Histology should be obtained or assess fibrosis noninvasively
		1–2× ULN	Rule out other causes of elevated ALT.Assess fibrosis noninvasively. Monitor every 3 months. Biopsy if noninvasive tests suggest evidence of significant fibrosis, age >35 years, ALT is persistently elevated, or there is a family h/o HCC or cirrhosis. Treat, if moderate to severe inflammation or significant fibrosis^a^
		Persistently normal	Assess fibrosis noninvasively. Monitor every 3 months. Biopsy if ALT becomes elevated, noninvasive tests suggest evidence of significant fibrosis, age >35 years or with family h/o HCC or cirrhosis. Treat if moderate to severe inflammation or significant fibrosis^a^
	<2000	>ULN	Rule out other causes of elevated ALT. Assess fibrosis noninvasively. Monitor every 3 months. Biopsy, if noninvasive tests suggest evidence of significant fibrosis, ALT is persistently elevated, age >35 years or with family h/o HCC or cirrhosis. Treat if moderate to severe inflammation or significant fibrosis^a^
		Persistently normal	Assess Fibrosis noninvasively. Monitor ALT every 3–6 months and/or DNA every 6–12 months. Biopsy if noninvasive tests suggest evidence of significant fibrosis, ALT becomes elevated, age >35 years or with family h/o HCC or cirrhosis. Treat if moderate to severe inflammation or significant fibrosis^a^

^a^Moderate to severe inflammation on liver biopsy means either hepatic activity index by Ishak activity score >3/18 or METAVIR activity score A2 or A3; significant fibrosis means F ≥2 by METAVIR fibrosis score or Ishak fibrosis stage ≥3. Significant fibrosis by noninvasive markers means liver stiffness ≥8 kPa (by Fibroscan) or APRI ≥1.5. Cirrhosis by noninvasive markers means liver stiffness ≥11 kPa (by Fibroscan) or APRI ≥2.0

**Fig. 1:**
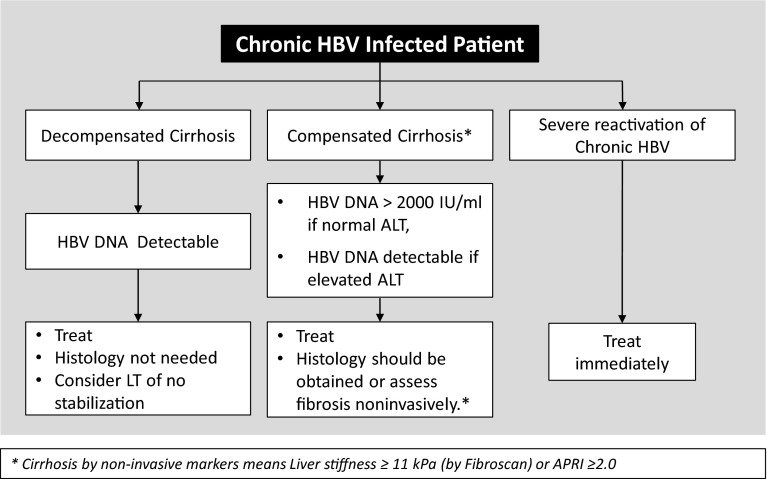
Treatment indications for chronic HBV-infected patients with cirrhosis or reactivation of chronic HBV infection

Patients with decompensated cirrhosis and detectable HBV DNA require urgent antiviral treatment with NA(s). Significant clinical improvement can be associated with control of viral replication [164, 165]. However, antiviral therapy may not be sufficient to rescue all decompensated patients and they should be considered for liver transplantation at the same time (Fig. 1).

Patients with compensated cirrhosis and HBV DNA >2000 IU/ml should also be considered for treatment even if ALT levels are normal. Liver biopsy is recommended, but noninvasive assessment of fibrosis is another option (Fig. 1).

Treatment may be started in pre-cirrhotic chronic HBV-infected patients if they have persistently elevated ALT levels >2 times the upper limit of normal (ULN) (at least 1 month between observations) and HBV DNA >20,000 IU/ml if HBeAg positive and >2000 IU/ml if HBeAg negative. In such patients, liver biopsy may provide additional useful information, especially in those with doubtful causes of hepatic necroinflammation. A noninvasive method for the estimation of the extent of fibrosis is useful in patients who start treatment without liver biopsy.

There is lack of sufficient data to start antiviral therapy in the sub-groups of patients where there is significant fibrosis, but the ALT levels are normal or minimally elevated or the DNA levels are below the defined limits. These group of patients are not uncommon and the experts deliberated on the treatment options for them. It was unanimously agreed that these patients do merit antiviral therapy, in order to prevent further progression of fibrosis and other complications of liver disease. In addition, therapy might help in stabilizing their disease or even regression of fibrosis. In these cases, serial noninvasive assessment of fibrosis and bio-chemical assessment of inflammation and disease severity should be done.

Patients with a rising trend in ALT or bilirubin may be developing an exacerbation, and even severe hepatitis or hepatic decompensation. They should be monitored closely with weekly or biweekly serum ALT, bilirubin, and prothrombin time measurement. Such exacerbations, particularly in patients with declining serum HBV DNA level, may also precede spontaneous HBeAg seroconversion, and may be followed by disease remission. Thus, it is reasonable to delay treatment for an observation period of 3 months, if there is no concern about hepatic decompensation.

Patients with severe reactivation of chronic HBV infection [reactivation with the presence of coagulopathy with prolonged prothrombin time (prolonged by more than 3 s) or INR increased to >1.5] with impending or overt hepatic decompensation should be treated immediately with antiviral agents to prevent the development or deterioration of hepatic decompensation (see “Treatment of patients with reactivation of chronic HBV infection including those developing acute on chronic liver failure” section) (Fig. 1).

Available information suggests that patients with persistently normal alanine aminotransferase levels (PNALT) or minimally raised ALT levels (1–2 times the ULN) respond poorly, in terms of HBeAg seroconversion, when treated with currently available drugs. A recent article evaluating the effects of tenofovir disoproxil fumarate (TDF) in HBeAg-positive patients with normal levels of ALT and high levels of HBV DNA in a double-blinded way was reported. The authors demonstrated that both TDF monotherapy and the combination of TDF and emtricitabine are effective in the suppression of HBV DNA in patients with normal ALT and high viral load. However, only 5 % of patients achieved HBeAg seroconversion after 192 weeks of therapy with combination of TDF and emtricitabine [166]. Therefore, no drug treatment is recommended for this group of patients unless they have evidence of significant fibrosis, cirrhosis, or are under a protocol. One recent meta-analysis showed that nearly half (48 %) of the 683 CHB patients with minimally increased ALT levels (levels 1–2 times the ULN) from nine recruited studies had stage 2 or higher fibrosis (95 % CI 36–61 %). A subgroup of HBeAg-positive and HBeAg-negative patients showed similar rates of fibrosis (41 vs. 47 %; *p* = nonsignificant) [167]. Another study tried to explore the hepatic histological changes after long-term antiviral therapy in CHB patients with persistently normal ALT levels and advanced hepatic fibrosis [168]. The authors compared paired liver biopsies before and after lamivudine treatment in CHB and normal ALT levels. Of them, 82.4 % of patients had a baseline fibrosis score of 4 by Scheuer scoring system and this was reduced to 17.6 % after a median duration of 44.5 months of therapy.

**Fig. 2:**
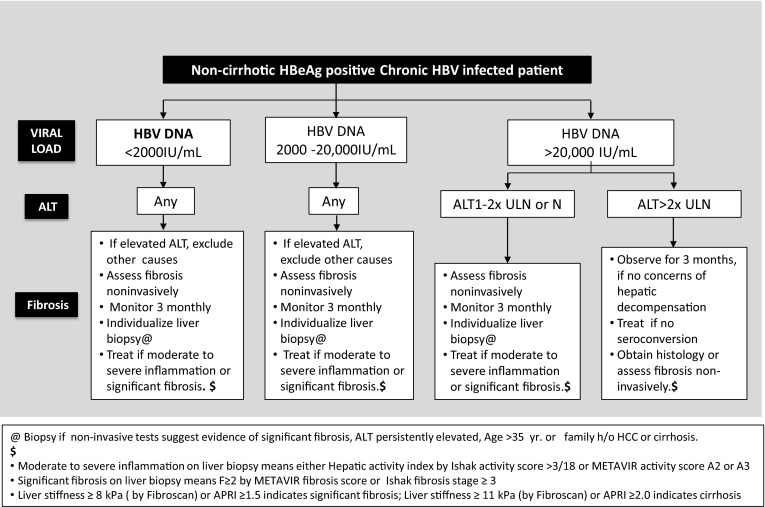
Treatment indications for noncirrhotic HBeAg-positive chronic HBV-infected patients

If patients are not considered for treatment, they should be followed up every 3–6 months. HBeAg-positive patients with serum HBV DNA >20,000 IU/ml and PNALT should also be followed up every 3 months. A liver biopsy should be considered in viremic patients older than 35–40 years, especially those with high normal or minimally raised ALT levels or family history of HCC or cirrhosis, with intent to identify the group of patients with significant fibrosis requiring treatment (Fig. 2).

Patients with active HBV replication (HBV DNA >2000 IU/ml) and minimally elevated (1–2× ULN) or persistently normal ALT should have liver fibrosis assessed. Liver biopsy may be needed before therapy to assess the necroinflammatory grade, determine the fibrotic stage, and exclude other possible causes of raised ALT levels as a guide for consideration of antiviral treatment. Treatment should be instituted if moderate to severe hepatic necroinflammation or significant fibrosis is found. If liver biopsy is not feasible, noninvasive assessment of liver fibrosis should be considered as an alternative.

Immunotolerant patients need special attention. HBeAg-positive patients under 30 years of age with persistently normal ALT levels and a high HBV DNA level, without any evidence of liver disease and without a family history of HCC or cirrhosis, generally do not require immediate therapy. In these cases, noninvasive assessment of liver fibrosis should be done. Follow-up should be done at least every 3–6 months. A liver biopsy should be considered if significant fibrosis is suspected or if there is family history of HCC or cirrhosis.

**Fig. 3:**
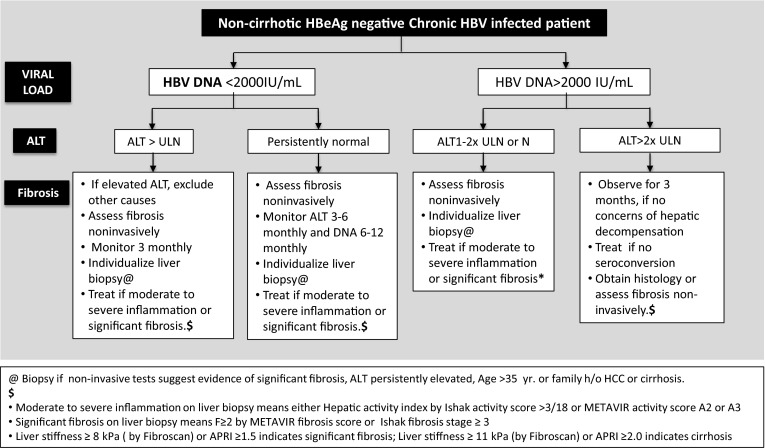
Treatment indications for noncirrhotic HBeAg-negative chronic HBV-infected patients

HBeAg-negative patients with persistently normal ALT levels (ALT determinations every 3 months for at least 1 year) and HBV DNA levels below 2000 IU/ml, without any evidence of liver disease, do not require immediate therapy. Evaluation of the severity of fibrosis by a noninvasive method might be useful as the first screening test in such cases. A suspicion of significant fibrosis should help identify patients for liver biopsy. There is however, limited data using such an algorithmic approach in CHB. Follow-up with ALT and alpha-fetoprotein determinations every 3–6 months and ultrasonography and/or HBV DNA every 6–12 months is needed (Fig. 3).

3.5 Recommendations: indications of therapy in chronic HBV infection
  3.5.1 HBsAg positive patients with decompensated cirrhosis and detectable HBV DNA require immediate antiviral treatment with NA(s). Liver transplantation should be considered if patients do not stabilize with medical management (A1).
  3.5.2 Patients with compensated cirrhosis and HBV DNA >2000 IU/ml should be considered for treatment even if ALT levels are normal (A1). Patients with compensated cirrhosis should be treated irrespective of the ALT and HBV DNA levels (C2).
  3.5.3 Patients with suspected severe reactivation [reactivation with the presence of coagulopathy with prolonged prothrombin time (prolonged by more than 3 s) or INR increased to >1.5] of chronic HBV infection should be started on antiviral therapy immediately after sending tests for quantitative HBV DNA, but without waiting for the results (B1).
  3.5.4 Treatment may be started in pre-cirrhotic chronic HBV-infected patients if they have persistently elevated ALT levels >2 times upper limit of normal (ULN) (at least 1 month between observations) and HBV DNA >20,000 IU/ml if HBeAg positive and >2000 IU/ml if HBeAg negative (B1).
  3.5.5 Patients with high HBV DNA levels (>20,000 IU/ml if HBeAg positive and >2000 IU/ml if HBeAg negative) but ALT <2× ULN should obtain assessment of fibrosis noninvasively, and should be monitored every 3 months. Biopsy should be considered if noninvasive tests suggest evidence of significant fibrosis, ALT becomes persistently elevated, if age is >35 years or there is family h/o HCC or cirrhosis. They should be considered for treatment if biopsy shows moderate to severe inflammation or significant fibrosis (B1).
  3.5.6 HBeAg-positive patients with HBV DNA <20,000 IU/ml, should be evaluated for other causes if ALT is elevated, should obtain assessment of fibrosis noninvasively, and should be monitored every 3 months. Biopsy should be considered if noninvasive tests suggest evidence of significant fibrosis, ALT becomes persistently elevated, if age is >35 years or there is family h/o HCC or cirrhosis. They should be considered for treatment if biopsy shows moderate to severe inflammation or significant fibrosis (B1).
  3.5.7 HBeAg-negative patients with HBV DNA <2000 IU/ml, should be evaluated for other causes if ALT is elevated, should obtain assessment of fibrosis noninvasively, and should be monitored every 3 months if ALT is elevated (if ALT is normal, monitoring should be done with ALT every 3–6 months and with DNA every 6–12 months). Biopsy should be considered if noninvasive tests suggest evidence of significant fibrosis, ALT remains persistently elevated, if age is >35 years or there is family h/o HCC or cirrhosis. They should be considered for treatment if biopsy shows moderate to severe inflammation or significant fibrosis (C1). More long-term data using antiviral therapy is needed for these groups of patients.
  3.5.8 Noninvasive methods for the estimation of the extent of fibrosis are useful in selecting patients for liver biopsy. Patients with the suggestion of significant fibrosis by noninvasive markers [mean liver stiffness ≥8 kPa (by Fibroscan) or APRI ≥1.5] should be considered for liver biopsy followed by treatment, if biopsy shows moderate to severe inflammation or significant fibrosis (C1) (Table 5). Patients with suspected significant fibrosis but unwilling to undergo liver biopsy may be considered for treatment (C2) or should be kept on regular follow-up (B1).
  3.5.9 Patients who are not considered for treatment should be followed up regularly by measurement of ALT levels, HBV DNA, AFP, ultrasonography and fibrosis assessment (Table 5) (B1).

### 3.6 Results of currently available therapies, predictors of response to therapy, follow-up and stopping rules during therapy in chronic HBV infection

#### 3.6.1 Results of and predictors of response to nucleos(t)ide analogues

Lamivudine, adefovir dipivoxil, entecavir, telbivudine and tenofovir disoproxil fumarate have been approved in most Asia Pacific countries. Clevudine has been approved in Korea and the Philippines, while its development has been stopped in others countries due to myopathy.

##### l-Nucleoside analogues

###### *Lamivudine*

In the Asian lamivudine (LAM) trial and a multi-center trial in China, HBeAg seroconversion was achieved in approximately 44–47 % after 4–5 years of therapy [169]. In a long-term follow-up study among 95 CHB patients (43 HBeAg-positive) on lamivudine for at least 10 years with maintained viral suppression (HBV DNA <2000 IU/ml), seven (10 %) patients had HBsAg seroclearance. Baseline HBsAg <1000 IU/ml and on-treatment reduction of HBsAg >0.166 log IU/ml were optimal cutoffs to predict HBsAg seroclearance (negative predictive values 98.1 and 97.8 %, respectively), but in general, the HBsAg decline was slow at 0.104 log IU/ml/year [170].

In a Korean study including 178 patients with HBeAg seroconversion and discontinued lamivudine, the relapse (defined as HBV DNA >140,000 copies/ml) rate after 12-month consolidation was 8.7 % in 5 years, in contrast to 61.9 % in those with consolidation therapy <12 months [171]. In another study including 101 patients from Taiwan and Hong Kong, longer consolidation of lamivudine was associated with a higher combined response (HBeAg seroconversion and undetectable HBV DNA) 6 months post-treatment; 25.6, 39.0 and 71.4 % with consolidation therapy for <12, 12–18 and >18 months, respectively [172]. A study among 83 Taiwanese patients found that HBsAg level <300 IU/ml at the end of lamivudine treatment could predict HBsAg seroclearance after stopping lamivudine (five of nine patients, 55.5 %) at a median follow-up of 4 years [173]. More data is needed for the use of HBsAg level to guide treatment cessation.

In HBeAg-negative patients, studies among Chinese patients who stopped LAM after a minimum of 24 months of treatment with at least three results of undetectable HBV DNA 6 months apart showed a post-treatment relapse (HBV DNA ≥10^4^ copies/ml) rate of 37–50 % at 1 year [174, 175]. A study from Hong Kong including 53 HBeAg-negative patients treated with LAM for a mean of 34 (12–76) months and stopped LAM therapy for 47 ± 35 months showed that end-of-treatment HBsAg ≤100 IU/ml plus reduction by >1 log from baseline could predict sustained response (HBV DNA ≤200 IU/ml) of 100 % (five of five patients) at 12 months and HBsAg loss at 5 years post-treatment [176]. Another Taiwanese study including 107 HBeAg-negative patients treated by LAM for 93 ± 35 months showed that end of treatment HBsAg <120 and <200 IU/ml were associated with HBsAg loss (19 of 24 patients, 79.2 %) and sustained response (HBV DNA <2000 IU/ml; 28 of 30, 93.3 %) at a median of 4 years post-treatment [177].

LAM is well tolerated, even in patients with decompensated cirrhosis or in pediatric patients [178]. The key LAM resistant mutant is at the YMDD locus in the catalytic domain of the HBV polymerase gene (rtM204I/V/S), which may confer cross-resistance to other drugs in the l-nucleoside group, such as telbivudine and entecavir. The compensatory mutation, rtL180M, is frequently associated with rtM204V/S and will reduce the susceptibility to entecavir. Another LAM resistant mutation, rtA181T/V, may confer cross-resistance to adefovir and telbivudine, and has partial resistance to tenofovir. Compensatory codon substitutions that increase viral replication may also be found, such as rtL80V/I, rtV173L, rtT184S/G [179]. The incidence of rtM204V/I substitution increased from 24 % in 1 year to 70 % in 5 years. Undetectable HBV DNA at week 24 was associated with 9 and 5 % of LAM resistance at 2 years among HBeAg-positive and HBeAg-negative patients, respectively [180].

Although prolonged lamivudine (LAM) therapy is associated with the emergence of LAM-resistant mutations, it is still a commonly used therapy in many Asian countries because of its established long-term safety and low cost. In one recent multicenter study on 838 patients, an individual prediction model for lamivudine treatment response in HBeAg-positive CHB patients was suggested. In the multivariate analysis, age [odds ratio (OR) 0.974, *p* < 0.001], baseline alanine aminotransferase level (OR 1.001, *p* = 0.014), and baseline HBV DNA level (OR 0.749, *p* < 0.001) were independent factors for HBeAg seroconversion. Based on the predictors, an IPM was established. Patients were classified into high (>50 %), intermediate (30–50 %), or low (≤30 %) response groups based on their probability of HBeAg seroconversion according to the IPM. The cumulative HBeAg seroconversion rate at 6 years for the high, intermediate, and low response groups was 66.0, 48.5, and 21.8 %, respectively (*p* < 0.001). This model may allow screening of LAM responders prior to the commencement of antiviral treatment, but needs further validation [181].

###### *Telbivudine*

Telbivudine (LdT) 600 mg daily has been shown to have more potent HBV DNA suppression than LAM and ADV [182, 183]. After excluding patients who had drug resistance at year 2 in the GLOBE study, continuation of LdT until year 4 was associated with undetectable HBV DNA in 76 % of HBeAg-positive and 86 % of HBeAg-negative patients, HBeAg seroconversion in 53 % of HBeAg-positive patients, and HBsAg loss in 1.9 % of HBeAg-positive patients and 0.6 % in HBeAg-negative patients [184]. Among the 61 patients who had telbivudine stopped because of HBeAg loss for >6 months and HBV DNA <5 log copies/ml (98 % had HBV DNA <300 copies/ml), 50 (82 %) had sustained HBeAg seroconversion, 28 (46 %) had HBV DNA <4 log copies/ml (14 patients had undetectable HBV DNA), and four (6.5 %) had HBsAg loss [184].

The most common LdT resistant substitution is rtM204I, and rtA181T/V [179]. The 2-year risk of LdT resistance was 25.1 % in HBeAg-positive patients and 10.8 % in HBeAg-negative patients, which is lower than that of lamivudine [180]. In the subgroup that had no genotypic resistance at year 2 and received LdT up to year 4, the cumulative virological breakthrough/resistance rate was 18.8/10.6 % for HBeAg-positive and 15.9/10.0 % for HBeAg-negative patients [184].

In a multi-centered Chinese study among HBeAg-positive patients on LdT, patients who had HBV DNA ≥300 copies/ml at week 24 were randomized to add-on adefovir treatment versus continuation of telbivudine until week 104. The add-on adefovir group had a higher chance of HBV DNA <300 copies/ml (76.7 vs. 61.2 %), a lower risk of genotypic resistance (2.7 vs. 25.8 %) and comparable rate of HBeAg seroconversion (23.7 vs. 22.7 %) compared to the continued LdT group at week 104 [185]. In a real-life cohort in Hong Kong, among the 25 patients who had detectable HBV DNA but <2000 IU/ml after 6–12 months of telbivudine, 24 (96 %) could achieve undetectable HBV DNA after switching to entecavir for a median follow-up of 2 years [186]. LdT is generally well tolerated, including in patients with decompensated liver cirrhosis [178]. Based on the databases of the GLOBE study as well as other studies including compensated and decompensated patients, LdT was found to improve renal function, as measured by calculated eGFR after 24 weeks of therapy, and this benefit was seen among patient who were aged >50 years and those with eGFR ≤90 at baseline [187]. The improvement in eGFR was confirmed in another Korean study with 43 patients on LdT and adefovir combination therapy for 24 weeks [188]. Among patients who received LdT for 4 years, creatine kinase increase was reported in 10.1 % of patients and muscle symptoms in 6.1 % of patients (myopathy and myositis in 0.6 %) [187].

##### Acyclic nucleotide phosphonates

###### *Adefovir dipivoxil*

In HBeAg-positive patients, HBeAg seroconversion can be achieved in 30–37 % after 3–5 years of adefovir (ADV) treatment [189, 190]. In HBeAg-negative patients, 67 % of patients had HBV DNA <200 IU/ml and 75 % had fibrosis regression after 240-week treatment with ADV [191].

The safety profile of 10 mg ADV daily was similar to placebo in patients with compensated CHB. Reversible increase in serum creatinine of more than 0.5 mg/dl (maximum 1.5 mg/dl) was reported in up to 3 % of patients when the therapy is extended to 5 years [191].

The primary drug resistance mutations against ADV are rtA181V/T and rtN236T. The cumulative incidence of genotypic resistance to ADV was 29 % after 5 years of therapy in HBeAg-negative patients [191]. The substitution rtN236T has partial cross-resistance to tenofovir, but it is sensitive to LAM, LdT and entecavir [179].

ADV is effective in suppressing HBV DNA in patients with rtM204I/V HBV substitution. In a 5-year follow-up cohort of 165 LAM-resistant patients, add-on ADV therapy resulted in undetectable HBV DNA in 74 % and genotypic ADV resistance in 10.2 % of patients [192]. Undetectable HBV DNA at month 6 is the best predictor of maintained HBV DNA suppression; 87–100 % of patients with undetectable HBV DNA at month 6 had undetectable HBV DNA at 3–5 years on continuous ADV add-on therapy [193, 194].

###### *Tenofovir disoproxil fumarate*

Tenofovir disoproxil fumarate (TDF) is an acyclic adenine nucleotide analogue effective for both HBV and HIV. Five-year continuous TDF therapy was associated with HBV DNA <400 copies/ml in 65 % of HBeAg-positive and 83 % of HBeAg-negative patients; HBeAg seroconversion in 40 % and HBsAg loss in 10 % (all but one were HBeAg-positive; 96 % HBV genotype A and D) patients [195]. On paired liver biopsy at 5 years, 87 % of the 348 patients had histological improvement and 74 % of the 96 cirrhotic patients had regression of liver cirrhosis [195]. Patients who had high viral load (>9 log copies/ml) took a longer time to reach HBV DNA <400 copies/ml than those with lower baseline HBV DNA levels, but overall, 96.9 % of patients who completed 240 weeks of therapy could achieve HBV DNA <169 copies/ml [196]. Among immune-tolerant patients (HBeAg-positive, HBV DNA >1.7 × 10^7^ IU/ml, normal ALT), a combination of tenofovir with emtricitabine was associated with a higher rate of undetectable HBV DNA than tenofovir monotherapy (76 vs. 55 %) after treatment for 4 years, but the overall rate of HBeAg seroconversion was only 5 % (all in patients on combination therapy) [166]. Among the 52 patients who stopped treatment after 4 years, 51 of them had rapid increase in HBV DNA within 4 weeks and one patient had an ALT flare within 24 weeks.

TDF is generally well tolerated, including in patients with decompensated liver disease [165]. Reduction of creatinine clearance to <50 ml/min is extremely uncommon among patients with normal baseline renal function (<1 %) after 3–5 years of continuous TDF treatment [197, 198]. Approximately 1 % of patients developed hypophosphatemia (<2 mg/dl or 0.65 mmol/l), and most of them resolved without dosage modification, treatment interruption or phosphate supplementation. In a multi-centered study comparing TDF (*n* = 141) with TDF and emtricitabine (*n* = 139) in lamivudine-resistant CHB, there was a small decline in the bone mineral density of the spine (−1.4 %) and hip (−1.8 %) at week 96 of treatment [198]. Rare cases of Fanconi syndrome that readily resolved with cessation of tenofovir have been reported [199].

No TDF resistance has been reported up to 7 years [200]. Tenofovir monotherapy is sufficient in the treatment of rtM204V/I ± rtL180M HBV variants; 89.4 % patients on TDF versus 86.3 % patients on a combination of TDF and emtricitabine achieved undetectable HBV DNA (<69 IU/ml) after 96 weeks of treatment [198]. In vitro studies showed that a single mutation of the ADV resistant mutations, A181T/V or N236T, had little reduced susceptibility to TDF. On the other hand, presence of the double mutant rtA181V/T + rtN236T had low level, reduced susceptibility to TDF [201]. In a post hoc analysis of a multi-center study comparing TDF versus TDF and emtricitabine combination among ADV refractory patients, patients with rtN236T showed a similar decline in HBV DNA as of those with wild-type HBV in the initial 24 weeks by either regime [202]. Another European multi-center study showed that TDF monotherapy and TDF/emtricitabine combination were equally effective in suppressing the HBV DNA to <400 copies in 168 weeks (82 and 84 %, respectively) among ADV refractory patients, and there was no difference in the response with regard to the baseline LAM/ADV resistance profile [197]. In a case series of 57 patients who failed to achieve complete HBV DNA suppression by antiviral drugs including entecavir or TDF due to the presence of multi-drug resistant HBV, a combination of TDF and entecavir (0.5 mg for naïve or 1 mg for LAM experienced patients daily) could achieve undetectable HBV DNA (<80 IU/ml) in 90 % of patients after treatment for a median of 21 months [203].

#### d-Cyclopentanes

##### Entecavir

Entecavir (ETV) is a cyclopentyl guanosine analogue with potent selective inhibition of the priming, DNA-dependent synthesis, and reverse transcription functions of HBV polymerase. In Asian cohorts treated with ETV 0.5 mg daily, approximately 83–92 % patients had undetectable HBV DNA, 26–49 % patients had HBeAg seroconversion and <1 % of patients had HBsAg seroclearance at year 3 of treatment [204, 205]. Among 222 treatment-naïve patients treated with entecavir in Hong Kong, 97.1 % patients had undetectable HBV DNA, 66.9 % had HBeAg seroconversion and only one patient achieved HBsAg seroclearance after 5 years [206]. The rate of HBsAg decline is approximately 0.125 log IU/ml/year, which explains the need for long-term therapy and low rate of HBsAg clearance in ETV-treated patients [206]. Among HBeAg-positive patients with high viral load (>10^8^ IU/ml), a combination of tenofovir and entecavir could achieve a higher rate of undetectable HBV DNA than entecavir monotherapy at week 96 (78.8 vs. 62.0 %, respectively) [207]. However, this study lacks the tenofovir monotherapy arm for comparison.

 In a Korean study, approximately 14–16 % of treatment-naïve patients had primary nonresponse as defined by AASLD (<2 log reduction in HBV DNA at month 6) or EASL (<1 log reduction in HBV DNA at month 3), but all primary non-responders could achieve undetectable HBV DNA after 54 months of treatment [208]. On the other hand, partial virological response (detectable HBV DNA at month 12) was predictive of a lower probability of complete HBV DNA suppression and higher risk of virological breakthrough. Approximately 18–26 % of treatment-naïve patients had partial virological response on entecavir; the cumulative rate of virological response (undetectable HBV DNA) at year 3 is 45–58 % and virological breakthrough is 5.1–6.3 % [205, 209]. For some of these patients, virological breakthrough might be related to poor drug adherence.

Long-term cohort studies among entecavir-treated patients compared with historic untreated controls in Japan and Hong Kong demonstrated reduction in mortality, liver-related complication and HCC, especially among patients with liver cirrhosis [210–212]. Patients who achieved undetectable HBV DNA during treatment had better prognosis [213, 214]. Over 97 % of treatment-naïve patients could achieve maintained HBV DNA suppression on entecavir after 2–3 years, while most patients who failed to achieve undetectable HBV DNA were exposed to previous antiviral agents [214]. Among patients who failed to have complete HBV DNA suppression with entecavir, switching or add-on tenofovir was associated with 97–100 % undetectable HBV DNA after 12 months [215, 216].

In a retrospective Taiwanese study among 95 HBeAg-negative patients who discontinued ETV therapy after undetectable HBV DNA had been documented on three occasions, each 6 months apart, the cumulative clinical relapse (ALT >2 time upper limit of normal and HBV DNA >2000 IU/ml) was 45.3 % in 1 year [217]. Nine patients had spontaneous remission while the remaining 34 patients were retreated by ETV with good HBV DNA suppression. In another prospective study from Hong Kong, ETV was stopped in 184 HBeAg-negative patients, fulfilling the same stop treatment criteria. The cumulative rate of virological relapse (HBV DNA >2000 IU/ml) was 72.4 % at 6 months and 91.2 % at 1 year; 25.8 % of patients had elevated ALT level before ETV retreatment was recommenced [218]. No baseline or on-treatment factors were found to be consistently predictive of post-treatment relapse after stopping ETV.

ETV is well tolerated. The US Food and Drug Administration (FDA) requires all approved NAs to carry the “black box” warning for the potential development of lactic acidosis as a result of mitochondrial toxicity. Most of the reports of lactic acidosis for LAM and TDF have been when they were used in combination with other antiretroviral agents in HIV-infected patients. Isolated cases have been reported for TEL and ADV in HBV patients [219, 220]. Reports of cases have also been observed in patients treated with ETV, in particular those with impaired liver function and high model for end-stage liver disease (MELD) score [221, 222]. Interestingly, only the MELD and not the Child–Pugh score was correlated with the development of lactic acidosis, suggesting that renal impairment may be an important contributor. In a series of 11 patients treated with ETV before liver transplant for acute flares of CHB with decompensation, none had evidence of lactic acidosis [223]. This highlights the importance of appropriate dose adjustment of NAs according to the calculated CrCl. Lactic acidosis is rarely reported among Asian patients with decompensated cirrhosis [164]. Although it is likely to be a rare event, clinical vigilance must be adopted for this potentially fatal complication, especially for those who are receiving combination therapy, and for those with impaired liver function and multi-organ failure.

ETV has a high genetic barrier to resistance. Drug resistance requires at least three codon substitutions, including rtL180M, rtM204I/V, plus a substitution at one of the following amino acids: rtT184S/G, rtS202I/G and/or rtM250V [179]. Among treatment-naïve patients, ETV resistance is very rare. In the long-term follow-up of the international trial on HBeAg-positive and HBeAg-negative patients and in a long-term follow-up study in Hong Kong, the cumulative probability of ETV resistance was 1.2 % after 5 years of ETV treatment [218].

ETV is effective in the treatment of ADV resistance [179]. Switching to ETV monotherapy (1 mg daily) in LAM resistant patients is associated with a >50 % cumulative risk of ETV, as rtM204I/V and rtL180M reduce the genetic barrier of resistance to ETV [224]. Among lamivudine resistant patients who had HBV DNA >2000 IU/ml on LAM and ADV combination therapy, a combination of entecavir 1 mg daily and ADV could achieve undetectable HBV DNA (<60 IU/ml) in 29 % in 1 year and 42 % in 2 years [225].

###### *Other direct antiviral agents*

Clevudine is an l-nucleoside pyrimidine analogue with potent antiviral activity against HBV. With clevudine 30 mg daily, the cumulative rate of undetectable HBV DNA is 67–83 % and HBeAg seroconversion is 23–31 % after 2–3 years [226, 227]. Virological breakthrough occurs in approximately 25 % of patients, and is primarily related to rtM204I ± rtL180M mutants. Myopathy was reported in up to 13 % of patients after being treated for a mean of 14 (range 9.3–23.5) months, but it was resolved spontaneously after stopping clevudine [226]. The global development of clevudine was terminated in 2009 because of case reports of serious myopathy related to myonecrosis.

Besifovir (LB80380) is an acyclic nucleotide phosphonate with chemistry similar to ADV and TDF. In a phase IIb, open-label, multicenter study among 114 treatment-naïve patients randomized to besifovir 90 mg daily, besifovir 150 mg daily and entecavir 0.5 mg daily for 48 weeks, undetectable HBV DNA was found in 63.6, 62.9 and 58.3 %, and HBeAg seroconversion was found in 11.1, 15 and 9.5 %, respectively [228]. No drug resistance or elevated serum creatinine was found among patients on besifovir. Ninety-four percent of patients on besifovir had reduced serum l-carnitine, but the l-carnitine levels returned to normal with supplement.

*Tenofovir alafenamide fumarate (TAF)* is a nucleotide reverse transcriptase inhibitor and a novel prodrug of tenofovir. Closely related to the commonly used reverse-transcriptase inhibitor tenofovir disoproxil fumarate, it has greater plasma stability than tenofovir disoproxil fumarate, and provides efficient delivery of active drug to hepatocytes at reduced systemic tenofovir exposures. In a recent study, noncirrhotic, treatment-naïve subjects with CHB were randomized (1:1:1:1:1) to receive tenofovir alafenamide 8, 25, 40, or 120 mg, or tenofovir disoproxil fumarate 300 mg for 28 days and were assessed for safety, antiviral response, and pharmacokinetics, followed up by off-treatment for 4 weeks. Tenofovir alafenamide was safe and well tolerated; declines in HBV DNA were similar to tenofovir disoproxil fumarate at all doses evaluated. Tenofovir alafenamide 25 mg has been selected for further hepatitis B clinical development [229].

###### *Combination of NAs*

De novo combination of lamivudine and adefovir does not improve viral suppression over lamivudine alone, although this reduces, but does not abolish, lamivudine resistance. Furthermore, adefovir resistance was not reported in this study. Combining telbivudine and lamivudine does not achieve greater reduction in HBV DNA than telbivudine monotherapy, but may even increase the risk of telbivudine resistance [230]. This suggests that NAs with the same resistance pattern should not be combined.

In one meta-analysis evaluating the effectiveness and resistance of de novo combination of lamivudine and adefovir dipivoxil compared with entecavir monotherapy for nucleos(t)ide-naive patients with chronic HBV infection (five studies, 328 patients), it was found that at 48 weeks, the combination group had superior virological response rates compared to the ETV group (90.0 vs. 78.9 %, *p* = 0.01). At week 96, LAM  +  ADV was more effective than ETV in ALT normalization [RR 1.11, 95 % CI (1.02, 1.21), *p* = 0.01] and HBeAg seroconversion [RR 2.00, 95 % CI (1.26, 3.18, *p* = 0.003)], and no significant difference was found in the virological response (*p* = 0.23). No viral resistance occurred in combination therapy and six patients in the ETV group were experienced with viral breakthrough [231]. In a recent clinical trial, 379 treatment-naïve patients were randomized to receive entecavir monotherapy (*n* = 186) or entecavir plus tenofovir (*n* = 198) [232]. By week 96, 76 % in the monotherapy arm and 83 % in the combination arm had HBV DNA below 50 IU/ml (*p* = 0.088). In a post hoc subgroup analysis, combination therapy was superior to entecavir monotherapy in patients with positive HBeAg and baseline HBV DNA over 8 log IU/ml. However, because the subgroup analysis was not planned a priori, the findings can only be considered exploratory and have to be confirmed in another study focusing on patients with high viral load. The efficacy of tenofovir monotherapy and higher dose entecavir (1.0 mg) has to be evaluated before combination therapy can be recommended for this group of patients.

#### Monitoring treatment and guidance for stopping therapy in chronic HBV-infected patients treated with nucleos(t)ide analogues

Efficacy and safety of NA therapy should be monitored regularly. Primary non-response, defined as <1 log_10_ IU/ml decline in HBV DNA level from baseline at month 3 of therapy, is rare with NA therapy [233]. Checking patient’s compliance is recommended in patients with primary non-response. Virological response at 6 months of lamivudine or telbivudine therapy and at 12 months of adefovir therapy is associated with the risk of emergence of drug resistance and virological and serological response with long-term therapy [234, 235]. HBV DNA level should be measured at month 3 and 6 of therapy and then every 3–6 months if agents with low genetic barrier are used (lam, Adefo, telbivudine), and every 6 months in patients treated with a high genetic barrier to resistance, such as entecavir or tenofovir. Serum ALT and HBeAg and anti-HBe (in patients with HBeAg-positive CHB) should be monitored every 3 months.

Checking compliance and testing for genotypic resistance should be done in patients with virological breakthrough during NA therapy. Due to potential nephrotoxicity, monitoring serum creatinine and serum phosphate levels should be done every 3 months during adefovir or tenofovir therapy [236, 237]. Muscle symptoms or muscle weakness should be monitored during telbivudine or clevudine therapy [180, 238]. A decline of HBsAg level during therapy may predict HBeAg or HBsAg loss with long-term telbivudine, entecavir or tenofovir therapy [239–241]. However, more data is needed to confirm the results before making a recommendation.

In HBeAg-positive CHB patients who achieve HBeAg seroconversion with undetectable HBV DNA, the relapse rates depend on the duration of consolidation therapy [242]. One recent study described 94 patients who stopped NA after at least 1 year of therapy. Patients could be HBeAg-positive or HBeAg-negative at the start of therapy, but all were HBeAg-negative and had undetectable HBV DNA (<200 IU/ml) at the time of discontinuation. Consolidation therapy was defined as treatment duration between the first undetectable HBV DNA (in case of HBeAg-positive patients after HBeAg loss) and NA discontinuation. Relapse was defined as HBV DNA >2000 IU/ml measured twice 6 months apart within 1 year, or retreatment after an initial HBV DNA elevation. At the start of therapy, 35 patients were HBeAg-positive and 59 were HBeAg-negative. The cumulative relapse rate was 33 % at 6 months, 42.7 % at 1 year, and 64.4 % at 5 years. Patients with at least 3 years of consolidation therapy (*n* = 37) had a 1-year relapse rate of 23.2 % compared to 57.2 % for 1–3 years of consolidation therapy (*n* = 32), and 55.5 % for <1 year of consolidation therapy (*n* = 20) (*p* = 0.002). For each additional year of consolidation therapy, patients were 1.3-fold more likely to lose HBsAg (hazard ratio 1.34; 95 % CI 1.02–1.75). Consolidation therapy of at least 3 years decreased the rate of relapse and increased the rate of HBsAg loss significantly [243].

Due to the high relapse rate after NA treatment discontinuation in patients with HBeAg-negative chronic hepatitis, treatment until HBsAg loss is generally recommended [218]. HBsAg levels may be a potential marker to guide treatment cessation. HBsAg levels of <2 log_10_ IU/ml at the end of treatment are associated with a lower relapse rate at 1–2 years post-treatment discontinuation (15 vs. 85 % in those with HBsAg level >2 log_10_ IU/ml at end of treatment) [244]. In one recent study to assess the outcome of patients withdrawing from NA therapy after HBsAg clearance, 27 (5 %) out of 520 CHB patients who received NA for prolonged periods ultimately lost serum HBsAg and were followed for 44 (12–117) months thereafter. It was concluded that patients reaching the therapeutic endpoint of HBsAg clearance can be safely withdrawn from NA following either anti-HBs seroconversion or at least 12 months of a post-clearance consolidation period [245]. However, in one recent meta-analysis including 22 studies with a total of 1732 HBeAg-negative patients (median duration of therapy, consolidation therapy and off-therapy follow-up ranged from 6 months to 8 years, 4–96 weeks and 6–80 months, respectively, and patients were monitored with serum ALT and HBV DNA monthly in the first 1–3 months and every 3–6 months thereafter in most studies), the 1-year off-therapy ‘virological relapse’ (HBV DNA >2000 IU/ml)and ‘clinical relapse’ (HBV DNA >2000 IU/ml + ALT elevation) occurred in <70 % and <50 % of the patients, respectively, and <40 % of the patients received re-treatment. These rates were higher in patients with shorter treatment, shorter consolidation therapy (<2 years) and those treated with less potent nucleos(t)ide analogues. Off-therapy severe flares were rare and hepatic decompensation was reported in only one patient with cirrhosis. Biochemical relapse reflecting enhanced immune-mediated hepatocyte killing may lead to a higher chance for off-therapy HBsAg seroclearance and possibly be desirable. Thus, with an appropriate stopping rule and a proper off-therapy monitoring plan, cessation of long-term nucleos(t)ide analogue therapy prior to HBsAg seroclearance in HBeAg-negative CHB is a feasible alternative to indefinite treatment [246].

Hepatitis relapse with hepatic decompensation and death is an important issue after cessation of NAs therapy in cirrhotic patients. The advantages of stopping NA therapy are a finite duration of treatment, with improved adherence and retention in care, reduced costs, and minimization of renal and bone toxicity. The disadvantages are the risk of reactivation of suppressed disease with discontinuation of therapy, resulting in an unpredictable worsening of disease and possible development of fulminant hepatitis and acute-on-chronic liver failure, as well as the risk of developing resistance with “stop–start” therapy. Cirrhotics have much less hepatic reserve for life-threatening hepatic decompensation after an exacerbation. However, one recent meta-analysis suggested that NAs withdrawel is safe even in cirrhotics, that off-therapy severe flares were rare and that hepatic decompensation was rarely observed in patients with cirrhosis [246].

3.6.1 Recommendations (results of currently available therapies, predictors of response to therapy, follow-up and stopping rules during NA therapy in patients with chronic HBV infection)
  3.6.1.1 Treatment-naïve patients can be treated with TDF 300 mg daily (A1), ETV 0.5 mg daily (A1), ADV 10 mg daily (A2), LdT 600 mg daily (A2) or LAM 100 mg daily (A2).
  3.6.1.2 TDF or ETV are the preferred NAs and should be used as first-line therapy (A1).
  3.6.1.3 During NA therapy, HBeAg, anti-HBe (in patients with HBeAg-positive) and ALT should be monitored every 3 months (A1).
  3.6.1.4 The HBV DNA level should be measured at month 3 and 6 of therapy and then every 3–6 months if agents with a low genetic barrier are used (lamivudine, adefovir, telbivudine), and every 6 months in patients treated with a high genetic barrier to resistance, such as entecavir or tenofovir (A1).
  3.6.1.5 Renal function and bone profile should be monitored at least every 3 months if TDF or ADV is used (A1).
  3.6.1.6 Muscle symptoms and muscle weakness should be monitored during telbivudine or clevudine therapy (A1).
  3.6.1.7 For HBeAg-positive patients without liver cirrhosis, the optimal duration of NA therapy is unknown, and the therapy can be stopped after at least 1 year (A1), but preferably after 3 years (C1) of additional therapy after HBeAg seroconversion with undetectable HBV DNA by PCR and persistently normal ALT levels.
  3.6.1.8 The optimal duration of NA therapy is unknown in patients with HBeAg-negative CHB. In patients without liver cirrhosis, the treatment can be withdrawn (1) after HBsAg loss following either anti-HBs seroconversion or at least 12 months of a post-HBsAg clearance consolidation period (B1), or (2) after treatment for at least 2 years with undetectable HBV DNA documented on three separate occasions, 6 months apart (B1).
  3.6.1.9 After stopping of NAs, patients should be monitored monthly for the initial 3 months and then every 3–6 months thereafter for relapse (A2).
  3.6.1.10 The stopping of NA therapy may also be considered in cirrhotic patients with a careful off-therapy monitoring plan (A1).

### 3.6.2 Results of and predictors of response to therapy with interferons

Currently, conventional interferon-alfa (IFN), lamivudine, adefovir, entecavir, telbivudine, tenofovir and pegylated interferonα2a (Peg-IFN-2a) have been approved for the treatment of CHB globally. Table 1 shows the comparison between these two treatment strategies (immune control vs. viral control). Peg-IFN-2b has been approved for the treatment of chronic HBV infection in a few countries. Thymosin α_1_ has also been licensed in some Asian countries. However, clevudine was only approved in Korea and the Philippines.

Immunomodulatory agents include conventional interferon-α (IFN), pegylated interferon (Peg-IFN), and thymosin α_1_. These agents have dual actions: enhancing host immunity to mount a defense against HBV and modest antiviral action. Over the past two decades, IFN-based therapy has been the mainstay of CHB treatment worldwide.

#### Conventional interferon

##### HBeAg-positive chronic hepatitis B

Meta-analyses of controlled trials in HBeAg-positive patients showed that treatment with conventional interferon-alfa (IFN) at a dose of 5 MU daily or 10 MU three times weekly for 4–6 months achieved higher HBeAg loss (33 vs. 12 %), HBV DNA suppression (37 vs. 17 %) and ALT normalization than untreated controls with a risk difference of around 25 % for each parameter. The rate of HBsAg seroclearance was also higher (7.8 vs. 1.8 %) in IFN-treated patients, with a risk difference of 5.6 %. Asian patients with elevated baseline ALT have IFN response rates comparable to Western patients. The efficacy of IFN treatment in children with elevated ALT was also similar to that in adults. Re-treatment of patients who failed previous IFN therapy could achieve HBeAg loss in 20–40 % of cases. A study of tailored regimen of IFN in 247 HBeAg-positive patients showed a higher sustained response than fixed 6-month treatment (40.5 vs. 28.3 %, *p* = 0.013). HBeAg seroconversion is durable in over 90 % and delayed HBeAg seroconversion could occur in 10–15 % at 1–2 years post-therapy, and there was up to a 15-year cumulative incidence of 75 % HBeAg seroconversion (vs. 52 % in control). In addition, IFN-treated patients have a lower likelihood of cirrhosis and HCC development, as well as better overall survival, especially among responders [19].

##### HBeAg-negative chronic hepatitis B

A 12-month IFN therapy showed the end-of-treatment biochemical and virological response rates in 60–90 %; however, sustained response rate was only 22 %. Extending IFN treatment for 24 months in Italian patients induced sustained response in 30 % and HBsAg clearance in 18 % at 6 years post-therapy. IFN treatment improved long-term outcomes, including reduction of HCC and survival and hepatic complication-free survival in patients with sustained response [19].

##### Compensated cirrhosis

Previous studies showed that compensated cirrhosis patients treated with IFN had comparable or even better response and a similar side effect profile as those without cirrhosis, with reduced risk of hepatic decompensation, HCC and prolonged survival in responders. However, IFN is contraindicated in patients with overt decompensated cirrhosis because it can precipitate hepatic decompensation, resulting in fatal complications [19]. Long-term follow-up studies showed that IFN treatment increased HBsAg seroclearance over time in patients with HBeAg loss. Two meta-analysis studies have confirmed these long-term benefits of IFN treatment in reducing liver disease progression to cirrhosis and HCC [247].

##### Pegylated interferon alfa alone

Pegylation of interferon-α (Peg-IFN) improves its pharmacokinetic and prolongs its half-life, which allows weekly injection. Two types of Peg-IFN have been developed: Peg-IFN α-2a (40 KD) and Peg-IFN α-2b (12 KD). In an early phase 2 study on Asian HBeAg-positive patients, the combined sustained viral response (SVR) (HBeAg loss, HBV DNA suppression, and ALT normalization) of Peg-IFN α-2a was twice that with conventional IFN α-2a (24 vs. 12 %; *p* = 0.036) at 24 weeks post-therapy [248]. A previous study of 24-week Peg-IFN α-2b in Chinese HBeAg-positive patients also confirmed a higher HBeAg loss rate than conventional IFN α-2b [249].

##### HBeAg-positive chronic hepatitis B

Two large phase 3 trials on HBeAg-positive patients showed that 1 year of Peg-IFN α-2a and Peg-IFN α-2b monotherapy resulted in HBeAg seroconversion in 32 % and 29 % of patients at 6 months post-therapy, respectively. The virological response based on HBV DNA suppression was found to be modest with Peg-IFN. HBV DNA suppression to <400 copies/ml was only obtained in 14 % of patients with Peg-IFN α-2a and 7 % with Peg-IFN α-2b, respectively. However, HBsAg seroconversion was achieved in 3–5 % of patients at 6 months post-therapy [250, 251]. In an analysis of the long-term effects of Peg-IFN, 83 % of 150 Asian HBeAg-positive patients treated with Peg-IFN α-2a for 48 weeks who achieved HBeAg seroconversion at 6 months post-therapy had sustained seroconversion at 12 months. Furthermore, 38 % of the patients who achieved HBeAg seroconversion at 12 months post-therapy had serum HBV DNA levels <400 copies/ml [252]. Moreover, long-term (mean follow-up of 3 years) sustained negativity of HBeAg and HBsAg in 172 European HBeAg-positive patients treated with Peg-IFN α-2b was 37 and 11 %, respectively. In particular, sustained negativity of HBeAg and HBsAg was observed in 81 and 30 % of 64 patients with an initial serological response (HBeAg negativity at 26 weeks post-therapy) [253]. Of note, most of the patients who cleared HBsAg were infected by HBV genotype A.

A recent prospective study with mostly Asian patients compared the treatment response of different doses and durations of Peg-IFN α-2a in HBeAg-positive patients [254]. The data showed that 180 μg/week of Peg-IFN α-2a for 48 weeks was superior to regimens with shorter duration or lower dose. Therefore, the currently recommended dose and duration of Peg-IFN α-2a therapy is 180 μg/week for 48 weeks. The recommended dose of Peg-IFN α-2b therapy is 1.5 μg/kg/week for 48 weeks.

##### HBeAg-negative chronic hepatitis B

With 1 year of Peg-IFN α-2a therapy, the data revealed that HBV DNA <4000 IU/ml occurred in 43 % of patients and HBsAg loss was reported in 4 % at 6 months post-therapy [255]. After 3 years of follow-up, 28 % of HBeAg-negative patients had HBV DNA <2000 IU/ml, and HBsAg clearance rate increased to 8.7 % [256]. In addition, the two studies using Peg-IFN α-2a therapy also found that Peg-IFN–based therapy was superior to lamivudine alone in inducing HBeAg seroconversion in HBeAg-positive patients and in suppressing viral replication in HBeAg-negative patients. All three studies showed that the therapeutic efficacy was comparable between Peg-IFN monotherapy and combination therapy of Peg-IFN plus lamivudine. A recent study on 120 Caucasian HBeAg-negative patients with genotype D infection explored whether longer treatment duration could lead to a better response, and the results showed that extending treatment duration to 96 weeks increased response rate (HBV DNA level <2000 IU/ml at 1 year post-therapy) from 11.8 to 28.8 % [257].

##### Chronic hepatitis B with cirrhosis

A prior study on 24 HBeAg-positive patients with well-compensated cirrhosis treated with 52 weeks of Peg-IFN α-2b with or without lamivudine showed a higher rate of HBeAg serconversion and HBV DNA <10,000 copies/ml at 26 weeks post-therapy than those without cirrhosis (30 vs. 14 %, *p* = 0.02) [258]. In addition, improvement of liver fibrosis was found more frequently in patients with advanced fibrosis than in those without (66 vs. 22 %, *p* < 0.001). The side effects were comparable between patients with and without advanced fibrosis.

#### Combination therapy of IFN and NAs

With current antiviral agents, most CHB patients fail to obtain HBsAg seroclearance, which is the ultimate goal of HBV therapy. Furthermore, relapse is common during post NA therapy follow-up. Therefore, combination therapy could be considered the ideal treatment for CHB. There are three approaches for administering combination therapy: NA followed by addition of Peg-IFN and continuation of NA; starting with Peg-IFN followed by addition of NA; or simultaneous administration of NA and Peg-IFN. There is lack of data to recommend one over the other. However, most investigators have used the first approach and scientifically prefer the basis of viral load reduction followed by immune modulation as a logical step. The three approaches have been used with different NAs and Peg-IFN with improved results compared to monotherapy with either group of drugs.

##### Combination of Peg-IFN with lamivudine

However, in both HBeAg-positive and HBeAg-negative subjects, simultaneous commencement of Peg-IFN and LAM tends to provide a more profound treatment effect on viral suppression without superior sustained virological off-treatment response, compared with Peg-IFN monotherapy [250, 251, 255].

A study on 36 treatment-naive HBeAg-positive patients who received LAM 100 mg per day for 4 weeks before adding Peg-IFN for the following 24 weeks showed that they achieved higher sustained (6 months after end of treatment) virological responses compared with the 27 patients who received Peg-IFN from the start (undetectable HBV DNA and HBeAg losses 50 vs. 15 %; *p* = 0.028; 39 vs. 15 %; *p* = 0.05, respectively) [259]. However, another study found no difference in efficacy between32-week Peg-IFN started simultaneously with LAM and that started 8 weeks before LAM or 8 weeks after commencement of LAM, 24 weeks after the end of therapy. All patients received lamivudine until week 104 [260].

##### Combination of Peg-IFN with adefovir

In a multicenter prospective study, 160 HBeAg-positive patients were randomized to Peg-IFNa-2a monotherapy or to individualized combination therapy with Peg-IFNa-2a + adefovir dipivoxil (ADV) based on the baseline features and treatment response. At week 96, combined response (ALT normalization and undetectable HBV DNA), HBeAg clearance, and seroconversion rates were higher in those patients treated with the combination than in those treated with Peg-IFNa-2a alone [261]. An Italian multicenter study in 60 HBeAg-negative patients showed a similar sustained virological response (i.e., HBV DNA <2000 IU/ml 24 weeks) after the EOT among those treated with a 48-week combination of Peg-IFNa-2a + ADV or Peg-IFNa-2a alone (23 vs. 20 %, *p* = 0.75), with only one patient (3 %) in the combination group achieving HBsAg loss [262].

##### Combination of Peg-IFN with telbivudine

A study in 159 HBeAg-positive patients reported that a combination of Peg-IFNa-2a and telbivudine (LdT) led to a higher rate of undetectable HBV viral load and greater reductions in HBeAg and HBsAg levels than either drug alone [263]. Another study compared the efficacy and safety of two sequential regimens: Peg-IFN for 24 weeks followed by LdT for 24 weeks (Peg-IFN first), or vice versa (LdT first), in 30 HBeAg-negative patients. At the end of follow-up (week 72), more patients treated with LdT first had HBV DNA <2000 IU/ml (47 vs. 13 %, *p* = 0.046). Sequential treatment with Peg-IFN followed or preceded by 24 weeks of LdT was safe; only one patient dropped out because of myalgia [264]. However, presently the combinations of Peg-IFN with LdT should be avoided, as a high risk of severe polyneuropathy development was reported in those treated with the combination therapy, leading to an early discontinuation of one study [263].

##### Combination of Peg-IFN with entecavir

One recent study (the OSST study) reported on 100 Chinese HBeAg-positive patients with maintained virological response on ETV who switched to a finite course of Peg-IFN α-2a and achieved significantly higher rates of HBeAg seroconversion and HBsAg clearance than 100 patients who continued ETV [265].

Another global randomized trial (the ARES study) was conducted in European and Chinese HBeAg-positive patients. In this open-label, multicenter randomized trial, HBeAg-positive CHB patients with compensated liver disease started on ETV monotherapy (0.5 mg/day) and were randomized in a 1:1 ratio to either Peg-IFN add-on therapy (180 µg/week) from week 24 to 48 (*n* = 85), or to continue ETV monotherapy (*n* = 90). Response was defined as HBeAg loss with HBV DNA <200 IU/ml at week 48. Responders discontinued ETV at week 72. All patients were followed until week 96. Response was achieved in 16/85 (19 %) patients allocated to the add-on arm versus 9/90 (10 %) in the monotherapy arm (*p* = 0.095). Adjusted for HBV DNA levels prior to randomized therapy, the Peg-IFN add-on was significantly associated with response (OR 4.8, 95 % CI 1.6–14.0, *p* = 0.004). Eleven (13 %) of add-on treated patients achieved disease remission after ETV cessation, versus 2/90 (2 %) of patients treated with monotherapy (*p* = 0.007), which was 79 % (11/14) versus 25 % (2/8) of those who discontinued ETV (*p* = 0.014). At week 96, 22 (26 %) patients assigned add-on versus 12 (13 %) assigned monotherapy achieved HBeAg seroconversion (*p* = 0.036). Peg-IFN add-on led to significantly more decline in HBsAg, HBeAg and HBV DNA (all *p* < 0.001). Add-on therapy resulted in more viral decline and appeared to prevent relapse after stopping ETV. Hence Peg-IFN add-on therapy may facilitate the discontinuation of nucleos(t)ide analogues [266].

##### *Combination of Peg*-*IFN with tenofovir*

In one study on HBeAg-positive CHB, raised ALT (48–200 IU/ml) patients, all patients received tenofovir (300 mg/day for 12 weeks), followed by randomization to tenofovir plus peg-interferon a2b 1.5 mcg/kg/weekly for 24 weeks (sequential therapy; *n* = 30) or tenofovir monotherapy (*n* = 30). Daily tenofovir was continued thereafter until HBsAg loss. At 48 weeks, 60 % in the sequential therapy group and 30 % in tenofovir monotherapy had normal ALT (*p* = 0.02). Patients receiving sequential therapy had higher HBV DNA loss (80 vs. 53 %; *p* = 0.028), mean HBV DNA reduction [6.70 ± 1.64 vs. 4.43 ± 2.44 log10 (*p* < 0.001)], and HBeAg seroconversion (53.3 vs. 23.3 %; *p* = 0.017), compared to the tenofovir monotherapy group. Two patients on sequential therapy had HBsAg loss by 48 weeks compared with none in tenofovir monotherapy [267].

In a recent open-label study (Study 149), a total of 740 CHB patients (60 % positive for HBeAg) without advanced bridging fibrosis or cirrhosis were randomly assigned to receive tenofovir + pegylated interferon for 48 weeks, tenofovir + pegylated interferon for 16 weeks, continuing on Tenofovir alone through week 48, tenofovir monotherapy for 120 weeks (continuous monotherapy) or pegylated interferon monotherapy for 48 weeks. At the end of treatment, HBsAg levels declined most in the 48-week tenofovir plus pegylated interferon arm (−1.1 log_10_), followed by interferon monotherapy (−0.8.1 log_10_), the 16-week tenofovir combination regimen (−0.5 log_10_) and tenofovir monotherapy (−0.3 log_10_). At 48 weeks, 7.3 % of patients taking the 48-week tenofovir plus pegylated interferon regimen showed HBsAg loss. Response rates were substantially lower in the 16-week tenofovir combination arm and interferon monotherapy arm (both 2.8 %). None taking tenofovir alone experienced HBsAg loss. By 72 weeks, the rate of HBsAg loss rose to 9.0 % in the 48-week tenofovir plus pegylated interferon group, while remaining the same in the other three arms. A total of seven patients experienced HBsAg seroreversion, or reappearance after loss (four in the 48-week combination arm and three in the 16-week combination arm) [268].

Taken together, simultaneous combination of Peg-IFN plus tenofovir or sequential combination therapy using entecavir first followed by Peg-IFN shows promising results; however, future large studies are needed to confirm these results.

#### Peg-IFN add-on treatment in NAs responders

Because it has been observed that during effective NAs therapy, HBsAg decline is very slow and may require decades to achieve undetectable levels, an alternative use of Peg-IFN in chronic HBV-infected patients is to add on Peg-IFN to NAs responders to accelerate the HBsAg decline. One study reported HBsAg kinetics in 12 patients (nine HBeAg-negative) having undetectable HBV DNA (<116 copies/ml) for more than 6 months on NAs (LAM = 1, LAM + ADV = 2, ETV = 7, ETV + TDF = 2), and who additionally received Peg-IFN as an individualized therapy. After add-on of PegIFN, a rapid decline of HBsAg occurred in two patients, to HBsAg levels of 0.14 and 0.02 IU/ml at week 48, respectively (corresponding to a maximal reduction of 2.9 log10 and 4.25 log10). Three patients discontinued Peg-IFN early due to side effects, whereas seven patients withdrew from treatment after a mean of 16 weeks due to a suboptimal HBsAg response (decline of 0.09 log10 only) [269]. In one randomized controlled trial (PEGON study) conducted in Europe and China, 82 HBeAg-positive patients with compensated liver disease were treated for at least 12 months with entecavir (ETV) or tenofovir (TDF) with subsequent HBV DNA <2000 IU/ml at randomization. Patients were randomized to 48 weeks of Peg-IFN addition, or 48 weeks of continued NA monotherapy. Response (HBeAg seroconversion with HBV DNA <200 IU/ml) was assessed at week 48. Responders will discontinue treatment after 24 weeks consolidation treatment (week 72), with subsequent off-treatment follow-up until week 96. Week 48 results were presented at AASLD 2014. Ninety-six percent of patients were of Asian ethnicity, with an average age of 33 years. Response, as well as HBeAg seroconversion alone, was achieved in 17 % of patients who received Peg-IFN add-on compared to 5 % of patients who continued NA monotherapy (*p* = 0.15). HBeAg loss was achieved in 33 % of patients who received Peg-IFN add-on compared to 18 % in the NA monotherapy group (*p* = 0.14). Peg-IFN add-on resulted in significantly more HBsAg decline at week 48 (0.59 vs. 0.29 log IU/ml, *p* = 0.021). HBsAg decline >1 log IU/ml was achieved in 19 % of the Peg-IFN add-on group compared to 0 % in the NA monotherapy group (*p* = 0.005). One patient who received Peg-IFN add-on had clearance of HBsAg at week 48 [270]. Preliminary results of the multicenter, randomized controlled phase III trial ANRS-HB06 PEGAN study presented at AASLD 2014 suggested that addition of a 48-week course of Peg-IFN alfa-2a to oral anti-HBV therapy in HBeAg-negative CHB patients with undetectable serum HBV DNA for at least 1 year results in a low rate of HBsAg clearance (6.6 %), and that low baseline HBs Ag titers and a history of HBeAg seroconversion, either spontaneously or under HBV therapy, may increase HBsAg clearance rate [history of HBeAg seroconversion prior to randomization (23.5 vs. 3.3 %) (*p* = 0.0185)] [271].

#### Baseline and on-treatment predictors of response to Peg-IFN (Table 6)

##### Lower serum HBV DNA level and elevated ALT levels

In CHB patients receiving IFN or Peg-IFN treatment, lower HBV DNA level and higher ALT level are known as baseline predictors for a better response. For HBeAg-positive patients receiving Peg-IFN-based treatment, a pooled analysis showed that a lower level of HBV DNA (<9 log_10_ copies/ml) and an elevated ALT level (>2 times of upper limit of normal) were associated with a higher sustained response rate (HBeAg loss and HBV-DNA level <2000 IU/ml at 6 months post-therapy) [272]. For HBeAg-negative patients, a lower HBV DNA level and a higher ALT level were both associated with a higher treatment response to Peg-IFN-based therapy [256].

**Table 6:** Baseline predictors and stopping rules of 48-week pegylated interferon therapy in Asian and Caucasian chronic hepatitis B patients

	HBeAg-positive		HBeAg-negative	
	Asian	Caucasian	Asian	Caucasian
Lower HBV DNA level	Better response	Better response	Not clear	Not clear
Higher ALT level	Better response	Better response	Not clear	Not clear
Genotype	B and C are comparable	A is better than D	B and C are comparable	Not clear
Precore stop codon (PC) and basal core promoter (BCP) mutants	Mutant better than wild type	Wild type better than mutant	Not clear	Not clear
IL28b SNP	No predictive value	Controversial	Not clear	Controversial
Lower level HBeAg	Better response	Better response	Not applied	Not applied
Stopping rule at 12-weeks	No decline of HBsAg level at week 12	HBsAg level >20,000 IU/ml at week 12	No rule could achieve 95 % of negative predictive value	Only in genotype D patients: without HBsAg decline and with <2log HBV DNA decline

##### HBV genotype

In a pooled analysis on two large clinical trials with HBeAg-positive patients who received 12-month Peg-IFN-based therapy, Buster et al. [272] found that patients with genotype A infection had the best response, followed by genotypes B and C, which had similar responses, while those with genotype D had the worst response. These results lend support to the recommendation that Peg-IFN therapy is suitable for patients with genotype A rather than genotype D infection. In patients with genotype B or C infection, Asian studies reported that in a shorter 6-month Peg-IFN treatment, response was better in genotype B infection compared to genotype C infection [273, 274]. However, the HBeAg seroconversion rate is similar between genotypes B and C after 12-month Peg-IFN-based treatment, which is the current standard of care. When HBsAg clearance is defined as treatment endpoint in HBeAg-positive patients, subgroup analysis from the clinical trial using Peg-IFN α-2b showed that genotype A had the highest rate of HBsAg loss compared to other genotypes [272]. For HBeAg-negative patients, the data comparing the sustained response among patients receiving Peg-IFN α-2a ± lamivudine showed that there was no difference between genotypes A and D or genotypes B and C after a long-term follow-up of 3 years [256].

Taking these lines of evidence together, it is concluded that with a standard 12-month Peg-IFN treatment, HBeAg-positive patients infected with genotype A have the best response, followed by genotypes B and C, who have a similar response, while those infected with genotype D have the lowest response. For HBeAg-negative patients, the role of HBV genotype may be minimal.

##### HBeAg level, precore and basal core promoter mutants

A retrospective analysis on 271 HBeAg-positive patients who received 48-week Peg-IFN α-2a ± lamivudine showed that HBeAg seroconverters have a lower baseline and on-treatment levels of HBeAg [275]. However, thus far, there is no commercial assay available for measuring HBeAg concentrations in clinical practice. Two Asian studies indicated that pre-therapy BCP mutations could increase HBeAg clearance rate in patients receiving Peg-IFN treatment [273, 276]. These results highlight that further studies are needed to confirm the predictive value of HBeAg-associated factors in HBeAg-positive patients with Peg-IFN therapy. A recent study quantified the proportion of precore (PC) and BCP mutants at baseline and during IFN or Peg-IFN treatment in 203 HBeAg-positive patients, and found a dose response relationship between the proportion of PC/BCP mutants and HBeAg seroconversion rate [277]. These data suggested that both PC and BCP mutants were qualitatively and quantitatively associated with a higher response rate to IFN or Peg-IFN therapy in Asian HBeAg-positive patients. However, a European study with 214 HBeAg-positive patients receiving Peg-IFN α-2b ± lamivudine showed that the presence of either PC or BCP mutants lowered the rate of sustained response (wild-type vs. presence of mutant: 34 vs. 11 %) [278]. Taken together, PC and BCP mutant may play different roles in Asians and Caucasians, which may be attributable to different HBV genotypes.

##### Quantitative serum HBsAg level

Since serum HBsAg level varies depending on the balance between viral replication and host immunity, it is hypothesized that HBsAg may serve as a biomarker to predict treatment response to Peg-IFN. A French study first reported that a decline in serum HBsAg level of 0.5 log_10_ IU/ml at week 12 could differentiate sustained responders from relapser in HBeAg-negative patients [279]. From then on, several retrospective studies proposed the role of HBsAg level as a “stopping rule” at week 12 of Peg-IFN treatment in both HBeAg-positive and HBeAg-negative patients. However, further prospective studies are still required to validate these findings.

In a study enrolling 202 HBeAg-positive Caucasian patients with genotype A or D infection [280], only 3 % of patients without decline of HBsAg level at week 12 could achieve sustained response [negative predictive value (NPV) of 97 %]. However, this was not validated well in another study with 399 HBeAg-positive Asian patients with genotype B or C infection (NPV of 82 %) [281]. Instead, the Asian study proposed an alternate stopping rule, HBsAg >20,000 IU/ml at week 12. To investigate which stopping rule was more universally applicable across HBV genotypes, data from three large-scale clinical trials were pooled, and it was concluded that if treatment response was defined as sustained response, then the 12-week stopping rule can be defined as no decline of HBsAg level for genotype A and D, but HBsAg level >20,000 IU/ml for genotype B and C patients; while HBsAg >20,000 IU/ml at 24 week could be applied to all patients as the 24 week stopping rule, irrespective of HBV genotype [282].

Most data regarding HBeAg-negative patients included genotype D infection. When using HBV DNA level <2000 IU/ml combined with normal ALT level at 6 months post-therapy as the treatment endpoint, the stopping rule of no HBsAg decline plus <2 log HBV DNA decline at week 12 had NPV of 100 % [283]. For patients with non-genotype D infections, HBsAg decline of 10 % has been shown to predict treatment response at 1-year post-therapy (47.2 and 16.4 % for HBsAg decline ≥10 vs. <10 %, respectively) [284]. In summary, a stopping rule for Peg-IFN therapy at week 12 or 24 is clinically useful in HBeAg-positive patients. For HBeAg-negative patients with genotype D infection, a week 12 stopping rule is also clinically applicable. However, for HBeAg-negative patients with non-genotype D infection, more studies are warranted.

##### Quantitative serum anti-HBc level

Quantitative serum anti-HBc level has been reported to reflect host immune status and hepatitis activity. However, its clinical significance in CHB therapy remains limited. In a retrospective cohort study consisting of 231 and 560 patients enrolled in two phase IV, multicenter, randomized, controlled trials treated with Peg-IFN or NA-based therapy, the role of quantitative serum anti-HBc level in predicting HBeAg seroconversion was evaluated. The data showed that at the end of trials, 99 (42.9 %) and 137 (24.5 %) patients achieved HBeAg seroconversion in the Peg-IFN and NA cohorts, respectively. Baseline anti-HBc level of 4.4 log_10_ IU/ml was the optimal cutoff value to predict HBeAg seroconversion for both Peg-IFN and NA. Patients with baseline anti-HBc ≥4.4 log_10_ IU/ml and baseline HBV DNA <9 log_10_ copies/ml had 65.8 % (50/76) and 37.1 % (52/140) rates of HBeAg seroconversion in the Peg-IFN and NA cohorts, respectively. In pooled analysis, other than treatment strategy, the baseline anti-HBc level was the best independent predictor for HBeAg seroconversion (OR 2.178; 95 % CI 1.577–3.009; *p* < 0.001). Therefore, baseline anti-HBc titer may serve as a useful predictor of Peg-IFN and NA therapy efficacy in HBeAg-positive CHB patients, which could be used for optimizing the antiviral therapy of CHB [285].

##### Quantitative hepatic HBsAg level

In addition to serum HBsAg level, the relationship between hepatic HBsAg level and treatment response of IFN-based therapy has been explored in 45 HBeAg-positive patients, and there was a positive correlation between baseline serum HBsAg level and hepatic HBsAg level [286].

##### Interleukin-28B genotype

Several interleukin-28B (*IL28B*)-associated single nucleotide polymorphisms (SNPs), including CC genotype of rs12979860 and TT genotype of rs8099917, are associated with a higher response rate in Peg-IFN-based treatment for chronic hepatitis C. Whether the *IL*-*28B* SNPs could also predict Peg-IFN-based treatment response in CHB has been actively investigated. Nevertheless, the results remain controversial. The first study enrolled 115 patients receiving 6-month Peg-IFN treatment, and there was no correlation noted between *IL28B* SNPs and treatment response [273]. In contrast, a multicenter study, which enrolled 205 HBeAg-positive patients receiving Peg-IFN ± lamivudine from 11 European and Asian centers, yielded contradictory results [287]. In this study, around 65 % of the patients were of Asian descendants. They found that the CC genotype of rs12979860 was highly associated with HBeAg seroconversion. However, this is the only study showing a positive correlation in HBeAg-positive patients. Most of the subsequent studies failed to confirm these findings [288]. With regard to HBeAg-negative patients, only one retrospective study has been reported. The authors included 101 Caucasian patients receiving IFN or Peg-IFN for 24 months and were followed for a median of 11 years [289]. They found that the CC genotype of rs12979860 was associated with higher rates of SVR (HBV DNA level <2000 IU/ml) and HBsAg clearance. In summary, most studies involving Asian patients failed to identify *IL28B* genotype as a possible predictor for HBV treatment response. In Caucasian patients, further investigations are needed.

##### SNPs near HLA-DP region

Two SNPs near *HLA*-*DP* regions rs3077 and rs9277535 were shown to play a role in spontaneous HBsAg clearance in patients with chronic HBV infection. Since spontaneous clearance of HBsAg is a result of host immune activity, which could be enhanced by Peg-IFN treatment, it seems reasonable to investigate the association between the *HLA*-*DP* SNPs and the treatment response to Peg-IFN. In fact, it has been shown that rs3077 GG genotype was associated with a better treatment response in HBeAg-positive patients receiving Peg-IFN therapy in Asian studies [290]. Although both were retrospective and small-scale studies, these encouraging data still suggested that the role of *HLA*-*DP* SNPs in Peg-IFN therapy are worthy of further studies.

#### Side effects of IFN-based therapy

The most frequently reported side effects of IFN-based therapy are flu-like symptoms, headache, fatigue, myalgia, alopecia, and local reaction at the injection site. IFN and Peg-IFN have myelosuppressive effects; however, neutropenia <1000/mm^3^ and thrombocytopenia <500,000/mm^3^ are not common unless patients already have cirrhosis or low cell counts prior to IFN-based treatment. Neutropenia and thrombocytopenia induced by IFN or Peg-IFN do not significantly increase the risk of infection and bleeding, except in patients with cirrhosis or immunosuppression. Although IFN and Peg-IFN have many side effects, they are well tolerated. Premature discontinuation due to patient’s intolerability has been reported in 2–8 % of patients treated with Peg-IFN.

#### Therapy with pegylated interferon: overall conclusions

Currently, monotherapy with a potent NA or Peg-IFN is recommended as the first-line therapy. However, Peg-IFN is not recommended for patients who have hepatic decompensation, immunosuppression or medical or psychiatric contraindications. Peg-IFN is more appropriate for young patients, those who can better tolerate side effects and those who are reluctant to receive indefinite treatment. During treatment, Peg-IFN could be stopped at week 12 or 24 if the patients are found to be primary non-responders, which is defined by the genotype-specific HBsAg stopping rule. Finally, useful and reliable viral and host factors predictive of treatment outcomes need further exploration to guide individualized Peg-IFN therapy in the future.

### Monitoring treatment and guidance for stopping therapy in chronic HBV-infected patients treated with interferons

The currently recommended dose and duration of Peg-IFN α-2a therapy for both HBeA-positive and HBeA-negative CHB is 180 μg/week for 48 weeks. In patients receiving Peg-IFN therapy, full blood cell counts and serum ALT levels should be monitored monthly and thyroid function should be monitored every 3 months. All patients should be monitored for safety through 12 months of treatment.

In HBeAg-positive patients, HBeAg, anti-HBe antibodies and serum HBV DNA levels should be checked at 6 and 12 months of therapy and at 6 and 12 months post-therapy. Sustained HBeAg seroconversion together with ALT normalization and serum HBV DNA below 2000 IU/ml post-therapy is the desired therapeutic endpoint. HBeAg-positive patients who develop HBeAg seroconversion with Peg-IFN therapy require long-term follow-up because of the possibility of HBeAg seroreversion or progression to HBeAg-negative CHB. HBsAg should be checked at 12-month intervals after HBeAg seroconversion if HBV DNA is undetectable, as the rate of HBsAg loss increases over time. Patients who become HBsAg seroclearance should be tested for anti-HBs antibodies. Patients treated with Peg-IFN who achieve significant decline of HBV DNA and/or HBsAg levels through 3 or 6 months of therapy have an increased likelihood of treatment response. In contrast, HBeAg-positive patients treated with Peg-IFN who fail to achieve serum HBsAg levels below 20,000 IU/ml or any decline in serum HBsAg levels by month 3 have a low likelihood of HBeAg seroconversion [274]. Thus, cessation of Peg-IFN therapy may be considered.

In HBeAg-negative patients, serum HBV DNA levels should be checked at 6 and 12 months of therapy and at 6 and 12 months post-therapy. A sustained virological response with HBV DNA <2000 IU/ml post-therapy is generally associated with the remission of disease activity. HBsAg should be checked at 12-month intervals if HBV DNA remains undetectable during the follow-up. Patients who become HBsAg seroclearance should be tested for anti-HBs antibodies. HBeAg-negative patients who achieve sustained response at 12 months post-therapy still require long-term follow-up because of the risk of future disease reactivation. HBeAg-negative patients, especially those with genotype D infection, who fail to achieve any decline in serum HBsAg levels and a >2 log10 IU/ml decline in serum HBV DNA levels by month 3 of Peg-IFN therapy, have a very low likelihood of treatment response [274, 291, 292]. Thus, cessation of Peg-IFN therapy should be considered.

3.6.2 Recommendations: results of currently available therapies, predictors of response to therapy, follow-up and stopping rules during interferon therapy in chronic HBV infection
  3.6.2.1 Treatment-naïve patients can be treated with Peg-IFN-a2a 180 μg weekly or Peg-IFN-a2b 1–1.5 μg/kg weekly (A1).
  3.6.2.2 For Peg-IFN, the recommended duration is 48 weeks for both HBeAg-positive and–negative patients (A1).
  3.6.2.3 In patients treated with Peg-IFN, full blood counts and serum ALT levels should be monitored monthly and TSH should be monitored every 3 months. All patients should be monitored for safety through 12 months of treatment (A1).
  3.6.2.4 In regions endemic for HBV genotype A and D infection, HBV genotyping should be done among patients being considered for IFN therapy (A1).
  3.6.2.5 In HBeAg-positive patients, HBeAg and anti-HBe antibodies and serum HBV DNA levels should be checked at 6 and 12 months of treatment and at 6 and 12 months post-treatment (A1). HBsAg levels should be checked every 3 months (A1).
  3.6.2.6 For HBeAg-positive patients treated with Peg-IFN who fail to achieve serum HBsAg levels below 20,000 IU/ml (genotype B and C infection), or any decline in serum HBsAg levels (genotype A and D infection) by week 12 and serum HBsAg levels below 20,000 IU/ml by week 24 (genotype A–D infection), stopping Peg-IFN therapy should be considered (B2).
  3.6.2.7 In HBeAg-negative patients, serum HBV DNA levels should be measured at 6 and 12 months of treatment and at 6 and 12 months post-treatment (A1). HBsAg levels should be checked every 3 months (A1).
  3.6.2.8 For HBeAg-negative patients, especially those with genotype D infection, who fail to achieve any decline in serum HBsAg levels and a >2 log10 IU/ml decline in serum HBV DNA levels by month 3 of Peg-IFN therapy, discontinuation of Peg-IFN therapy should be considered (B2).

### 3.7 Treatment strategies for first-line therapy in pre-cirrhotic chronic hepatitis B: nucleos(t)ide analogues or interferons or a combination

The two therapeutic approaches available for the suppression of HBV replication include antiviral agents [nucleos(t)ide analogues, NAs] and immune-based therapies (IFN-α or pegylated-IFN-α) (Table 7).

**Table 7:** Comparison of two treatment strategies for chronic hepatitis B

	Pegylated interferon	Nucleos(t)ide analogues
Strategy	Sustained off-therapy response (immune control)	Maintained on-treatment response (viral control)
Goal	Low HBV DNA level (<2000 IU/ml) and normal ALT level	Undetectable HBV DNA level and normal ALT level
Duration	Finite	Prolonged or indefinite
Effectiveness	Sustained response in ~30 % of patients after 48 weeks of therapy	Successful suppression of viral replication with continued treatment, but high relapse rate after stopping the treatment
Contraindication	Hepatic decompensation, immunosuppression, pregnancy, psychiatric or medical contraindications	Nil

The main theoretical advantages of Peg-IFN are the absence of resistance and the potential for immune-mediated control of HBV infection with an opportunity to obtain a sustained virological response off-treatment, and a chance of HBsAg loss in patients who achieve and maintain undetectable HBV DNA, and thus potential of finite treatment duration. Peg-IFN-induced HBeAg seroconversion might be more durable than NA-induced HBeAg seroconversion. Frequent side effects and subcutaneous injection are the main disadvantages of (PEG-) IFN treatment. (PEG-) IFN is contraindicated in patients with decompensated HBV-related cirrhosis or autoimmune disease, in patients with uncontrolled severe depression or psychosis, and in female patients during pregnancy. Orally administered NAs are well tolerated, but the rate of viral relapse is common once the treatment is ceased, which necessitates long-term or even life-long treatment. Current data show that long-term ETV or TDF therapy is relatively safe and has minimal risk of drug resistance. Therefore, Peg-IFN should be highly considered in young people who are planning to have babies and patients with a high chance of achieving sustained off-therapy response, such as HBeAg-positive patients who have high pre-treatment ALT levels, genotype A infection or those with more favorable predictors.

*Finite*-*duration treatment with (PEG*-*) IFN* This strategy is intended to achieve a sustained off-treatment virological response. Peg-IFN, if available, has replaced standard IFN in the treatment of CHB, mostly due to its easier applicability (once weekly administration). A 48-week course of Peg-IFN is mainly recommended for HBeAg-positive patients with the best chance of HBeAg seroconversion. It can also be used for HBeAg-negative patients, as it is practically the only option that may offer a chance for sustained off-treatment response after a finite duration of therapy. Full information about the advantages, adverse events and inconveniences of Peg-IFN versus NAs (Table 7) should be provided so the patient can participate in the decision. Simultaneous combinations of Peg-IFN with NAs such as entecavir and tenofovir have been shown to be safe with promising results. Sequential combination therapies using viral load reduction followed by addition of Peg-IFN have been found to be safe with improved seroconversion rates compared to monotherapies. These approaches need to be confirmed in larger studies before they are recommended.

*Finite*-*duration treatment with a NA* This strategy can be is feasible for HBeAg-positive patients who seroconvert to anti-HBe on treatment. However, treatment duration is unpredictable prior to therapy, as it depends on the timing of HBeAg seroconversion and the treatment continuation post-HBeAg seroconversion. HBeAg seroconversion may not be durable after NAs discontinuation, at least with less potent agents, in a substantial proportion of these patients requiring close virological monitoring after treatment cessation.

An attempt for finite NA treatment should use the most potent agents with the highest barrier to resistance, to rapidly reduce levels of viremia to undetectable levels and avoid breakthroughs due to HBV resistance. Once HBeAg seroconversion occurs during NA administration, treatment should be prolonged for at least 1 year and preferably an additional 3 years to try to achieve a durable off-treatment response. Consolidation therapy of at least 3 years decreases the rate of relapse and increases the rate of HBsAg loss significantly [243].

*Long*-*term treatment with NA(s)* This strategy is necessary for patients who are not expected to or fail to achieve a sustained off-treatment virological response and require extended therapy, i.e., for HBeAg-positive patients who do not develop HBeAg seroconversion and HBeAg-negative patients. This strategy is also recommended in patients with cirrhosis, irrespective of HBeAg status or anti-HBe seroconversion on treatment. The most potent drugs with the optimal resistance profile, i.e., tenofovir or entecavir, should be used as first-line monotherapies.

There are as yet no data to indicate an advantage of de novo combination treatment with NAs in NA naive patients receiving either entecavir or tenofovir.

3.7 Recommendations: treatment strategies for first-line therapy in pre-cirrhotic CHB: nucleos(t)ide analogues or interferons or a combination
  3.7.1 A course of Peg-IFN may be the most appropriate first-line treatment strategy when the purpose of treatment is to achieve a sustained response after a defined treatment course compared with NAs requiring long-term administration (B2).
  3.7.2 A 48-week course of Peg-IFN is mainly recommended for HBeAg-positive patients with the best chance of HBeAg seroconversion (B1). It can also be used for HBeAg-negative patients, as it is practically the only option that may offer a chance for sustained off-treatment response after a finite duration of therapy (B2).
  3.7.3 Despite the tolerability and the higher rates of off-therapy response compared to NAs, the benefits of Peg-IFN are restricted to a subgroup of patients, especially with raised ALT and low to moderate levels of serum HBV DNA. To increase the rates of patients who may benefit from this treatment while minimizing the adverse events, a careful patient selection and individualized treatment decisions to achieve treatment optimization are required (A1).
  3.7.4 Full information about the advantages, adverse events and inconveniences of Peg-IFN versus NAs should be provided, so that the patient can participate in the decision (A1).
  3.7.5 Simultaneous combinations of Peg-IFN with NAs such as lamivudine, entecavir and tenofovir have been shown to be safe, but safety needs to be confirmed in larger studies before recommendation (B2).
  3.7.6 Sequential combination therapies using viral load reduction followed by addition of Peg-IFN or add-on Peg-IFN after response to NAs have been found to be safe with improved seroconversion rates compared to monotherapies (B2). These approaches need to be confirmed in larger studies.
  3.7.7 Finite-duration treatment with a NA is achievable for HBeAg-positive patients who seroconvert to anti-HBe on treatment. However, treatment duration is unpredictable prior to therapy, as it depends on the timing of HBeAg seroconversion and the treatment continuation post anti-HBe seroconversion (A1).
  3.7.8 Strategy of long-term treatment with NA(s) is necessary for patients who are not expected to or fail to achieve a sustained off-treatment virological response and require extended therapy, i.e., for HBeAg-positive patients who do not develop HBeAg seroconversion and HBeAg-negative patients (A1).
  3.7.9 The most potent drugs with the optimal resistance profile, i.e., tenofovir or entecavir, should be used as first-line long-term monotherapies (A1).
  3.7.10 As of yet, there are no data to indicate an advantage of de novo combination treatment with two NAs in NA-naive patients receiving either entecavir or tenofovir (C2).

### 3.8 Treatment failure to therapy and its management in chronic HBV infection

The goals of hepatitis B treatment are to eliminate or permanently suppress viral replication, normalize serum ALT levels and improve liver histology, thereby reducing the risk of disease progression in patients chronically infected with hepatitis B and reducing the long-term risk of liver-related complications such as HCC, decompensation and death. In recent years, the treatment of chronic hepatitis has greatly improved with the development of new therapeutic options. To date, two immunomodulators, interferon alpha and pegylated interferon, and five nucleos(t)ide analogues (NA), lamivudine, adefovir, entecavir, telbivudine and tenofovir (not all countries), are approved therapies for HBV.

The long term efficacy of NAs is determined by the ability to achieve and maintain viral suppression. Treatment failure may be either primary virological failure or secondary viral breakthrough. Primary virological failure may be either primary nonresponse or partial (suboptimal) virological response. Primary nonresponse is defined as <1 log reduction in plasma HBV DNA levels after 24 weeks of therapy. In the absence of noncompliance, primary nonresponse is rare and is now only observed during adefovir therapy due to suboptimal efficacy of this agent. The appropriate action is to switch to a more potent drug (entecavir in treatment-naïve patients, tenofovir in treatment-experienced patients). Partial virological response is defined as detectable HBV DNA in plasma after 24 weeks of therapy. Partial virological response may be encountered with all available NAs, especially in those patients with high baseline viraemia. The previous APASL HBV Management Guidelines recommended that treatment be modified (switch or add a second, more potent drug without cross-resistance) if HBV DNA remained detectable after 24 weeks. However, this “Roadmap Approach” really only pertains to patients receiving lamivudine or telbivudine (drugs with a low genetic barrier to resistance), and should become obsolete with the shift towards primary therapy with more potent drugs with a high genetic barrier to resistance. In patients receiving entecavir or tenofovir monotherapy with detectable HBV DNA after 24 weeks, continuation of the same treatment is recommended, given the steady rise in rates of virological response over time and the very low risk of resistance with both of these agents [213].

Viral breakthrough is either due to noncompliance or the emergence of drug resistance. Because antiviral therapy with NA does not completely inhibit the replication of the virus, the emergence of HBV drug resistance is almost inevitable with long-term monotherapy. Like HIV, the HBV reverse transcriptase lacks a proofreading function, which allows for viral mutations to occur spontaneously during viral replication. This results in a pool of viral quasi-species that coexist in different proportions depending on their relative replicative fitness. The dominant species at any one time is the “fittest” virus, capable of replicating in the presence of selection pressure provided by the antiviral therapy. Factors that may impact the risk of selecting resistant HBV variants during antiviral therapy include the baseline viral load and diversity, the replicative fitness of variants and the number of specific mutations that are required to confer resistance, which is the genetic barrier of that antiviral agent to resistance.

There are five NAs approved for clinical use, and a sixth agent, clevudine, which is approved in Korea but development elsewhere, has been halted because of risk of myopathy. All NAs target the active site of the HBV reverse transcriptase of the HBV polymerase and have potent antiviral activity, with between 4 and 6 log IU reduction in HBV DNA levels over 12 weeks. Single amino acid substitutions within the reverse transcriptase domain can significantly reduce NA binding and antiviral efficacy, whilst preserving replication capacity. The long-term benefit of these agents is lost following the selection of these resistant mutants, resulting in viral breakthrough and subsequent treatment failure.

Viral breakthrough due to drug resistance is defined as an increase in HBV DNA levels (≥1× log10 IU/ml) in patients who initially responded to antiviral therapy and are compliant with therapy [293]. This will lead to ALT elevations with occasional hepatitis flares and clinical decompensation. Occasionally, the emergence of drug resistance may result in acute liver failure and death, even in patients with minimal liver disease. Antiviral resistance is also associated with loss of long-term efficacy of antiviral therapy, with reduced HBeAg seroconversion and histological progression. Other potential consequences of NA resistance include changes to the overlapping envelope region, resulting in altered HBsAg antigenicity, possible surface escape mutants, reduced binding to HBIG and associated increased risk of HBV recurrence following liver transplantation.

**Fig. 4:**
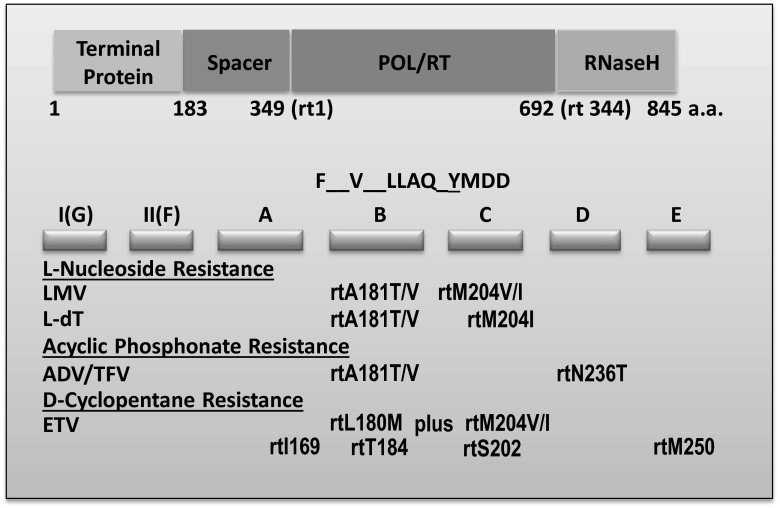
Reverse transcriptase mutations associated with drug resistance

Primary resistance mutations have been identified for five out of the six currently approved NAs (Fig. 4).

**Fig. 5:**
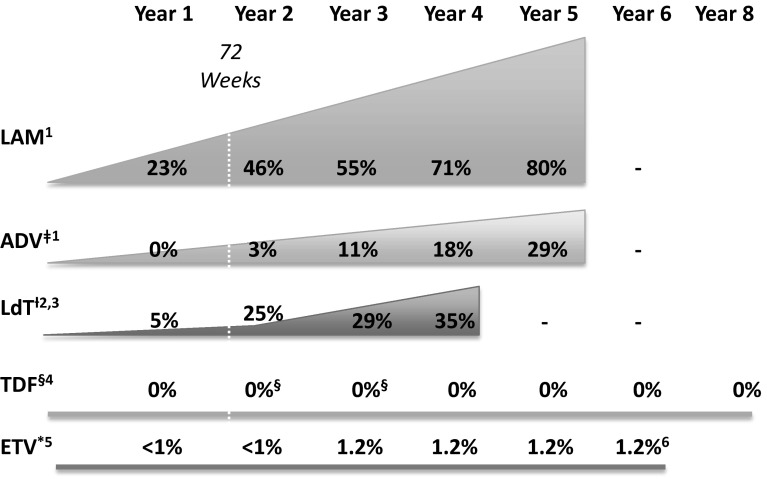
Cumulative incidence of antiviral resistance in long-term studies of NA therapy

Although all five currently available NAs target the same active site of the reverse transcriptase, they exhibit very different genetic barriers to resistance rates in long-term follow-up studies of each (Fig. 5).

*Lamivudine:l*-*nucleoside analogue* The first approved NA for HBV. Lamivudine has potent antiviral efficacy, but also has the lowest barrier to resistance. The cumulative rate of emergence of lamivudine resistance is 15–20 %/year, and it plateaus around 60 % after 5 years. Higher baseline viral load, HBeAg positivity and immunosuppression are all factors associated with increase rate of resistance, whilst HBV genotype and fibrosis stage are not. The primary mutations associated with lamivudine resistance are the rtM204I and rtM204V mutations (±rtL180M).

*Telbivudine:l*-*nucleoside analogue* Tenfold more potent than lamivudine. Slower rate of drug emergence than LAM, around 10 %/year in HBeAg-positive and 5 %/year in HBeAg-negative patients. In the Globe study, at the end of 2 years, resistance was observed in 21.6 % of HBeAg-positive patients and 8.6 % of HBeAg-negative patients. The primary mutations associated with telbivudine resistance are the rtM204I and rtM204V mutations. Therefore, lamivudine resistance is assumed to confer cross-resistance with telbivudine.

*Adefovir: acyclic nucleoside phosphonate* The first approved NA for the rescue of lamivudine resistance. It is also effective against telbivudine and entecavir resistance. Unfortunately, the dose-limiting nephrotoxicity of this agent has resulted in suboptimal dosing (10 mg) with reduced antiviral potency compared to other NAs. Around 20 % of patients have primary treatment failure to this agent. Factors that contribute to primary nonresponse include the inadequate dose of 10 mg, individual differences in ADV metabolism and prior lamivudine resistance. In treatment-naïve patients who had an adequate primary virological response, the rate of adefovir resistance is around 3–5 % per annum. This is increased to almost 10 % per annum in patients with prior lamivudine resistance (i.e., sequential monotherapy). The primary mutations associated with adefovir resistance are rt N236T ± rtA181V/T. The latter also confers cross resistance to lamivudine.

*Entecavir: deoxyguanosine analogue* 100-fold more potent than LAM and has a very high genetic barrier to resistance—only 1 % over 5 years in treatment-naïve patients. Much higher rates of resistance in LAM-experienced (refractory) patients, around 10 % per annum. This difference reflects the pathway to resistance for entecavir. The primary mutations are those associated with lamivudine resistance—L180M + m204I/V. However, secondary mutations are needed to confer resistance to entecavir. These include rtT184G ± rtS202I ± rtM250V. It has no cross resistance to adefovir, so entecavir monotherapy can be used to treat adefovir resistance.

*Tenofovir* Acyclic nucleoside phosphonate: 1000-fold more potent than adefovir and has a very high barrier to resistance. This is the only approved NA without any associated clinical resistance. Although reduced susceptibility to tenofovir has been produced in vitro with site-directed mutagenesis, no primary mutations associated with tenofovir resistance have been detected in any patient receiving up to 8 years continuous tenofovir therapy. In addition, no tenofovir resistance has been observed in patients with prior lamivudine resistance in studies of tenofovir salvage therapy. In a large Phase IIb study, 280 patients with documented resistance to lamivudine were randomized to either tenofovir monotherapy or the fixed-dose combination of tenofovir plus emtricitabine (an l-nucleoside analogue similar to lamivudine) for 96 weeks [198]. Both treatments were safe and well tolerated. The addition of emtricitabine did not improve efficacy—HBV DNA levels were suppressed below LOQ in 86 % of the combination group and 89 % of the monotherapy group. No tenofovir resistance was observed in either treatment group. Prior exposure or documented resistance to entecavir or adefovir was documented at baseline in 12 % and 22 %, respectively, all of who achieved and maintained complete viral suppression on tenofovir ± emtricitabine. In a second Phase IIb study, 105 patients with documented resistance to adefovir were randomized to tenofovir monotherapy or to tenofovir plus emtricitabine for 168 weeks [197]. Again, adding emtricitabine did not improve efficacy, with HBV DNA levels were suppressed below LOQ in 84 % of the combination group and 82 % of the monotherapy group maintaining HBV DNA levels below LOQ at the end of 168 weeks. The baseline genotypic resistance mutations did not predict response—in particular, the presence of lamivudine and/or adefovir resistance-associated mutations at baseline had no impact on long-term treatment response.

Because all the NAs share the same target (HBV polymerase), cross resistance is a major issue (Table 8), and therefore the emergence of resistance may limit future treatment options (Fig. 5). Therefore, the optimal first-line treatment will be with an NA with high antiviral potency and a high barrier to resistance. Unfortunately, in many countries within the Asia-Pacific region, the less expensive NAs with low barrier to resistance have remained as first-line therapies because of cost and access barriers. As patients receive and fail sequential monotherapy, multi-drug resistant HBV variants are becoming more prevalent, for which there are very limited salvage options available.

Combining two or more of these older NAs with low barriers to resistance from different classes may help delay or prevent the emergence of antiviral resistance to each drug (e.g., LAM plus ADV) [294]. However, such a strategy is associated with increased cost and non-adherence.

#### Summary

The best first-line strategy will always be selection of an agent with both a high barrier to resistance (requires multiple mutations before emergence of resistance) and high antiviral potency (achieves complete viral suppression within the first 6 months). Patient education and monitoring is also important to prevent treatment interruption.

The availability of tenofovir and entecavir as first line drugs has made the two previous APASL recommendations—(1) that combination of NAs without cross-resistance should be used in highest risk patients (those who have already failed one class, those with highest viral load and those on immunosuppression), and (2) that the treatment be modified (switch or add a second agent) after 24 weeks, if HBV DNA is still detectable (so-called “Roadmap Approach”)—invalid.

**Table 8:** Cross-resistance profiles amongst the five NAs [332]

Pathway	HBV variants	LAM	LdT	ETV	ADV	TDF
	Wild-type	**S**	**S**	**S**	**S**	**S**
l-Nucleoside (LAM/LdT)	M204 l/V	*R*	*R*	***I***	**S**	**S**
Acyclic phosphonate (ADV)	N236T	**S**	**S**	**S**	*R*	***I***
Shared (LAM, LdT, ADV)	A181T/V	*R*	**S**	**S**	*R*	***I***
Double (ADV, TDF)	A181T/V + N236T	*R*	*R*	**S**	*R*	*R*
d-Cyclopentane (ETV)	L181M + M204V/I ± I169 ± T184 ± S202 ± M250	*R*	*R*	*R*	**S**	**S**
Multi-drug resistance	A181T + N236T + M204V	*R*	*R*	*R*	*R*	*R*

In patients receiving long-term therapy with lamivudine, telbivudine and adefovir monotherapy, appropriate virological monitoring should be performed to detect viral breakthrough and genotypic resistance. Early detection and modification of antiviral therapy should optimize long-term outcomes (Table 9).

**Table 9:** Strategies to manage treatment failure—first and second line

LAM/LdT resistance	Switch to TDF
	Add ADV
LAM then ETV resistance	Switch to TDF
	Add ADV
ADV resistance (no previous LAM)	Switch to ETV
	Switch to TDF
ADV resistance (previous LAM/LdT)	Switch to TDF
	Switch to LAM/TDF
ETV resistance (no previous LAM/LdT)	Switch to TDF
	Add ADV
Multidrug resistance	Switch to ETV/TDF
	Switch to Peg-IFN

3.8 Recommendations: treatment failure to therapy and its management in chronic HBV infection
  3.8.1 The best strategy for drug resistance is prevention through patient education on compliance and selection of an agent with high potency and high barrier to resistance (entecavir and tenofovir) (A1).
  3.8.2 Regular monitoring for viral breakthrough should be performed in patients receiving an agent with low barrier to resistance (lamivudine, telbivudine and adefovir) (A1).
  3.8.3 Patients with viral breakthrough evident by more than 1 log IU/ml increase of HBV DNA from the nadir should be counseled about compliance. In the compliant patient, appropriate testing to confirm genotypic drug resistance should be performed with a validated test. Rescue therapy should be instituted as early as possible in case of drug resistance (A1).
  3.8.4 For patients who develop drug resistance while on LAM or LdT, switching to TDF is indicated (A1).
  3.8.5 For patients who develop drug resistance while on ADV therapy, without prior lamivudine exposure, switching to either ETV or TDF monotherapy is indicated (A1).
  3.8.6 For patients who develop drug resistance while on ADV rescue therapy for prior lamivudine/telbivudine resistance, switching to TDF monotherapy is indicated (B1).
  3.8.7 For patients who develop drug resistance while on ETV, switching to TDF is indicated (B1).
  3.8.8 For patients who develop drug resistance associated with multidrug resistant mutations (A181T + N236T + M204V), combination ETV plus TDF is indicated (C2).

### 3.9 Treatment of patients with chronic HBV infection with severe liver disease

#### 3.9.1 Treatment of patients with compensated cirrhosis

Peg-IFN in regimens similar to those used in CHB can be used for the treatment of well-compensated cirrhosis [258]. Among NAs, monotherapies with tenofovir or entecavir are preferred because of their potency and minimal risk of resistance. Close monitoring of HBV DNA levels every 3 months during the first year of therapy and until HBV DNA undetectability is important, as exacerbations of hepatitis B may occur in patients with cirrhosis requiring urgent management. Thus, patients with cirrhosis require long-term therapy, with careful monitoring for resistance and flares.

Clinical studies indicate that prolonged and adequate suppression of HBV DNA can stabilize patients and prevent the progression to decompensated liver disease [86]. Regression of fibrosis and even reversal of cirrhosis have been reported in patients with prolonged suppression of viral replication [295].

Nonetheless, long-term monitoring for HCC is mandatory despite virological remission under NA(s), since there is still a risk of developing HCC [296, 297].

NA therapy should usually be continued for life in cirrhotic patients.

3.9.1 Recommendations: treatment of patients with compensated cirrhosis
  3.9.1.1 Peg-IFN in regimens similar to those used in CHB can be used for the treatment of well-compensated cirrhosis (A1). However, extra caution and monitoring is recommended to prevent and diagnose hepatic decompensation (A1).
  3.9.1.2 Among NAs, monotherapies with tenofovir or entecavir are preferred (A1).
  3.9.1.3 NA therapy should usually be continued for life in cirrhotic patients (B1).
  3.9.1.4 Monitoring for HCC is mandatory despite virological remission under NA(s) (A1).

#### 3.9.2 Treatment of patients with decompensated cirrhosis

Patients with decompensated cirrhosis should be treated in specialized liver units, as the application of antiviral therapy is complex, and these patients may be candidates for liver transplantation. Antiviral treatment is indicated irrespective of HBV DNA level, in order to prevent reactivation. Peg-IFN is contraindicated in this setting. Entecavir or tenofovir should be used. The licensed entecavir dose for patients with decompensated cirrhosis is 1 mg (instead of 0.5 mg for patients with compensated liver disease) once daily.

Recent studies have shown that both drugs are not only effective, but are generally safe in these patients [164, 298].

Lactic acidosis has been reported to develop with some NAs, particularly entecavir, in treated patients with advance decompensated cirrhosis (MELD score >20). Therefore, clinical and laboratory parameters should be closely monitored in this setting. The dose of all NAs needs to be adjusted in patients with low creatinine clearance (<50 ml/min).

Patients with decompensated cirrhosis may show slow clinical improvement over a period of 3–6 months under NA(s) and then transplantation may be avoided. In such cases, life-long treatment is recommended. The HCC risk is high in these patients even under effective NA therapy, and therefore long-term HCC surveillance is mandatory [299]. Some patients with advanced hepatic disease with a high Child–Pugh or MELD score may have progressed beyond the point of no return, and may not benefit, thus requiring liver transplantation. In that situation, treatment with NA(s) inducing HBV DNA undetectability at transplantation will decrease the risk of HBV recurrence in the graft (see “3.12 Prevention and treatment of recurrent hepatitis B after liver transplantation” section).

3.9.2 Recommendations (treatment of patients with decompensated cirrhosis)
  3.9.2.1 Patients with decompensated cirrhosis should preferably be treated in specialized liver units, as the application of antiviral therapy is complex, and these patients may be candidates for liver transplantation (A1).
  3.9.2.2 Antiviral treatment is indicated in all HBsAg positive cirrhotic patients with hepatic decompensation, irrespective of HBV DNA levels (A1).
  3.9.2.3 Peg-IFN is contraindicated in decompensated cirrhosis (A1).
  3.9.2.4 Among NAs, monotherapies with tenofovir or entecavir are preferred (A1). The antiviral treatment should not be delayed while waiting for the HBVDNA results.
  3.9.2.5 The dose of all NAs needs to be adjusted in patients with low creatinine clearance (<50 ml/min) (A1).
  3.9.2.6 NA therapy should usually be continued for life in decompensated cirrhotic patients (B1).
  3.9.2.7 Monitoring for HCC is mandatory, despite virological remission under NA(s) (A1).

### 3.10 Treatment of patients with reactivation of chronic HBV infection including those developing acute on chronic liver failure

Upon exposure to HBV, individuals with a vigorous and broad immune response to the virus develop an acute self-limited infection that may result in acute hepatitis. Individuals who do not mount a broad and vigorous immune response do not clear the virus, but develop persistent infection and become chronically infected with HBV. HBV persists in the body even after serological recovery from acute hepatitis B; so individuals who have been exposed to HBV are at risk for reactivation of hepatitis B replication when the immune imbalance occurs, which can lead to flare or exacerbation of hepatitis [300]. The severity of the flare depends on the state of underlying liver disease and may range from mild flare of hepatitis to acute on chronic liver failure. As patients suffering from severe acute exacerbation of CHB may not have underlying liver cirrhosis, they may recover to a relatively normal liver function, in contrast to those suffering from end-stage liver cirrhosis. It is therefore important to recognize this important clinical presentation of CHB.

Reactivation of chronic HBV infection has two components, i.e., reactivation of HBV replication and flare (or exacerbation) of hepatitis. Reactivation of HBV replication should be defined as a marked increase in HBV replication (≥2 log increase from baseline levels or a new appearance of HBV DNA to a level of ≥100 IU/ml) in a person with previously stable or undetectable levels or detection of HBV DNA with levelss ≥20,000 IU/ml in a person with no baseline HBV DNA [22, 300]. The types of reactivation should be described as follows: exacerbation of CHB or reactivation of past hepatitis B. The latter can be further defined as reverse HBsAg seroconversion (reappearance of HBsAg), or appearance of HBV DNA in serum in the absence of HBsAg.

This reactivation of HBV replication may lead to flare (or exacerbation) of hepatitis, which is characterized by an abrupt elevation of the serum ALT level, although there is no consensus definition or diagnostic criterion. It usually refers to an abrupt increase in serum ALT to >5 times the upper limit of normal and more than twice the baseline value [23, 301]. Severe hepatitis flare means reactivation with the presence of coagulopathy with prolonged prothrombin time (prolonged by more than 3 s) or INR increased to >1.5. Severe hepatitis flare may lead to ACLF. Flare (or exacerbation) of hepatitis in CHB infected patients is common and may be caused by a number of factors (Table 10).

**Table 10:** Causes of acute hepatitis flares of hepatitis in chronic hepatitis B virus infected patients

Spontaneous reactivation of hepatitis B virus replication
Due to immunosuppressive medications: cancer chemotherapy, antirejection drugs, corticosteroids
Cessation of anti-viral agents
Emergence of drug resistance
Due to antiviral therapy: interferon, corticosteroid withdrawal
Due to superimposed infections with other hepatotropic viruses: hepatitis A/E virus, hepatitis C virus, hepatitis delta virus
Caused by interaction with HIV infection: reactivated hepatitis, effect of immune reconstitution therapy
Other hepatotropic insults: drugs, alcohol

#### Spontaneous reactivation hepatitis B

Spontaneous reactivation of hepatitis B can occur in both HBeAg-positive and -negative patients [302, 303]. Spontaneous reactivation of chronic HBV infection can occur in the immune clearance phase affecting 40–50 % of HBeAg-positive patients, and can be prolonged when there is repeated unsuccessful clearance of HBeAg [304]. Reactivation of chronic HBV infection at the HBeAg-negative phase is seen in 15–30 % of HBeAg-negative patients, and is occasionally associated with HBeAg seroreversion [301].

In Far Eastern regions, 23–38 % of patients have been reported to develop jaundice and hepatic decompensation (acute on chronic liver failure) during biochemical exacerbation of CHB [305, 306]. These exacerbations may be associated with significant mortality.

#### Pathogenesis of spontaneous reactivation of hepatitis B virus infection

Acute hepatitis flare is precipitated by the reactivation of HBV infection. The reasons for reactivated infection are unknown, but are likely explained by changes in the immunological control of viral replication.

Influence of HBV genotypes on reactivation has also been assessed. There is a possibility that the immunogenicity of the different genotypes is different. Genotype B HBV may associate with more vigorous immune response that leads to a higher chance of successful immune clearance, but also a higher risk of hepatic decompensation during the hepatitis flare. On the contrary, genotype C HBV is associated with less vigorous and prolonged, abortive immune clearance, which is more likely to cause progressive liver damage, and eventually, liver cirrhosis and HCC [307].

Several HBV mutant strains, including mutations in precore, core promoter, and deletion mutation in pre-S/S genes, have been reported. Viral populations in the immune tolerance phase mostly consist of exclusively wild-type virus or HBeAg-positive strains with little or no precore/core promoter mutants or HBeAg-negative strains [308]. Spontaneous reactivation of CHB may also occur in response to HBV genotypic variation. Chronic infection with precore mutant is often associated with multiple flares interspersed with periods of asymptomatic infection [309]. It is possible that the absence of HBeAg in patients harboring precore mutant HBV may permit a more vigorous immunological response to core peptides expressed on the surface of hepatocytes. Episodic flares have been attributed to increases in the concentration of precore mutants and changes in the proportion of precore to wild-type HBV [310]. It has been suggested that disease exacerbations are uncommon during the earliest phase of chronic HBV at a time when wild-type HBV predominates, and that flares become common with the gradual emergence of the precore variant [310]. These flares have been thought to subside with time as the genetic heterogeneity disappears and patients become exclusively infected with precore HBV [311]. Multiple exacerbations of hepatitis due to reactivated HBV infection have been described in patients with BCP mutation, either alone, or in association with precore mutation [312, 313].

Reactivation seems to occur more commonly in male homosexuals, patients who are infected with human immunodeficiency virus (HIV), concurrent with bacterial infections or surgery, and when there is emotional or physical stress [314]. Pregnancy and postpartum may also be a risk factor [315]. Liver injury during these spontaneous flares appears to be mediated by expanded numbers of T cells that are reactive to HBeAg and HBcAg which are cross-reactive at the T cell level. Measurement of lymphocyte proliferation in response to these viral antigens has shown that increased T-cell responses occur in the early phase of acute flares and subside after recovery from acute exacerbation and HBeAg seroconversion [316].

Once acute on chronic liver failure (ACLF) develops, the immunological changes seen in the inflammatory process are very similar to those of severe sepsis [317]. As the ACLF progresses, the resulting inflammatory responses in the liver and its associated cellular immune dysfunction can result in multi-organ failure.

#### Diagnosis

The typical presentation of severe spontaneous reactivation in a patient with CHB is a short onset of jaundice and very high ALT level, sometimes preceded by prodromal constitutional symptoms. If signs of chronic liver disease are present, the diagnosis could be easy, however, some patients presenting with severe acute reactivation of CHB may not have had an earlier diagnosis of chronic HBV infection. In countries with intermediate and high endemicity, the possibility of reactivation of chronic HBV infection is high, which may be the first presentation of CHB or compensated cirrhosis, which was asymptomatic before exacerbation. Hence, a possibility exists that a proportion of patients with suspected acute hepatitis B might actually be suffering from CHB and manifesting clinically for the first time during a period of severe reactivation [23]. In areas of intermediate to high HBV endemicity, endemic for chronic HBV infection, reactivation (flare or exacerbation) accounts for 27–70 % of presumed acute hepatitis [23, 317, 318].

The symptoms and biochemical parameters of severe acute reactivationof CHB can be very similar to those of acute hepatitis B [23]. Hence, severe acute reactivation of CHB might be misdiagnosed as acute hepatitis B in some cases. Patients with severe spontaneous acute reactivation of CHB can have positive IgM anti-HBc, which may again be confused with the diagnosis of acute hepatitis B. Levels >600 Paul–Ehrlich units/ml or IgM anti-HBc (>1:1000) suggest an acute HBV infection with high inflammatory activity. In all other situations, concentrations are lower or undetectable [23, 319]. One study suggests that a low titer of IgM anti-HBc (<1:1000) and high HBV DNA level (>0.5 pg/ml, which equals ~141,500 copies/ml) are useful to identify severe acute reactivation (flare or exacerbation) of CHB from acute hepatitis B [23]. However, HBV DNA may sometimes become undetectable at the peak of the biochemical exacerbation due to vigorous immune clearance. The presence of BCP mutation and precore stop codon mutations have been suggested to differentiate severe acute exacerbation of CHB from acute hepatitis B in Japanese series, but its use in clinical practice needs further validation [319].

A previous history of CHB or a positive family history of CHB may suggest reactivation (flare or exacerbation); whereas recent history of at-risk blood, percutaneous or sexual exposure may suggest acute hepatitis B.

Liver biopsy showing evidence of chronicity may suggest chronic infection.

In uncertain cases of acute hepatitis B versus severe reactivation of CHB, one can manage these patients as severe reactivation cases and repeat hepatitis B surface antigen testing (HBsAg) 6 months later. In over 95 % of acute hepatitis B acquired in adulthood, HBsAg will be cleared on the follow-up testing; however, a small percentage of patients with acute reactivation of chronic HVB infection may also clear HBsAg.

As CHB infected patients still can acquire another viral infection that causes acute hepatitis, other viral hepatitis (A, C, D and E) must be excluded by serological assays. If suspected, other etiologies (Table 1) should also be excluded before a diagnosis of spontaneous reactivation of CHB is made.

#### Outcome

The clinical presentation of acute spontaneous reactivation of CHB infection depends on the underlying severity of liver disease and other factors.

In a Chinese study on evaluation of prognostic factors in severe reactivation (flare or exacerbation) of chronic HBV infection, at admission the following parameters were independently associated with adverse outcome: pre-existing cirrhosis, high Child–Pugh score, low albumin level, high bilirubin level, prolonged PT and low platelet count. For the subsequent stay in the hospital, these factors were as follows: high peak bilirubin level, long peak PT, long duration to reach the peak PT, development of encephalopathy, and presence of ascites. There was also a trend for a longer time to reach peak bilirubin level to be an independent factor associated with adverse outcome [320].

In one study from Taiwan on HBeAg-positive noncirrhotic patients with acute exacerbation, 5.1 % of the exacerbation episodes resulted in hepatic decompensation, and serum HBV DNA level was the only significant risk factor (*p* = 0.003). A serum HBV DNA cutoff value of 1.55 × 10^9^ copies/ml predicted decompensation with a sensitivity of 85.7 %, a specificity of 85.5 %, a negative prediction value of 99.1 %, and a positive prediction value of 24.0 % [321].

Owing to their limited hepatic reserve, cirrhotic patients are expected to recover more slowly from the hepatic insult and are more prone to complications including sepsis, gastrointestinal bleeding and acute renal failure. Many studies have found that patients with pre-existing liver cirrhosis and more serious hepatic dysfunction (prolonged prothrombin time, elevated serum bilirubin and high Child–Pugh score) have a higher risk of mortality [322, 323].

Once the disease reaches the stage of acute on chronic liver failure (ACLF), the prognosis is extremely poor, with 3-month mortality rates without liver transplantation reported to be around 50–55 % [324]. Different predictive models have been used in prognosticating acute-on-chronic liver failure due to reactivation of CHB. MELD is the most commonly used prediction model. MELD score has been found in many studies to be more objective when compared to Child–Pugh score in predicting survival in chronic HBV infection patients with ACLF [325, 326]. It has been found that a MELD score of >30 is associated with high mortality (>90 % despite using antivirals), a MELD <20–23 is associated with low mortality with use of antivirals (16–17 %) and MELD in between these ranges is associated with intermediate mortality (44–51 %) with antiviral treatment [327, 328].

A number of logistic regression models based on both laboratory parameters and organ dysfunction have also been described. One regression model, using the presence of hepatorenal syndrome, liver cirrhosis, positive HBeAg, low albumin and prolonged PT, was found to be superior to the MELD score in predicting 3-month mortality [329]. Another model based on the presence of hepatic encephalopathy, hepatorenal syndrome, positive HBeAg, liver cirrhosis and prolonged PT was also found to be superior to both the MELD and Child–Pugh score [325]. In a recent study from China compared a logistic regression based model (based on presence of hepatic encepahalopathy, hepatorenal syndrome, cirrhosis, HBeAg status, Prothrombin time and age) with Child–Turcotte–Pugh (CTP) classification, King’s College Hospital (KCH) criteria, model for end-stage liver disease (MELD), MELD combined with serum sodium (Na) concentration (MELDNa), and integrated MELD (iMELD) for predicting short-term prognosis of patients with HBV-related acute-on-chronic liver failure (ACLF). It was found that the regression model, MELD, MELDNa and iMELD had similar accuracy in predicting the short-term prognosis in patients with liver cirrhosis, while regression model was superior to MELD, MELDNa and iMELD in predicting the short-term prognosis of HBV-ACLF patients without liver cirrhosis. CPT score and KCH criteria fared poorly [330]. Further studies to externally validate these models would be needed.

Acute Physiology and Chronic Health Evaluation (APACHE) II and III, Simplified Acute Physiology Score (SAPS) II, and Mortality Prediction Model II, SOFA and its modifications have been used to prognosticate critically ill patients with liver failure [331, 332].

#### Treatment

Patients need intensive supportive care, including close monitoring and treatment of complications.

In severe spontaneous reactivation of CHB when immune activity is already excessive, interferon-based treatment may aggravate the hepatic decompensation, and is thus contraindicated. Oral nucleos(t)ide analogs are the treatment of choice.

In initial case series or cohort studies of Lamivudine in patients with severe acute exacerbation, some showed dramatic effects [333], whereas others could not demonstrate any survival benefit of lamivudine treatment [323, 334, 335], possibly related to the delayed commencement of lamivudine. A study from Taiwan suggests that the beneficial effect of antiviral therapy on short-term survival depends on the timing of treatment. Among consecutive CHB patients with severe acute exacerbation treated with lamivudine, all 25 patients who had baseline bilirubin below 20 mg/dl survived. Among patients with low (<20 mg/dl) baseline serum bilirubin level, lamivudine treatment has definite survival benefit as compared to historic controls who did not receive lamivudine (5/20 patients died, 20 %, *p* = 0.013). On the other hand, the mortality rate of the patients who received lamivudine when bilirubin was above 20 mg/dl (23/35, 67 %) was similar to that of the untreated historical controls (9/11, 82 %) [336]. A more recent study found a survival benefit in lamivudine-treated patients when compared to controls in patients with a MELD score of 30 or less; however, those treated with lamivudine still had a 3-month mortality of 50.7 %. A low pre-treatment HBV DNA and a rapid decline in viral load were predictors of good outcome [337].

Once ACLF develops, the prognosis of spontaneous reactivation of HBV infection is poorer as compared to patients who don’t develop features of ACLF. In one meta-analysis of antiviral therapy in ACLF due to spontaneous reactivation of HBV infection that included 11 randomized controlled trials (including 654 patients; 340 treated with NAs such as lamivudine entecavir, telbivudine, or tenofovir disoproxil fumarate, and 314 treated with NAs or placebo), it was found that nucleoside analogues significantly improved 1-month [OR 2.10; 95 % CI (1.29, 3.41); *p* = 0.003], 3-month [OR 2.15; 95 % CI (1.26, 3.65); *p* = 0.005] and 12-month survival [OR 4.62; 95 % CI (1.96, 10.89); *p* = 0.0005] [338]. Another meta-analysis of five studies on nucleos(t)ide analogues in ACLF due to spontaneous reactivation of HBV infection concluded that antiviral treatment with nucleos(t)ide analogues significantly lowered 3-month mortality [44.8 vs. 73.3 %, RR 0.68, 95 % CI (0.54, 0.84), *p* < 0.01] as well as incidence of reactivation [1.80 vs. 18.4 %, RR 0.11, 95 % CI (0.03, 0.43), *p* < 0.01] compared to those who did not. There was no significant difference in the prognosis of patients treated with entecavir or lamivudine [36.4 vs. 40.5 %, RR 0.77, 95 % CI (0.45, 1.32), *p* = 0.35] [339].

Several studies have found that despite a faster suppression of HBV replication, entecavir treatment was either not associated with improved short-term survival as compared to patients receiving no treatment [340], or had higher overall mortality as compared to lamivudine treatment [341], or higher mortality when treatment was started early but with high DNA levels (bilirubin <15 mg/dl and HBV DNA higher than 10^5^ copies/ml) compared with lamivudine [342]. Lactic acidosis has been hypothesized as a possible cause of increased mortality with entecavir [341]. This finding needs further confirmation. However, other studies have found comparable efficacy of entecavir and lamivudine in the short term [329, 343, 344], and long term [345], or better long-term (52 weeks) survival but not short-term survival as compared to lamivudine [346]. One meta analysis found that there was no significant difference in the prognosis of patients treated with entecavir or lamivudine [36.4 vs. 40.5 %, RR 0.77, 95 % CI (0.45, 1.32), *p* = 0.35] [339]. One study has found entecavir to have similar survival benefit as compared to telbivudine, although telbivudine had a better renoprotective effect [347].

One RCT from India found improved 3-month survival with tenofovir (57 %) in comparison to placebo (15 %) among patients with acute exacerbation of chronic HBV infection presenting as acute-on-chronic liver failure. A more than 2-log reduction in HBV DNA levels at 2 weeks was found to be an independent predictor of survival [348].

In one study, 69 patients of severe spontaneous reactivation of hepatitis B were randomized to receive either tenofovir monotherapy or dual therapy of tenofovir plus telbivudine. Of all patients, 25 patients had ACLF (13 patients received tenofovir and 12 received tenofovir plus telbivudine). Patients with ACLF receiving tenofovir plus telbivudine against tenofovir alone had significant improvement in MELD score at week 4 and week 12 and improvement in acute kidney injury compared to baseline. Of the 69 patients enrolled into study, 11 patients died at the end of the 3-month follow-up period. Among ten deaths in ACLF, eight had received tenofovir alone (*p* = 0.02). A predictor of mortality in univariate analysis in ACLF-B at 24–36 weeks of follow-up was presence of septic shock, tenofovir monotherapy, e antibody positivity and high baseline MELD score [349].

The definitive treatment for severe reactivation (flare or exacerbation) with ACLF is liver transplantation. Both deceased and living donor transplants are viable and very useful options with very good results [350]. Liver transplantation results from the East in patients with HBV reactivation have shown successful 5-year survival above 90 % [350, 351].

In a DDLT setting, the availability of the organ becomes a major concern. In living donor transplant cases, there are no waiting list constraints, and survival has been shown to be comparable to DDLT.

Recently, a lot research has been conducted in an attempt to improve the dreadful outcome in HBV ACLF. One randomized placebo-controlled trial found that the administration of granulocyte-colony stimulating factor improved survival after 2 months [352]. Use of bioartificial liver support systems is controversial and the results of a randomized controlled multicenter study in ACLF patients failed to identify any survival benefit [353]. Corticosteroids, based on their anti-inflammatory activity, have been used in chronic HBVinfection with ACLF. In a recent study, 56 patients received intravenous dexamethasone 10 mg daily for 5 days, together with continuous lamivudine. When compared with controls, dexamethasone treatment was an independent factor influencing survival, with a rapid decline in serum bilirubin in the first 5 days being predictive of survival [354]. In a more recent study, corticosteroid treatment in combination with nucleotide analogue has sufficient virological effect against severe acute exacerbation of chronic HBVinfection, and a rapid decline of HBV DNA is conspicuous in survived patients [355].

3.10 Recommendations: treatment of patients with reactivation of chronic HBV infection, including those developing acute on chronic liver failure
  3.10.1 Reactivation of HBV replication should be defined as a marked increase in HBV replication (≥2 log increase from baseline levels or a new appearance of HBV DNA to a level of ≥100 IU/ml) in a person with previously stable or undetectable levels or detection of HBV DNA, with levels ≥20,000 IU/ml in a person with no baseline HBV DNA (B1).
  3.10.2 Flare (or exacerbation) of hepatitis usually refers to an abrupt increase in serum ALT to >5 times the upper limit of normal and more than thrice the baseline value (B1).
  3.10.3 Other causes of hepatitis flares, such as superimposed hepatotropic viruses, toxins or drugs, should be excluded (Table 10) (A1).
  3.10.4 The severity of such reactivation depends on the severity of underlying liver disease, and once ACLF develops, the prognosis is very poor (A1).
  3.10.5 Nucleos(t)ide analogs should be started immediately without delay or waiting for the HBV DNA results (A1).
  3.10.6 Liver transplantation should be considered among patients with severe liver failure (e.g., MELD >30) (B1).
  3.10.7 Assessment of reduction of HBV DNA level at week 2 after nucleos(t)ide analogs should be done; if there is a <2 log reduction, it suggests poor prognosis and the patient should be considered for liver transplantation (B1).

### 3.11 HCC screening in chronic HBV infection

HCC screening and surveillance in patients with HBV infection have been covered in detail in APASL consensus recommendations on HCC [356].

More than 50 % of HCC cases worldwide and 70–80 % of those in HBV-endemic regions are attributable to chronic HBV infection [357]. The relative risk of HCC in chronic HBV-infected subjects was about 100–223 times that of normal population [358]. As a result, surveillance for HCC has been widely applied in patients with chronic HBV infection.

An important issue related to the surveillance program is cost-effectiveness. In many Western countries, interventions that can be achieved at a cost of <$50,000/year of life gained are considered cost-effective [359]. Obviously, this threshold cost is not applicable in most Asian countries, and should be determined depending on the economic situation of each country. As a matter of course, the efficacy of surveillance unambiguously depends on the incidence of HCC in the target population.

#### Who should be screened?

In determining the target population for surveillance, two points should be taken into consideration: the incidence of HCC, and the degree of benefit from a treatment in terms of patient’s survival. According to several cost-effectiveness models, surveillance becomes cost-effective when the risk of HCC is 1.5 %/year or greater in patients with cirrhosis [359, 360]. However, surveillance with USG and AFP becomes cost-effective once the incidence of HCC exceeds 0.2 %/year in hepatitis B infected subjects without cirrhosis [361].

All patients with HBV-related cirrhosis should be screened for HCC. However, the benefit of surveillance seems to be absent or minimal in Child–Pugh class C patients. Trevisani et al. [362] reported that a surveillance program could prolong the patient’s survival in Child–Pugh class B patients. However, in Child–Pugh class C patients, although cancer stage and treatment distribution were better in those under a surveillance program than those without it, there was no difference in overall survival (7.1 vs. 6.0 months). The anticipated survival benefit from early detection of HCC was offset by a high incidence of liver-failure-related mortality.

Defining the population who should be screened among chronic HBV-infected subjects without cirrhosis is somewhat complicated. As mentioned above, surveillance becomes cost-effective in chronic HBV-infected subjects without cirrhosis, if the cutoff cost-benefit is $50,000/year of life gained and the incidence of HCC exceeds 0.2 %/year. However, each Asian country differs greatly in the economic situation, and therefore the result of cost-effectiveness analysis performed in a specific country is not applicable to other countries. Since the cost-effectiveness greatly depends on the incidence of HCC, the threshold incidence of HCC for surveillance should be determined individually in each country.

##### Outcome calculators for predicting HCC

Until now, several prediction scores have been developed and validated to calculate the risk of HCC in patients with chronic HBV infection in the community and clinic settings.

##### Liver stiffness as predictor of HCC development

Liver stiffness, measured by transient elastography, has been used to assess the degree of liver fibrosis and it correlates well with liver fibrosis stage. Jung et al. [363] reported that the incidence rates of HCC are significantly associated with the degree of elevated liver stiffness measurement (LSM). The discordance rate in the diagnosis of cirrhosis between clinical criteria and LSM was 13.4 %, and the incidence of HCC was higher in patients without clinical cirrhosis who showed LSM >13 kPa than in those with clinical cirrhosis who showed LSM ≤13 kPa. These results strongly suggested that LSM can be a complement or alternative to the clinical diagnosis of cirrhosis in developing models for the prediction of HCC. However, LSM per se was not useful in determining the subgroup of patients for surveillance in this study population. The observed incidence of HCC was 0.54 %/person-year even in patients with the lowest LSM value (<8 kPa), which is much higher than the threshold incidence (0.2 %/year) for surveillance in noncirrhotic chronic HBV-infected subjects. Recently, Wong et al. [364] modified their CU–HCC score with LSM (LSM–HCC score), and the AUROCs of LSM–HCC score were higher than those of CU–HCC score (0.83–0.89 vs. 0.75–0.81). By applying the cutoff value of 11, the score excluded future HCC with high negative predictive value (99.4–100 %) at 5 years.

#### Modalities and frequency for screening

USG, AFP, des-γ-carboxyprothrombin (DCP, prothrombin induced by vitamin K absence-II), *Lens culinaris* agglutinin-reactive fraction of AFP (AFP-L3), or their combinations have long been used as surveillance tests for HCC in Asian countries. Detailed review on the diagnostic performance of each test as a surveillance test is beyond the scope of this guideline for the management of CHB. They were well summarized in APASL consensus recommendations on HCC [356].

The APASL consensus recommendations on HCC recommended USG and AFP every 6 months as surveillance tests for HCC [356].

3.11 Recommendations: HCC screening in chronic HBV infection
  3.11.1 Surveillance for HCC is recommended in high-risk populations with chronic HBV infection (B2).
  3.11.2 Current HCC risk prediction scores can accurately stratify the risk of HCC in patients with chronic HBV infection and be used to determine the target population for surveillance (B1).
  3.11.3 The threshold incidence of HCC for surveillance should be determined individually based on the economic situation of each country (B1).
  3.11.4 Surveillance for HCC should be performed by USG and AFP (B2).
  3.11.5 Surveillance by USG and AFP should be performed every 6 months (B2), and preferably every 3 months in cirrhotics and those at high risk of HCC (C2).
  3.11.6 Contrast enhanced CT and MRI should be used regularly for confirmation of suspicious lesions on US screening (A1). Their use is also recommended in the screening of patients with advanced cirrhosis with high suspicion of development of HCC (C2).
  3.11.7 A baseline CECT or CEMRI should be obtained in all cirrhotics at presentation (B1).

### 3.12 Prevention and treatment of recurrent hepatitis B after liver transplantation

Antiviral therapy using newer nucleos(t)ide analogues with lower resistance rates such as entecavir or tenofovir could suppress HBV replication, improve liver function, and delay or obviate the need for liver transplantation in some patients. Antiviral therapy before LT may prevent HBV recurrence after LT by reducing the level of viremia to extremely low levels. After LT, the primary goal of antiviral therapy is to prevent HBV recurrence and to prevent graft loss.

#### Diagnosis, mechanisms, and risk factors for HBV recurrence after LT-

Recurrence of HBV infection after LT is defined as the reappearance of circulating hepatitis B surface antigen (HBsAg) with or without detectable HBV DNA. However, only patients who develop persistently detectable HBV DNA are shown to be at risk for clinical disease and graft loss [365]. HBV reinfection is the consequence of an immediate reinfection of the graft by circulating HBV particles, or a later reinfection from HBV particles coming from extrahepatic sites such as peripheral blood mononuclear cells, or both.

There is a direct relationship between HBV viral load at transplantation (i.e., >10^5^ copies/ml) and the rate of HBV recurrence [366]. Thus, antivirals should be used before transplantation to achieve undetectable HBV DNA levels to reduce the risk of HBV recurrence. Other factors associated with low rates of recurrence include surrogate markers for low levels of viral replication (including HBeAg-negative status, fulminant HBV, and HDV coinfection). In addition, HCC at LT, HCC recurrence, or chemotherapy used for HCC are independently associated with an increased risk of HBV recurrence [367].

#### Prevention of HBV recurrence after LT-

Prior to the availability of effective HBV prophylaxis in the 1980s, LT for CHB was a relative contraindication. High rates of graft reinfection leading to severe flares and loss of graft occurred in the absence of antiviral therapy. The use of hepatitis B immune globulin (HBIG) after LT was the first major milestone in the prevention of post-transplant HBV recurrence. HBIG monotherapy reduced HBV recurrence by a rate of approximately 70 % [368]. The advent of antiviral therapy further changed the landscape of post-LT prophylaxis. Several meta-analyses have shown that combination prophylaxis was significantly superior to antivirals or HBIG alone in preventing HBV recurrence [369–371].

##### HBIG containing prophylaxis regimens

In conventional protocols, HBIG is used at high dose to neutralize HBsAg during the anhepatic phase and the first postoperative week (i.e., generally 10,000 IU/day) . In the early post transplant period, some studies reported that high IV HBIG dosage (≥10,000 IU/day) versus low HBIG dosage (<10,000 IU/day) was associated with a lower frequency of HBV recurrence [368]. In medium-term and long-term follow-up, IV HBIG has been administered in two different ways: at a frequency dictated by the maintenance of specific anti-HBs levels, or on a fixed schedule. The latter approach is simpler and requires less monitoring, but is more expensive [372]. The target levels for anti-HBs titers vary with time after LT: generally, anti-HBs levels are maintained at >500 IU/l during 1–3 months, >250 IU/l until 6–12 months, and at >100 IU/l thereafter.

The use of IV HBIG has limitations; namely, the high cost, parenteral administration, limited supply, need for frequent clinic visits and laboratory monitoring, lower effectiveness in patients with high levels of HBV replication before LT, and the potential selection of HBsAg escape mutants. Alternative approaches have been studied, which include the use of low-dose intramuscular (IM) HBIG, subcutaneous HBIG, withdrawal of HBIG after a finite period or prophylaxis regimens without HBIG. The ability to achieve undetectable HBV DNA before LT in the majority of patients using potent antivirals allows the use of prophylaxis regimens that minimize the dose or duration of HBIG. However, a more cautious approach to a prophylaxis regimen is necessary for those patients with a high risk of HBV recurrence: high pretransplant HBV DNA levels, those with limited antiviral options if HBV recurrence occurs (i.e., HIV or HDV coinfection, preexisting antiviral drug resistance), those with a high risk of HCC recurrence, and those with a risk of noncompliance to antiviral therapy [373].

Combination prophylaxis with low-dose IM HBIG (400–800 IU IM) plus lamivudine decreases costs by more than 90 % compared to an IV regimen, with a recurrence rate as low as 4 % at 4 years [374]. Subcutaneous regimens of HBIG administered 6 months after LT have also been shown to be effectivel, with some advantage in tolerability and the possibility of self-administration by patients at home [375]. In one study on 183 patients receiving combination prophylaxis with antiviral therapy (mostly LAM monotherapy) plus HBIG given either IV high-dose (10,000 IU monthly), IV low-dose (3000–6000 IU monthly), IM low-dose (1000–1500 IU every 1–2 months), or for a finite duration (median duration 12 months). Cumulative rates of HBV recurrence at 1, 3, and 5 years were 3, 7, and 9 %, respectively. Multivariate analysis showed that positivity for HBeAg and high viral load at transplant, but not the post transplant HBIG regimen, were associated with HBV recurrence [376]. Also, the combination of HBIG and a newer nucleos(t)ide analogue (tenofovir or entecavir) was shown to be superior to the combination of HBIG and LAM in reducing the risk of HBV recurrence in one systematic review (1 vs. 6.1 %, *p* = 0.0004) [371].

Indefinite combination therapy with HBIG plus a nucleos(t)ide analogue may not be required in all liver transplant recipients. Strategy of HBIG withdrawal after a defined period of combination prophylaxis has been studied. In a study of 29 patients, high-dose HBIG and LAM were used in the first month, after which the patients were randomized to receive either LAM monotherapy or LAM plus IMHBIG at 2000 IU monthly [377]. None of the patients developed HBV recurrence during the first 18 months, but later recurrences developed in four patients after 5 years of follow-up, which was related to poor LAM compliance [378]. An alternative approach is to switch after HBIG withdrawal to a combination of LAM/ADV [379] or a combination of emtricitabine/TDV [380] or entecavir [381].

##### HBIG-free prophylactic regimens

LAM, when used as a prophylactic monotherapy (started before transplantation and continued after transplantation without HBIG), showed a 10 % recurrence rate at 1 year, but 22–41 % at 3 years after LT, due to the emergence of escape mutations in the YMDD motif of the polymerase gene [382]. Recurrence was observed mainly in patients with a high level of HBV replication prior to drug exposure [382]. In a study on 61 LAM-resistant patients treated with ADV on the wait-list who underwent LT (40 % of these patients received ADV plus/minus LAM prophylaxis without HBIG), no patient had recurrent HBV infection [383]. In another study on use of a combination prophylaxis using LAM and ADV without BIG in 18 patients who had HBV DNA below 3 log 10 IU/ml before LT, no cases of HBV recurrence were observed after a median follow-up of 22 months [384].

The availability of more potent antivirals with a higher barrier to resistance could increase the proportion of patients with undetectable HBV DNA before transplantation and decrease the risk of recurrent disease after transplantation. In a study investigating the efficacy of ETV as monoprophylaxis in 80 patients, there were no episodes of HBV flares or graft loss secondary to recurrent HBV infection. A total of 18 patients (22.5 %) had persistent HBsAg positivity after transplant without seroclearance (*n* = 8) or reappearance of HBsAg after initial seroclearance (*n* = 10). One of these patients had a very low HBV DNA level. The pre-LT HBsAg level was significantly higher in those who had HBV recurrence/persistence compared with those who did not [223]. A recent large long-term cohort study of 362 CHB post-LT patients receiving only NAs without HBIG showed that at year 8 after LT, 98 % had undetectable HBV DNA. Moreover, the survival was excellent at 83 % at 8 years, with no mortality related to HBV recurrence [385]. This clearly shows that HBIG-free regimen is safe and effective, and many studies have also demonstrated the efficacy of this therapeutic approach [386, 387].

However, HBIG remains part of the antiviral prophylaxis in many transplant centers. The use of HBIG is likely to result in a higher rate of HBsAg negativity due to the fact that the passive anti-HBs antibodies will bind with HBsAg, leading to a further reduction in detection rate when compared with HBIG-free protocols. HBV DNA persists in serum, liver, or peripheral blood mononuclear cells even 10 years after LT in a proportion of HBV transplanted patients who are HBsAg-negative. These reservoirs may serve as a source of HBV reinfection in the future, supporting the use of long-term prophylactic therapy in most patients [388, 389]. Therefore, life-long antiviral therapy is currently the standard of care after LT for CHB. In the early post transplant period, some studies reported that a high IV HBIG dosage (≥10,000 IU/day) versus a low HBIG dosage (<10,000 IU/day) was associated with a lower frequency of HBV recurrence [369]. Patients with undetectable HBV DNA levels at the time of transplant can be considered for HBIG free regimens by using high potency NAs [tenofovir or entecavir]. However, HBIG free prophylaxis should not be used for those patients with high pretransplant HBV DNA levels, those with limited antiviral options if HBV recurrence occurs (i.e., HIV or HDV coinfection, pre-existing drug resistance, or intolerance), those with a HCC at LT, and those with a risk of noncompliance to antiviral therapy [373]. Among them, HBIG withdrawal may be considered if high potency NAs are used. The timing of HBIG withdrawal is still controversial; however, 1-year post-transplantation seems to be safe and feasible [379, 390]. A recent study from India included 176 patients (at least >12 months follow-up) with HBV cirrhosis/HCC who received secondary prophylaxis with indefinite entecavir/tenofovir after living-donor LT. All patients received 10,000 IU intravenous HBIG in anhepatic phase followed by 600–1000 IU intramuscularly daily for 7 days, weekly for 3 weeks, and then monthly, to keep antiHBs levels >100 mIU/ml for 1 year. Thirty-five patients (19.8 %) had HBV DNA >2000 IU/ml before LT. After LT, patients received entecavir (*n* = 126, 71.5 %), tenofovir (*n* = 20, 11.3 %), or a combination of entecavir and tenofovir (*n* = 30, 17 % for 3 months, followed by entecavir alone). During follow-up of 43 (12–117) months, two patients (including one with non-compliance) had HBV recurrence [391].

**Fig. 6:**
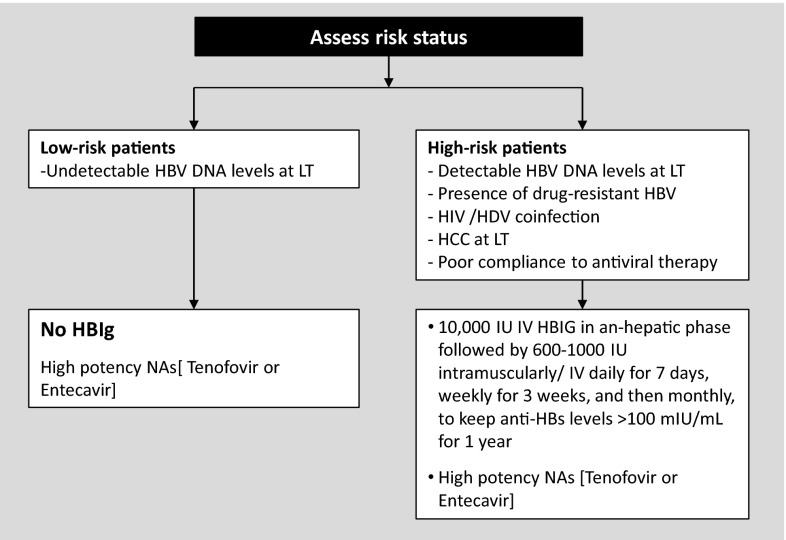
Prophylaxis for prevention of HBV graft recurrence following LT
  3.12.4 Among high-risk patients (detectable HBV DNA levels at LT, presence of drug-resistant HBV, HIV or HDV coinfection, HCC at LT or poor compliance to antiviral therapy) 10,000 IU IV HBIG in anhepatic phase should be given, followed by 600–1000 IU intramuscularly/IV daily for 7 days, then weekly for 3 weeks, and then monthly, to keep antiHBs levels >100 mIU/ml for 1 year. After 1 year, HBIg may be discontinued. High potency NAs (entecavir or tenofovir) should be continued simultaneously.

3.12 Recommendations: prevention and treatment of recurrent hepatitis B after liver transplantation
  3.12.1 Antivirals (tenofovir or entecavir) should be used before transplantation to achieve undetectable HBV DNA levels to reduce the risk of HBV recurrence (A1).
  3.12.2 A lifelong prophylactic therapy is needed (A1).
  3.12.3 Among low risk patients (i.e., with undetectable HBV DNA levels at the time of transplant), HBIg free regimens can be used. High potency NAs (entecavir or tenofovir) should be used for life (B1) (Fig. 6).

### 3.13 Treatment of chronic HBV infection in special patient groups

#### 3.13.1 Coinfection with HBV and HIV

Approximately 15–25 % of the HIV infected population in Asia and Africa has concurrent chronic HBV infection, with coinfection more common in areas of high prevalence for both viruses [392] and rates approaching 25 % in countries where the viruses are highly endemic [393]. In areas where HBV is less endemic (North America, Europe, and Australia), the overall prevalence of chronic HBV infection among HIV-infected persons is estimated to be 6–14 % [394–396].

A persistent state of immune activation in patients with chronic HBV infection could upregulate HIV replication. Early prospective cohort studies of HIV/HBV-coinfected patients revealed a 3.6-fold–6.8-fold relative risk of progression to AIDS compared to those without coinfection [397, 398]. However, other reports failed to confirm these results [399]. This discrepancy was likely related to the duration of HIV infection. To minimize the influence of duration of HIV infection, a prospective observational cohort of adult patients with primary HIV infection (seroconversion window ≤6 months) has shown that HBV coinfection (adjusted hazards ratio 3.46; 95 % CI 1.16–10.32) was an independent predictor of immunological progression that was defined as the occurrence of a CD4 cell count <350 cells/μl 3 months or more after diagnosis of primary HIV infection [400]. In another study examining the interactions of HBV and HIV using the composite endpoint of AIDS defining illnesses and death among HIV-infected individuals who had a seroconversion window of ≤3 years in a large cohort, it was found that the hazards ratio for an AIDS or death event was almost double (adjusted hazards ratio 1.80; 95 % CI 1.20–2.69) for those with HBV coinfection [401]. In the Swiss HIV Cohort Study, patients who tested positive for HBsAg had significantly impaired CD4 recovery during the first 3 years of HAART, despite similar virological effectiveness of antiretroviral therapy compared to patients without HBV infection [504 cells/μl (95 % CI 496–511) vs. 449 cells/μl (95 % CI 428–469)] [402].

Compared to HIV-uninfected subjects, patients with HIV infection have a higher risk of chronicity after acute HBV infection [403]. Clinical observational studies have demonstrated that HIV/HBV-coinfected patients may have faster progression of hepatic fibrosis and a higher risk of cirrhosis, end-stage liver disease, and HCC than HBV-monoinfected patients [395, 404]. Similarly, compared with HIV-monoinfected patients, those with HIV/HBV coinfection, especially HBV genotype B, had a higher risk of acute hepatitis, hepatic decompensation, and liver-related mortality [405]. Superinfection or coinfection with hepatitis D virus may further exacerbate the complications in patients with HIV/HBV coinfection [406].

Treatment of HIV may lead to flares of hepatitis B due to immune reconstitution, but the risk of developing cirrhosis is negligible in HBV/HIV coinfected patients on long-term tenofovir combined with emtricitabine or lamivudine therapy [407].

**Fig. 7:**
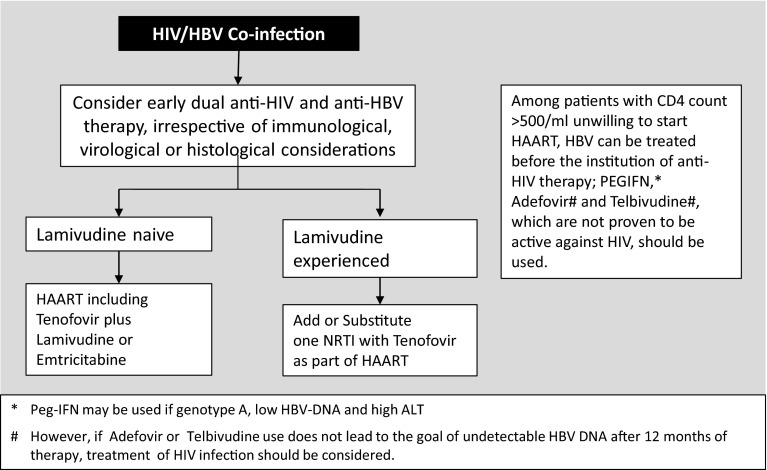
Treatment of CHB infection in HIV infected individuals

Given the faster progression of liver disease in HIV–HBV coinfected patients, there is a strong rationale for early dual anti-HIV and anti-HBV therapy, irrespective of immunological, virological or histological considerations [408]. Most coinfected patients should be simultaneously treated for both HIV and HBV de novo [409]. Lamivudine (LAM), emtricitabine (FTC) and and tenofovir (TDF) have both anti-HBV and anti-HIV activities. For most patients, the best option is triple combination of antiretrovirals, including two reverse transcriptase inhibitors with anti-HBV activity. Tenofovir combined with emtricitabine or lamivudine plus a third agent active against HIV are indicated [409, 410] (Fig. 7).

Other NAs, such as adefovir (ADV) or telbivudine (LdT) therapy, do not fit in the HIV setting due to the lack of or residual activity of these molecules against HIV and their relatively weak activity against HBV. Treatment with entecavir (ETV) may be needed in case TDF cannot be used, mostly due to kidney toxicity. Because ETV displays weak activity against HIV and may select for resistance mutations, it should be administered only in the context of a fully suppressive HIV treatment [411].

Lamivudine, entecavir and tenofovir have activity against both HIV and HBV, and are contraindicated as single agents for hepatitis B in coinfected patients because of the risk of HIV resistance. Thus, all HBsAg-positive patients should be screened for HIV before these drugs are used in the treatment of HBV infection.

Among patients with CD4 count >500/ml who are unwilling to start HAART, HBV can be treated before the institution of anti-HIV therapy; PegIFN, adefovir and telbivudine, which are not proven to be active against HIV, should be preferred [409]. Peginterferon (Peg-IFN) alpha could be considered as therapy for CHB in coinfected patients in very specific situations, such as in patients unwilling to start HAART who have normal CD4 counts >500, HBeAg(+), low HBV-DNA, elevated ALT, and lack of decompensated cirrhosis. However, if any of these two NAs (adefovir and telbivudine) with a low barrier to resistance do not reach the goal of undetectable HBV DNA after 12 months of therapy, treatment of HIV infection should be envisaged.

Oral anti-HBV drugs may select changes at the HBV polymerase, leading to loss of susceptibility to the corresponding drug and cross-resistance to other antivirals. Changes in M204 I or V are usually responsible for LAM, FTC, and LdT resistance, whereas more changes (L180M plus M204V plus T250) are usually needed for ETV resistance. Accordingly, cross-resistance is almost universal with LAM, FTC, LdT, and to a lesser extent, with ETV. There is some cross-resistance to ADV in the presence of A181S plus M204 I mutations in patients who have failed LAM therapy. No mutations have been uniformly associated with significant loss of susceptibility to TDF in vivo, although anecdotal reports have pointed out that A194T in the context of LAM resistance mutations might account for TDF resistance in HBV [412].

Resistance to LAM in HBV is more common and develops more quickly in HIV-HBV coinfected patients [413]. Selection of LAM resistance in CHB is associated with poor outcomes, including the occurrence of liver enzyme flares, which occasionally may be life-threatening, and preclude the success of rescue antiviral interventions due to cross-resistance with other antivirals. Additionally, because of overlapping polymerase and envelope genes in the HBV genome, LAM resistance mutations may result in changes in the HBsAg, causing diminished HBs antigen–antibody binding. This may translate into failure in diagnostic tests, vaccine escape, or both [414]. Transmission of drug-resistant HBV strains has also been reported [415].

HIV-infected adults without protective HBsAb titers should be vaccinated. The response rate and durability of the vaccine are poorer in HIV infected persons compared with HIV-negative persons, and they are influenced by both CD4 counts and plasma HIV-RNA levels [416, 417]. Accordingly, in patients with low CD4 counts (<200 cells/ml) and uncontrolled HIV replication, the success of HBV immunization is low. In these individuals, previous antiretroviral therapy for at least 6 months may increase HBV vaccine response rates. An initial conventional HBV vaccination schedule should be used; in the case of lack of achievement of protective anti-HBs titers (>10 mIU/ml) revaccination using double-dose and/or 3–4 injections (months 0, 1, 6, and 12) is recommended [418]. Some protection from HBV vaccine may be expected even in the case of anti-HBs titers dropping to <10 mIU/ml.

3.13.1 Recommendations: coinfection with HBV and HIV
  3.13.1.1 In HIV/HBV-coinfected patients, HBV coinfection accelerates immunological and clinical progression of HIV infection and increases the risk of hepatotoxicity when combination antiretroviral therapy is initiated, while HIV infection increases the risk of hepatitis events, cirrhosis, and end-stage liver disease related to chronic HBV infection (A1).
  3.13.1.2 Given the faster progression of liver disease in HIV-HBV coinfected patients, early dual anti-HIV and anti-HBV therapy should be considered, irrespective of immunological, virological or histological considerations (B1).
  3.13.1.3 Tenofovir combined with emtricitabine or lamivudine plus a third agent active against HIV should be used (A1).
  3.13.1.4 Peg-IFN can be used in a highly selected group of coinfected patients (B1) (Fig. 5).
  3.13.1.5 Lamivudine, entecavir and tenofovir have activity against both HIV and HBV and are contraindicated as single agents for hepatitis B in coinfected patients because of the risk of HIV resistance (A1). Thus, all HBsAg-positive patients should be screened for HIV before these drugs are used in the treatment of HBV infection (A1).
  3.13.1.6 Adefovir and telbivudine should not be used in coinfected patients (A1).
  3.13.1.7 HIV-infected adults without protective HBsAb titers should be vaccinated (A1).
  3.13.1.8 In HBV-HIV coinfected patients, an initial conventional HBV vaccination schedule should be used; in the case of lack of achievement of protective anti-HBs titers (>10 mIU/ml), revaccination using double-dose and/or three to four injections (months 0, 1, 6, and 12) is recommended (B1).

#### 3.13.2 Coinfection with HBV and HCV

 Most patients with chronic hepatitis C have a hepatitis C virus (HCV) monoinfection. However, in areas where the HBV is endemic, a substantial proportion of the patients are coinfected with hepatitis C and B [419]. If the prevalence of anti-HCV positivity worldwide is approximately 1–4 % in the general population, the number of individuals with HCV/HBV coinfection among the 320 million chronic HBV positive subjects would be approximately 3.2–12.8 million. Moreover, HCV/HBV coinfections can also be found in people at risk of parenteral hepatotropic viral transmissions, such as people who use intravenous drugs, patients with thalassemia, and patients with hemophilia.

In patients with dual chronic hepatitis B and C, the disease outcomes, including the development of liver cirrhosis (LC) and HCC, are generally more severe than those in patients with either hepatitis B or hepatitis C [420, 421]. In addition to cross-sectional data, a long-term community-based study finding supported the effect of HCV/HBV coinfection on the cumulative incidences of HCC [422]. Therefore, patients dually infected with hepatitis C and B need attention and require effective antiviral treatments.

##### Treatment goals and strategies

The primary goal of the treatment of HCV and HBV coinfection is to eliminate or permanently suppress both viruses [419]. Simultaneously, the long-term goal is to reduce or terminate hepatic necroinflammation, prevent progression to cirrhosis and the development of HCC, and ultimately prolong the survival of patients.

**Fig. 8:**
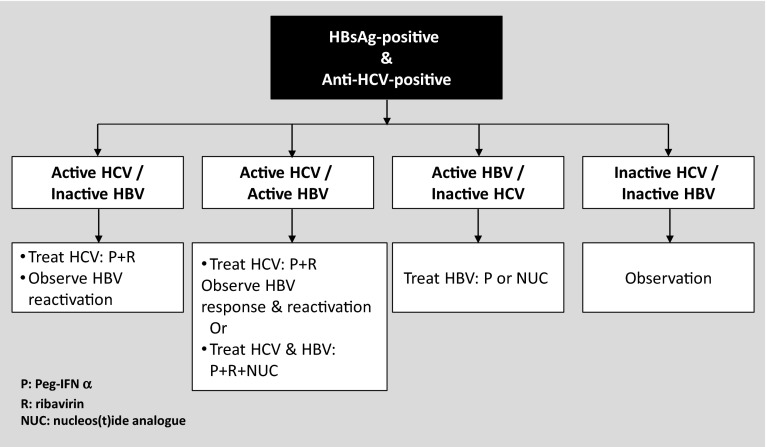
Treatment of HBV–HCV coinfected patients

These goals can be achieved by eradicating both viruses after providing an effective antiviral therapy for dually infected patients. Accumulating data exist to reach firm conclusions on the management of patients with HCV coinfection. It is generally agreed that the dominant virus should be identified before designing a therapeutic strategy (Fig. 8) [423]. HBV and HCV replicate in the same hepatocyte without interference [424]. A proportion of coinfected patients may have fluctuating serum HBV DNA levels, thus indicating the need for longitudinal evaluation of viral loads before starting any antiviral therapy, in order to clarify the respective pathogenic role of each virus [423]. HBV DNA levels are often low or undetectable and HCV is usually responsible for the activity of chronic hepatitis in most patients. If HBV is dominant, treatment should be aimed toward this virus. If HCV is dominant, Peg-IFN therapy in combination with ribavirin can achieve a sustained HCV clearance rate comparable to that in HCV mono-infection [425–428]. This has been demonstrated in an open-label, comparative, multicenter study involving 321 Taiwanese patients with active HCV infection, in which patients with HCV genotype 1 infection received Peg-IFN alfa 2a 180 µg weekly and ribavirin (1000–1200 mg) daily for 48 weeks [426]. Patients with HCV genotypes 2 or 3 received Peg-IFN alfa 2a 180 µg weekly and ribavirin 800 mg daily for 24 weeks. The sustained virological response in HCV genotype 1-infected patients was comparable between 161 HBV/HCV patients and 160 HCV mono-infection patients (72.2 vs. 77.3 %). For patients with HCV genotype 2/3 infections, the sustained virological response values were 82.8 and 84.0 %, respectively. The HCV sustained virological response (SVR) was durable in approximately 97 % of the patients during a 5-year post-treatment follow-up [427]. Furthermore, approximately 30 % of dually infected patients lost HBsAg within 5 years after the start of Peg-IFN-based therapy. The benefit of anti-HCV therapy in dually infected patients was further confirmed in another large population-based survey in Taiwan [429]. Compared with the patients in an untreated dually infected cohort, the risk of developing HCC, all-cause mortality, and liver-related mortality decreased by 35, 62, and 59 %, respectively, in patients who received active anti-HCV therapy.

3.13.2 Recommendations: coinfection with HBV and HCV
  3.13.2.1 It is important to determine the viral loads of individual viral infections and which virus is dominant before designing the treatment strategy, and then to treat the patients accordingly (B1) (Fig. 6).
  3.13.2.2 In HBV–HCV coinfected patients who are HCV viremic, antiviral treatment may be selected using the same criteria as for those patients with HCV mono-infection (A1).

#### 3.13.3 Coinfection with HBV and HDV

Although HDV can only infect HBsAg positive patients and HBV vaccine has been available for a long time, the prevalence of HDV has not shown a significant decline. Recent studies also confirm that even in countries like United States, Australia and some European countries, the prevalence of HDV is showing an increasing trend [430].

In the coinfected host, it is generally HDV which is the dominant virus because it suppresses HBV through replication, but can cause severe liver injury that may result in fulminant hepatic failure and rapid progression to cirrhosis and hepatic decompensation, as well as an increased risk of liver cancer [431]. Chronic infection after acute HBV-HDV hepatitis is less common, while chronic delta hepatitis develops in 70–90 % of patients with HDV superinfection [430]. Active coinfection with HDV is confirmed by detectable HDV RNA, immuno-histochemical staining for HDV antigen, or IgM anti-HDV [432]. However, diagnosis of active HDV infection may be difficult, as HDV RNA assays are not standardized and HDV antigen and IgM anti-HDV assays are not widely available.

Peg-IFN is effective against HDV. The efficacy of Peg-IFN therapy can be assessed during treatment (after 3–6 months) by measuring HDV RNA levels. Weekly injection of pegylated interferon is currently used for 12–18 months [433]. More than 1 year of therapy may be necessary, as there may be some benefit from treatment prolongation [434]. However, the optimal duration of therapy is not well defined [432]. So long as the hepatitis B surface antigen stays positive, HDV patients remain infective even if the HBV or HDV viral titers are low or undetected.

Around 25–40 % of treated patients have a sustained off-treatment virological response with undetectable HDV RNA and accompanying improvement in histology, while some also lose HBsAg [430, 432].

Although late relapses have been documented, in a study performed by Hedrich and colleagues in patients who were HDV RNA negative 6 months after pegylated interferon treatment, pegylated interferon alfa 2a treatment was given for 48 weeks with or without adefovir and resulted in 28 % of the patients having undetectable HDV RNA 6 months post-treatment [435]. In long-term follow-up of patients for approximately 4 years, a significant number of patients were tested HDV RNA positive at least once during further follow-up, and it was also concluded by the investigators to closely monitor patients post-Peg interferon therapy, even those who are HDV RNA negative 6 months after therapy with interferon alfa 2a therapy.

When standard interferon was used at nine million units compared to no treatment or low dose at three million units given three times a week for 48 weeks, 50 % of the high-dose group had a complete biochemical response defined by normalization of ALT, in addition to virological response negative HDV RNA at the end of the treatment, compared to no complete responses in any of those in the low-dose or no treatment group. The long term follow-up up to 12 years demonstrated significantly improved survival and liver histology for the high dose treatment group, although most of them relapsed after clearance of HDV RNA [436].

Although no head-to-head comparison trials have been carried out, two major reviews have not been able to definitely show that either type of interferon therapy is superior to the other. However, one recent systematic review of randomized trials found that 1 year of high dose interferon alfa monotherapy achieved higher levels of undetectable HDV RNA and normalization of ALT at the end of treatment when compared with pegylated interferon alfa 2a monotherapy. However levels of HDV RNA suppression 24 weeks after the end of therapy were not significantly different [437]. A systematic review by Alavian and colleagues comparing standard and pegylated interferon alfa found sustained virological response rates in 19 and 29 % of patients, respectively [438]. In a study from Turkey using entecavir for chronic hepatitis D, after 1 year of entecavir treatment, it was found to be ineffective in CHD. It was also concluded from the study that any beneficial effect of nucleoside–nucleotide analogue treatment may necessitate prolonged treatment [439]. In a recent study from Pakistan, sustained virological response, which was defined as negative HDVRNA at 24 weeks post-treatment, was seen in 23.1 % for virological and biochemical responses and in only 12.5 % as a combined response [440]. A Cochrane review concluded that interferon alfa does not seem to cure Hepatitis D in most patients. It was also concluded from this review that more randomized trials with large sample sizes and less risk of bias were needed before interferon can be recommended or refuted [441]. In a recent study from Germany by Nikongolo [442], it was suggested that HBV and HDV entry via sodium taurocholate co transporting polypeptide is inhibited by cyclosporine A. In the future, this drug may help reduce the incidence of HBV and HDV after more studies demonstrate its usefulness and where it would actually fit in the management of HBV and HDV coinfection. Myrcludex-B, a myristoylated a preS/2–48my2 peptide, has been shown to limit the establishment of HDV infection in vivo and delayed the increase in HBV viremia. The real role of its use is yet to be determined in HBV-HDV management [443].

Lamivudine, adefovir and entecavir have been found to be ineffective in the management of Hepatitis D alone or in combination with interferon; however, Wedemeyer, in his study using pegylated interferon and adefovir, showed significant decline in HBsAg titers using adefovir [444], which could be significant as a predictor for successful treatment of HBeAg-positive CHB [280]. Case reports have appeared in which successful treatment of HBV and HDV have been reported using pegylated interferon and entecavir [445] and pegylated interferon and tenofovir and emtricitabine [446]. Thus, NAs treatment might be considered in some patients who have active HBV replication with persistent or fluctuating serum HBV DNA levels above 2000 IU/ml [447].

3.13.3 Recommendations: coinfection with HBV and HDV
  3.13.3.1 In patients with coinfection of HBV and HDV, it is important to determine which virus is dominant and the patient should be treated accordingly with pegylated interferon alfa for 12–18 months. Patients should be monitored for 6 months post-treatment and beyond (A1).

#### 3.13.4 Health care workers

HBV can survive in dried blood outside the body for up to 7 days, and is significantly more infectious than either hepatitis C or HIV, with a reported transmission rate of up to 30 % from needlestick injuries. This rate seems to correlate with serum HBV DNA concentrations. The concentration of HBV varies across body fluids, with blood, serum and wound exudates carrying the highest concentrations; semen, vaginal fluid and saliva carrying moderate concentrations; and urine, feces, sweat and breast milk containing the lowest concentrations, which translates into the lowest risk of HBV transmission. Percutaneous injuries sustained by health-care workers during certain surgical, obstetrical, and dental procedures provide a potential route of HBV transmission to patients as well as to heath care workers (HCWs). Therefore, it is important to prevent operator injuries and blood exposures during exposure-prone surgical, obstetrical, and dental procedures.

Chronic HBV infection in itself should not preclude the practice or study of medicine, surgery, dentistry, or allied health professions. Standard precautions should be adhered to rigorously in all health-care settings for the protection of both patient and provider [448].

HCWs and students of surgery, dentistry, medicine, or allied health fields should be screened for HBV infection. Testing should include a serological assay for HBsAg, ant-HBs and Total anti-HBc. All noninfected health-care providers and students should receive hepatitis B vaccine. Vaccination (three-dose series) should be followed by assessment of hepatitis B surface antibody to determine vaccination immunogenicity, and providers who do not have protective concentration of anti-HBs (>10 mIU/ml) should undergo, revaccination [448].

Exposure of a HCW to the blood of an HBV-infected patient in the performance of any procedure, should be handled with standard post-exposure prophylaxis. Exposure of a patient to the blood of an HBV-infected health-care provider, in the performance of any procedure, should be handled with post-exposure prophylaxis and testing of the patient in a manner similar to the reverse situation (i.e., prophylaxis for providers exposed to the blood of an HBV-infected patient) [449].

##### Transmission of HBV by HCWs to patients

In the health care setting, transmission may occur via several routes, but the most frequent route leading to establishment of HBV infection is through needlestick injury. Invasive surgical procedures are another route of HBV transmission; in fact, surgeons represent the largest group of HCWs involved in provider-to-patient HBV transmission [450].

It is the regular performance of an exposure-prone procedure (EPP) that is mainly of concern. EPPs are defined as procedures in which there is a risk that injury to the physician may result in the exposure of the patient’s open tissues to the blood of the physician. Any type of invasive surgery is, thus, an EPP, wherein the affected physician’s gloved hand is in constant contact with sharp instruments, needle tips or sharp tissues (spicules of bone or teeth) inside a patient’s open body cavity. Surgery performed within a confined anatomical space, where the hands or fingertips may not always be completely visible, also carries an elevated risk of transmission, given the paucity of surgical precision and control in this context. A procedure is considered to be non-exposure-prone (NEPP) when the hands and fingertips of the physician are visible and outside the patient’s body throughout, even when there is handling of sharp instruments. A NEPP can become an EPP if a patient is uncooperative [451].

Retrospective studies have evaluated the rate of HBV transmission from affected physicians through blood contact during specific types of EPPs. Percutaneous injuries have been reported to occur in 6.9 % of operations, and in 32 % of these instances, the instigating sharp instrument touches the patient wound once again [452]. The risk of HBV transmission is not negligible; the rate in cardiothoracic surgery is reported to be 6–13 % [453, 454], up to 9 % in gynecological surgery [455, 456], and 2 % in general surgery [457, 458]. The proportion of patients infected with HBV secondary to transmission from an infected HCW is between 0.5 and 13.1 % [459].

CDC has classified patient care procedures into two categories (Table 11).

**Table 11:** Classification of patient care procedures

Procedures known or likely to pose an increased risk of percutaneous injury to a HCW that have resulted in provider-to-patient transmission of hepatitis B virus (HBV)	Category II—all other invasive and noninvasive procedures
These procedures are limited to major abdominal, cardiothoracic, and orthopedic surgery, repair of major traumatic injuries, abdominal and vaginal hysterectomy, caesarean section, vaginal deliveries, and major oral or maxillofacial surgery (e.g., fracture reductions). Techniques that have been demonstrated to increase the risk for health-care provider percutaneous injury and provider digital palpation of a needle tip in a body cavity and/or the simultaneous presence of a health care provider’s fingers and a needle or other sharp instrument or object (e.g., bone spicule) in a poorly visualized or highly confined anatomic site	These and similar procedures are not included in Category I as they pose low or no risk for percutaneous injury to a health-care provider, or, if a percutaneous injury occurs, it usually happens via provider-to-patient blood exposure. These include surgical and obstetrical/gynecological procedures that do not involve the techniques listed for Category I; and provider’s hands are outside a body cavity (e.g., phlebotomy, placing and maintaining peripheral and central intravascular lines, administering medication by injection, performing needle biopsies, or lumbar puncture)
Category I procedures, especially those that have been implicated in HBV transmission, are not ordinarily performed by students fulfilling the essential functions of a medical or dental school education rectal examination; and procedures that involve external physical touch (e.g., general physical or eye examinations or blood pressure checks)	Dental procedures other than major oral or maxillofacial surgery Insertion of tubes (e.g., nasogastric, endotracheal, rectal, or urinary catheters) Endoscopic or bronchoscopic procedures Internal examination with a gloved hand that does not involve the use of sharp devices (e.g., vaginal, oral)

Most effective transmissions have occurred when the HCW carried HBV DNA >1.9 × 105 IU/ml (10^6^ copies/ml, with conversion factor of 5.26 copies/IU). Establishing a threshold for the limitation of EPPs would have to account for a 3 log10 safety margin to account for assay variability [452]. HCWs who are HBsAg positive should be tested for HBeAg and anti-HBe and for HBV viremia.

HCWs, and medical and dental students who are HBsAg-positive, who do not perform exposure-prone procedures but who practice non- or minimally invasive procedures (Category II, table) should not be subject to any restrictions of their activities or study. They do not need to achieve low or undetectable levels of circulating HBV DNA, hepatitis e-antigen negativity, or have review and oversight by an expert review panel, as recommended for those performing exposure-prone procedures. However, they should receive medical care for their condition by appropriate clinicians [460].

HCWs who perform exposure-prone procedures, i.e., those listed under Category I activities (Table 11), should be guided by an institutional expert review panel regarding the procedures that they can perform and prospective oversight of their practice [461]. Confidentiality of the health-care provider’s or student’s HBV serological status should be maintained. HBV-infected HCWs can conduct exposure-prone procedures if a low or undetectable HBV viral load is documented by regular testing at least every 6 months unless higher levels require more frequent testing; for example, as drug therapy is added or modified, testing is repeated to determine if elevations above a threshold are transient [460]. An HBV DNA level of 1000 IU/ml or its equivalent is an appropriate threshold to adopt [460]. Spontaneous fluctuations of HBV DNA levels and treatment failures might both present as higher-than-threshold (1000 IU/ml) values. This will require the HBV-infected HCW to abstain from performing exposure-prone procedures, while subsequent retesting occurs, and if needed, modifications or additions to the health-care provider’s drug therapy and other reasonable steps are taken [460].

Hospitals, medical and dental schools, and other institutions should have written policies and procedures for the identification and management of HBV-infected health-care providers, students, and school applicants.

##### Treatment of HCWs for reduction of Infectivity

Healthcare workers also need special attention regarding starting antiviral therapy, as they may require antiviral therapy even if they do not fulfill the typical indications for treatment, to reduce direct transmission during exposure-prone procedures to patients.

Published evidence for the efficacy of antiviral therapy on the transmission rate to patients is limited. One Dutch study reported reduction of viremia to <1000 copies/ml in 18 HCPs with either interferon-α or various NAs [462].

DNA levels <1000 IU/ml are reached more often and much faster in HBeAg-negative chronic HBV-infected subjects, because they have lower baseline levels of viremia and possibly a higher turnover of HBV-containing hepatocytes, as compared to HBeAg-positive subjects. The immunotolerant HBeAg-positive subjects typically have baseline viremia levels >10^8^ IU/ml. The mean reduction of viremia obtained in HBeAg-positive patients with entecavir within 1 year was reported to be 6.9 log10 copies/ml [463]. One study on tenofovir therapy showed that the mean value in patients with moderately high viremia fell from 7.3 to 3.7 log10 copies/ml within approximately 12 weeks, while patients with >9.0 log10 baseline level needed roughly 52 weeks [196]. In a direct comparison of tenofovir and entecavir, decreases of −4.0 or −4.5 log10 units/ml, respectively, were found after 3 months of therapy [464]. Thus, viremia can be suppressed to acceptable levels in the majority HCWs by 3 months of entecavir or tenofovir therapy. HCWs with very high viremia >10^8^ IU/ml may need longer therapy, but most of them will reach acceptable or even undetectable levels within 1 year [465].

3.13.4 Recommendations (health care workers)
  3.13.4.1 Chronic HBV infection in itself should not preclude anyone from the practice or study of medicine, surgery, dentistry, or allied health professions (A1). Such HCWs should not be isolated or discriminated, but should be encouraged to be investigated and treated (A1).
  3.13.4.2 HCWs and students of surgery, dentistry, medicine, or allied health fields should be screened for HBV infection. Testing should include a serological assay for HBsAg, anti-HBs and total anti-HBc (A1).
  3.13.4.3 All non-infected health-care providers and students should receive hepatitis B vaccine and their immunization status be confirmed (A1).
  3.13.4.4 Standard precautions should be adhered to rigorously in all health-care settings for the protection of the patient and the provider (A1).
  3.13.4.5 HCWs who perform exposure-prone procedures, i.e., those listed under Category I activities (Table 11), should be guided by an institutional expert review panel regarding the procedures that they can perform and prospective oversight of their practice (B1). The status of the individual could vary depending on the response to therapy.
  3.13.4.6 HBV-infected HCWs can conduct exposure-prone procedures if a low (<1000 IU/ml) or undetectable HBV viral load is documented by regular testing at least every 6 months (B1).

#### 3.13.5 Chronic HBV infection and pregnant females

When females in the childbearing age require antiviral therapy, the issue of pregnancy must be discussed before starting treatment. If pregnancy is planned in approaching years, IFN-based therapy is preferable for its finite duration of treatment. Pregnancy is discouraged during IFN therapy because of IFN's anti-proliferative effect. Contraception is suggested during IFN treatment. In pregnant females with chronic HBV infection who need antiviral therapy, or in females who have unexpected pregnancy during antiviral treatment, the treatment plan should be fully discussed, considering risks and benefits for the mother and fetus on issues regarding risks of maternal disease progression, maternal ALT flares, fetal development, vertical transmission of HBV, long-term plan for treatment and next pregnancy [466, 467]. Among the currently available NAs, LdT and TDF are classified as category B drugs (no risk in animal studies, but unknown in humans), whereas LAM, ADV, and ETV are classified as category C drugs (teratogenic in animals, but unknown in humans) by the US FDA. Category B NAs (LdT and TDF) may be considered for mothers indicated for antiviral treatment during the first through third trimester of pregnancy. TDF has more safety data in HIV-positive females, and the least chance of viral resistance. Safety data from the Antiretroviral Pregnancy Registry has demonstrated no increased rates of birth defects (2.8 % 46/1982) with TDF exposure during the first trimester [468]. Despite postnatal active/passive immunization of the newborns, mother-to-infant transmission of HBV still occurs; major risk factors are maternal HBeAg and high viral load [469, 470]. For prevention of mother-to-infant transmission that occurrs during theperinatal period, short-term maternal NAs used in mothers of stable liver disease, starting from the second or third trimester, has been documented to reduce maternal viral load and decrease perinatal mother-to-infant transmission. The results are based on non-randomized, open label clinical studies using either LAM, LdT or TDF [471–478]. The target population for short-term NAs treatment for pregnant females to reduce maternal HBV transmission is maternal HBV viral load above 6–7 log_10_ IU/ml [469]. The starting point of maternal treatment in most studies is 28–32 weeks of gestation, after careful examination to exclude maternal systemic disorder and fetal anomalies. Cessation of NAs therapy (at delivery or 4–12 weeks after delivery) is recommended in females without ALT flares and without pre-existing advanced liver fibrosis/cirrhosis. Continuation of NAs treatment after delivery may be necessary according to maternal liver disease status. Breastfeeding is not discouraged in mothers with chronic HBV infection if newborns have received appropriate postnatal immunoprophylaxis. However, breastfeeding is not generally encouraged during NAs therapy because of uncertainty of safety to newborns [479, 480]. There has been insufficient data regarding maternal ALT flare rates before and after delivery, especially after cessation of NAs postpartum. Some studies reported increased rates of ALT flares in the LdT or LAM treated group [476], and other studies using TDF reported comparable or possibly beneficial effects for the mothers [472, 481]. Several issues are still not well understood, such as the long-term safety of the mothers and child beyond 1 year post delivery, optimal large-scale screening methods, and cost-effectiveness of such prevention strategy for the population.

3.13.5 Recommendations: chronic HBV infection and pregnant female
  3.13.5.1 The issue of pregnancy and maternal–fetal–child health should be notified in chronically HBV-infected female in the childbearing age, especially when antiviral treatment is considered. The treatment plan should be fully discussed with the patient and relatives, especially regarding the risks of maternal liver disease status, fetal development, vertical transmission of HBV, long-term plan for treatment and pregnancy. Maternal HBeAg, HBV DNA status, and ALT level should be checked during pregnancy (A1).
  3.13.5.2 In pregnant females with chronic HBV infection who need antiviral therapy, tenofovir is the drug of choice for mothers indicated for antiviral treatment during the first through third trimester of pregnancy. It is a pregnancy category B drug with adequate safety data in HIV-positive females and least chance of viral resistance (B1).
  3.13.5.3 For reduction of risk of mother-to-infant transmission that occurs during perinatal period, short-term maternal NAs starting from 28 to 32 weeks of gestation is recommended using either tenofovir or telbuvidine for those mothers with HBV DNA above 6–7 log_10_ IU/ml (B2). Since, the HBV transmission could occur even with lower maternal HBV DNA levels, NAs could be administered after discussion with the patient, even in patients with lower DNA levels. The NAs could be stopped at birth and when breastfeeding starts, if there is no contraindication to stopping NAs (B2).
  3.13.5.4 Breast-feeding is discouraged during maternal NAs treatment. For those with ALT flares detected during the treatment period, continuation of antiviral treatment according to maternal liver disease status may be indicated (B2).

#### 3.13.6 Chronic HBV infection in patients with CKD, on dialysis and in renal transplant patients

Chronic HBV infection has a pivotal influence on patients with CKD undergoing haemodialysis and renal transplant with complex issues [482]. Patients with renal disease should be screened for HBV infection and though vaccine responsiveness is impaired, HBV seronegative patients should be vaccinated. In particular, renal patients under anti-HBV therapy should be followed not only for the treatment efficacy, but also the stage of liver disease and the renal disease status. Peg-IFN or NAs can be used for chronic HBV infection patients with renal dysfunction. However, NAs represent the first-line treatment option for chronic HBV-infected patients with any level of renal dysfunction and renal replacement therapy. Physicians should be aware of the necessary drug dose adjustments according to creatinine clearance as well as the potential nephrotoxicity and long-term drug efficacy.

In general, entecavir, an agent without signs of nephrotoxicity, and telbivudine, an agent with promising data for even improvement in creatinine clearance, seem to be the preferred options for NA-naive patients with any renal dysfunction, depending on the HBV viremia levels and the severity of renal dysfunction. Although, there are no definite conclusions about the risk of tenofovir-associated nephrotoxicity, most clinicians are concerned and therefore avoid using this agent in this setting. However, tenofovir remains the agent of choice for patients with renal dysfunction and prior resistance to other NAs.

HBsAg positive patients who undergo renal transplantation and receive immunosuppressive agents should receive anti-HBV prophylaxis with NAs. However, Peg-IFN should be avoided in renal transplant patients because of the risk of rejection.

3.13.6 Recommendations: chronic HBV infection in patients with CKD, on dialysis and renal transplant patients
  3.13.6.1 NAs (entecavir or telbivudine) represent the first-line treatment options for chronic HBV-infected patients with any level of renal dysfunction and renal replacement therapy. NAs should be dose adjusted based on creatinine clearance rates (A1).
  3.13.6.2 Peg-IFN should be avoided in renal transplant patients because of the risk of rejection (A1).

#### 3.13.7 Extrahepatic manifestations of chronic hepatitis B

Several extrahepatic conditions have been described during acute and chronic HBV infection. While the pathogenesis remains controversial, it is largely attributed to an immune-mediated injury of organs other than the liver. Viral antigen-induced induction and deposition of immune complexes, reaction with tissue antigens by HBV-induced auto antibodies, or a direct viral reaction may occur in extrahepatic tissues such as the skin, muscles, joints and kidneys.

##### Glomerulonephritis

The incidence of HBV-related glomerulonephritis is from 0.1 to 25 % and may present clinically in three forms, i.e., membranous, membranoproliferative, and IgA nephropathy. Membranous glomerulonephritis (MGN) is the most common type, especially in areas endemic for HBV infection, and usually presents as nephrotic syndrome, with proteinuria, edema and hypertension. Immune complexes are deposited only in the basement membrane where HBV antigens, i.e., HBsAg, may be identifiable in the glomerular capillary wall. In children, up to 60 % may experience spontaneous remission, usually associated with HBeAg seroconversion. However, in adults it can lead to chronic renal failure in up to 30–50 % of cases [483, 484]. Membranoproliferative glomerulonephritis (MPGN) is characterized by deposition of HBsAg in both the mesangial and capillary walls; HBeAg and HBcAg have also been identified in the glomeruli. In chronic HBV infection, the heightened immune response results in increased amounts of circulating immune complexes containing HBV antigens, complement components, immunoglobulins, etc., which are deposited in sites outside the liver [485]. HBV-related IgA nephropathy is a less severe form of renal disease and usually evolves with an indolent course, although an aggressive course with progression to acute renal failure has been reported. Tubulo-reticular inclusions in the endoplasmic reticulum of endothelial cells of the glomerular and peritubular capillaries have been identified on electron microscopy [486]. It has been suggested that both humoral and cellular immune injury, mediated by HBAg-HBAb immune complexes in the former and by HBV originating from renal cells in the latter, may be involved in the pathogenesis of IgA nephropathy [487]. Remission of clinical and laboratory manifestations of nephropathy with successful antiviral treatment have been demonstrated [488–491].

##### Polyarteritis nodosa

Polyarteritis nodosa (PAN) is a generalized necrotizing vasculitis, and HBV-associated PAN (HBV-PAN) represents a typical form of classic PAN. The pathogenesis HBV-PAN is largely attributed to immune-complex deposition (with antigen excess) in the vessel walls of the skin, kidneys, heart and nervous system. A recent study of 348 patients with PAN revealed that patients with HBV-PAN had more frequent peripheral neuropathy, abdominal pain, cardiomyopathy, orchitis, and hypertension compared to patients with non-HBV-related PAN. PAN is observed more frequently in European and North American patients, but rarely in Asian patients [492]. Constitutional symptoms include malaise, anorexia, weakness, fever, and weight loss. Erythematous skin lesions and palpable purpura and nodules are not uncommon. Prior reports have estimated the incidence of HBV infection in PAN patients to be between 30 and 70 %; however, in the West, these figures have declined remarkably in parallel with those of HBV infection [493]. Antiviral therapy, combined with corticosteroids and plasma exchanges, has demonstrated good efficacy in the management of HBV-PAN [494, 495].

##### Cryoglobulinemia

Patients with chronic HBV infection may present with mixed cryoglobulinemia, i.e., type II (monoclonal IgM and polyclonal IgG) and type III (polyclonal IgM and monoclonal IgG). The prevalence of HBV-associated cryoglobulinemia ranges from 0 to 15 % [496, 497]. Clinically, it may present with protracted purpura, with or without ulcerative skin lesions, arthralgia, and weakness. It may be associated also with the sicca syndrome, Raynaud phenomenon, as well as renal and neurological complications. When nephritis is present, the clinical course can rapidly be fatal. Effective treatment of the underlying chronic HBV infection with currently available nucleos(t)ide analogues generally leads to clinical and serological resolution of cryoglobulinemia [498, 499].

##### Serum sickness-like syndrome

A transient serum sickness-like “arthritis–dermatitis” syndrome occurs in approximately 10–20 % of patients during the prodrome of acute hepatitis B [500]. The pathogenesis is related also to circulating immune complexes, and during the acute phase, high concentrations of HBsAg have been detected in the synovial fluid with associated reduction in complement levels. The manifestations can range from fever, myalgia, polyarthralgia or overt arthritis with joint swelling and edema of small joints of the hands and feet, as well as large joints of the knees, ankles and wrists. The polyarthritis is characteristically asymmetrical and is often associated with erythematous skin lesions. Morning stiffness and a “gel” phenomenon are present; thus, it can be mistaken for acute rheumatoid arthritis. However, it typically disappears when jaundice sets in and leaves no demonstrable permanent joint destruction. The resolution of arthritic lesions parallels those of HBsAg clearance. This serum sickness-like syndrome ends abruptly with the onset of clinical hepatitis with few significant sequelae, and does not recur [501].

##### Dermatological manifestations

The skin rashes in patients with chronic HBV infection are usually related to immune complex deposition, neutrophilic infiltration and small vessel necrosis. They present typically as palpable purpura. Lichen planus, a chronic recurrent rash composed of small, flat-topped, polygonal bumps that may coalesce together into rough, scaly plaques on the skin and mucous membranes, has been found to be highly prevalent in Turkish patients who are seropositive with HBsAg [502]. The Gianotti-Crosti syndrome, or papular acrodermatitis of childhood, is characterized by small, flat, erythematous, papular or papulovesicular rash that occurs in the face and distal extremities of infants and young children. Other than HBV, the Epstein–Barr virus, hepatitis A virus, cytomegalovirus, coxsackie, adenovirus, enterovirus, HIV are also implicated as etiological agents. While the association of this syndrome with HBV infection was reported as early as 1976, this association remains controversial [503].

##### Guillain–Barré syndrome

Guillain–Barré syndrome (GBS) is a rare extrahepatic involvement associated with both acute and chronic HBV infection. While both HBsAg and HBV DNA have been detected in cerebrospinal fluid, it is unclear whether the virus itself or an immune-mediated assault or a vasculitis-related injury to the myelin sheath is responsible for these symptoms of the central nervous system [504]. A recent case report described a patient with GBS associated with acute hepatitis B responding to nucleoside analogue and intravenous immunoglobulin treatment [505].

3.13.7 Recommendations: extrahepatic manifestations of CHB
  3.13.7.1 Extrahepatic manifestations may be associated with both CHB infection (glomerulonephritis, polyarteritis nodosa, mixed cryoglobulinemia, and skin manifestations) and acute HBV infection (Guillain–Barré syndrome and, a serum sickness-like syndrome) (B1).
  3.13.7.2 HBsAg positive patients with extra-hepatic manifestations and active HBV replication may respond to antiviral therapy (B1).
  3.13.7.3 Peg-IFN may worsen some immune mediated extra-hepatic manifestations (B1).
  3.13.7.4 Plasmapheresis, corticosteroids or IVIG can be useful in addition to NA therapy in severe immune-mediated cases (C2).

#### 3.13.8 Patients before and/or after curative or local–regional therapy of HCC

Once HCC develops, treatment for HBV will depend on the stage of disease. Mostly patients will be on antiviral therapy, as the majority will have underlying cirrhosis.

For non-surgical patients, a high viral load prior to chemotherapy or locoregional therapy results in higher rates of severe hepatitis during chemotherapy [506]. Longer survival has been shown in patients receiving TACE with the additional of antiviral therapy [507].

For the overwhelming majority of patients with HCC, surgical removal of the tumor by resection or LT is the only curative option. HCC recurrence occurs in up to 41–50 % of patients within 2 years after resection (early recurrence) and in up to 20 % of patients more than 2 years later (late recurrence) [508]. Most early recurrence appears to reflect diffusion of primary tumors, while most late recurrence stems from de novo tumors spontaneously arising in the remnant diseased liver. Antiviral therapy is important for patients undergoing resection, as the hepatic reserves will be limited and compromised in the post-operative period. Therefore, flares of hepatitis may lead to decompensation for untreated patients [509]. Surgery and anesthesia may also impart a state of immunosuppression in the early post-operative period, thereby increasing the risk of HBV reactivation [510]. A high pre-operative viral load has been associated with worse overall and recurrence free survivals after curative resection [511]. There is also the potential increased risk of recurrent HCC due to the process of necrosis and regeneration of remaining hepatocytes, which may induce DNA mutations and instability. Upregulation of adhesion molecules on cells lining sinusoids may increase the risk of distant metastasis [512].

Viral load and hepatic inflammatory activity have been associated with late recurrences after HCC resection [513]. A cohort of 72 resected patients with HBV-relatedHCC showed that the absence of antiviral treatment was a risk in tumor recurrence [514]. An HBV DNA of >2000 IU/ml at the time of resection was a significant risk factor (RR 22.3, 95 % CI 3.3–150.5, *p* = 0.001).

Routine prophylactic NA therapy for HCC patients with HBV-DNA levels <2000 IU/ml before liver resection may also be considered. The aim is to prevent HBV reactivation after liver resection, which occurs in as many as 19 % of patients within the first 1 year, and which can severely reduce liver function and survival [515].

Since NAs cannot completely eradicate HBV, lifelong treatment should be pursued as long-term therapy may help prevent hepatitis flare-ups and inhibit hepatocarcinogenesis to the greatest extent [516].

Various studies have found that antiviral therapy decreases HCC recurrence after resection. In a nationwide cohort study from Taiwan of 4051 untreated versus 518 NA-treated CHB patients with resected HCC, even though there was a higher rate of cirrhosis in the latter (38.7 vs. 48.6 % respectively, *p* < 0.001), the risk of HCC recurrence was lower in the NA-treated patients (43.6 vs. 20.5 % respectively, *p* < 0.001) [517]. NA use was independently associated with a significantly lower HCC recurrence risk (HR 0.67, 95 % CI 0.55–0.81, *p* < 0.001). A meta-analysis also demonstrated the beneficial effects of antiviral therapy with regards to HCC recurrence (OR 0.59, 95 % CI 0.35–0.97, *p* = 0.04), and liver-related mortality (OR 0.13, 95 % CI 0.02–0.69, *p* = 0.02) [518]. Two recent meta-analyses including 20 studies demonstrated that the presence of high viral load significantly increased overall HCC recurrence risk after curative therapy, whereas antiviral therapy had potential beneficial effects in preventing recurrence [519, 520].

There is also improvement in recurrence-free survival and overall survival with NAs treatment among patients undergoing resection for HBV-related HCC. In a recent systematic review of 19 studies, the NA group (1468 patients) showed a median recurrence-free survival of 85.0 % (range 19.7–90.0 %) at 1 year, 57.0 % (range 11.4–90.0 %) at 3 years, and 54.0 % (range 42.6–81.3 %) at 5 years. These median survival rates were significantly higher than the corresponding values in the non-NA group (5541 patients): 78.0 % (range 4.5–86.6 %) at 1 year, 56.0 % (range 0–56.0 %) at 3 years, and 47.0 % (range 0–47.0 %) at 5 years (all *p* < 0.001) [521]. In the same review on 15 studies reporting overall survival, the overall median survival in the NA group (1468 patients) was 94.0 % (range 24.0–100.0 %) at 1 year, 81.0 % (range 60.0–100.0 %) at 3 years, and 73.0 % (range 59.0–89.7 %) at 5 years. These values were significantly higher than the corresponding ones for the non-NA group (5200 patients): 91.0 % (range 0–100.0 %) at 1 year, 74.0 % (range 0–85.0 %) at 3 years, and 62.0 % (range 0–70.0 %) at 5 years (all *p* < 0.001) [521].

Thus, use of antiviral therapy improves the long-term post-hepatectomy recurrence and survival in patients with HBV-related HCC. With a better liver function reserve at the time of recurrence, a greater proportion of patients in the antiviral group could receive curative treatment for recurrence [522].

Interferon treatment as tertiary prevention of HBV-related HCC recurrence remains controversial [523–525]. Use of interferon treatment in HCC patients may be complicated and even risky, as these patients are more vulnerable to the development of hepatic decompensation with life-threatening complications such as hepatic encephalopathy and ascites. In contrast, nucleos(t)ide analogues are, in general, safer and better tolerated than interferon.

HBV recurrence after liver transplantation has always been a major problem for HBV-related HCC. Pre-transplant HBV DNA level and antiviral treatment was a major risk factor associated with HBV recurrence after liver transplantation [526]. For all recipients with high load of HBV DNA, a potent, high resistance NAs should be given as early as possible before transplantation. HBIG should be given during the anhepatic phase. NAs in combination with low dose HBIG have been proved to reduce HBV recurrence after transplantation [527]. The most recent data showed entecavir or tenofovir were more effective NAs using this strategy [528] (see “3.12 Prevention and treatment of recurrent hepatitis B after liver transplantation” section).

3.13.8 Recommendations: patients before and/or after curative or local–regional therapy of HCC
  3.13.8.1 NAs treatment should be given to patients with HBV-related HCC (at least 1–2 weeks before, during and after chemotherapy, locoregional therapies, resection or LT), if they have detectable serum HBV-DNA (B1).
  3.13.8.2 Because NA therapy cannot completely eradicate HBV, lifelong treatment is needed (B2).
  3.13.8.3 HBIG should be given to recipients with high viral loads in anhepatic phase, followed by combination therapeutic modalities with NAs and low-dose HBIG after LT to prevent HBV recurrence (B1).

#### 3.13.9 Chronic HBV infection in children

The Oxford dictionary states that ‘A young human being below the age of puberty or below the legal age of majority should be regarded as ‘child’. On the other hand, The Nations Convention defines child as ‘a human being below the age of 18 years’ [529]. The UN convention has been ratified by 192 of 194 member countries. However, different countries may have different age settings for children in medical set-up. Also, the age of voting rights differ in different countries, ranging from 18 to 20 years. The definition of ‘child’ is of utmost importance for the treatment of chronic HBV-infected patients, as the pathogenesis of HBV takes critical clinical turns in ‘child’, especially around 16–18 years of age. Transmission modalities for HBV infection vary between different regions of the world. In highly endemic areas, most infections are transmitted from mother to child vertically/perinatally (mainly in Asian countries) or through horizontal transmission from child to child during early childhood (mainly in African countries) [530]. In countries of intermediate endemicity, HBV infection occurs in all age groups, whereas in areas of low endemicity, infection occurs primarily in adult life through sexual or parenteral transmission (e.g., drug use). In these countries, surgery, dental care, tattooing, and body piercing may be relevant sources of infection, while transfusion-related infections have become very rare because of improved blood screening [531]. The age at the time of HBV acquisition is the major determinant of chronicity, as about 90 % of newborns who acquire HBVperinatally develop chronic HBV infection In contrast, only 25–50 % of children who acquire the virus in the first 6 years of life and 5 % of adults become chronically infected [532].

##### Natural history of chronic HBV infection in children

The vast majority of children infected at birth are immune-tolerant with high HBV DNA levels in serum and the presence of HBeAg for years, typically into late childhood or adolescence. Generally, in this phase, despite the high-level HBV replication, the host T-cell response is suppressed, and infected hepatocytes are therefore not attacked. Alanine aminotransferase (ALT) levels are frequently normal or slightly increased, and histological changes are minimal. Transplacental transfer of maternal HBeAg can induce tolerance of helper T cells of newborns to HBeAg [309]. The affected children are usually asymptomatic and have normal growth.

The immune-active phase is characterized by elevation of aminotransferase and fluctuating serum HBV-DNA levels. This phase may lead to seroconversion. Spontaneous seroconversion rates (loss of HBeAg and development of anti-HBe) in these perinatally infected children are low, occurring in fewer than 2 %/year of children younger than 3 years and in 4–5 % of children older than 3 years [533]. These rates are much lower than those (14–16 %/year) observed in children infected horizontally after the perinatal period [534, 535].

After achievement of anti-HBe seroconversion, serum HBsAg persists, but aminotransferase levels return to normal and HBV DNA becomes very low or undetectable. This state is the low replicative phase, and in this phase, liver disease progresses very slowly. Available data on long-term follow-up of children in low replicative phase without signs of cirrhosis at the time of seroconversion have demonstrated no progression to cirrhosis over about 30 years [536, 537]. The complete resolution of HBV infection is characterized by loss of HBsAg and appearance of anti-HBs. This spontaneous vent is rarely observed in children (0.6–1 %/year) [538]. Although in children and adolescents, chronic HBV infection is generally a mild disease with a benign course, 1–5 % of HBeAg-positive children develop cirrhosis [536, 537].

Between 0.01 and 0.03 % of children with chronic HBV infection develop HCC during childhood (32 per 100,000 person-year) [538, 539]. Children developing HCC are more likely to be males (70 %), with cirrhosis (80 %), and to have undergone early seroconversion (suggesting that necroinflammation during seroconversion to anti-HBe may be severe enough to lead to cirrhosis and HCC) [538]. In adult patients, the long-term risk of both HCC and cirrhosis is directly correlated to serum HBV DNA levels and HBeAg positivity, but no conclusion can be drawn from pediatric studies because of the rarity of HCC during childhood. The role of viral genotype in the risk of developing HCC is still to be clarified in the pediatric population. The risk of HCC is higher in individuals with a family history of HCC [71].

##### Indications of treatment in children with chronic HBV infection

Decision to treat must take into account the mild evolution of the disease during childhood, the risk of disease progression later in life, the development of severe complications in few, not yet well-identified children, the efficacy of current antivirals, their side effects, and the limited number of drugs labelled for use in this age group [540].

The need for treatment should be evaluated at each follow-up visit, in order to initiate antiviral drugs at the earliest signs of liver damage. Currently, decision to start treatment is based on ALT levels, HBeAg positivity, HBV DNA levels, assessment of liver disease severity (either histology and/or noninvasive methods), family history of HCC, and co-existing liver diseases (Table 12).

**Table 12:** Indications of treatment in children with chronic HBV infection

	HBV DNA (IU/ml)	ALT	Treatment
Decompensated cirrhosis	Detectable	Any	Treat. Histology not needed. Consider LT of no stabilization
Compensated cirrhosis	Detectable	Any	Treat
Severe reactivation of chronic HBV	Detectable	Elevated	Treat immediately
Noncirrhotic HBeAg-positive CHB	>20,000	>2× ULN	Follow-up for 1 year to see for spontaneous seroconversion. Treat if no seroconversion. Histology not t needed
		1–2× ULN	Follow-up for 1 year to see for spontaneous seroconversion. If no seroconversion, assess severity of liver disease by biopsy. Treat if moderate to severe inflammation or significant fibrosis^a^
		Persistently normal (immune tolerant phase)	Monitor every 3 months. Biopsy if ALT persistently elevated or family h/o HCC or cirrhosis. Treat if moderate to severe inflammation or significant fibrosis^a^
	2000–20,000	Any ALT	Rule out other causes of elevated ALT if normal ALT. Monitor every 3 months. Biopsy if ALT persistently elevated, or with family h/o HCC or cirrhosis. Treat if moderate to severe inflammation or significant fibrosis^a^
	<2000	<ULN	Monitor every 3 months. Biopsy if ALT persistently elevated or with family h/o HCC or cirrhosis. Treat if moderate to severe inflammation or significant fibrosis^a^
		>ULN	Rule out other causes of elevated ALT. Monitor every 3 months. Biopsy if ALT persistently elevated, or with family h/o HCC or cirrhosis. Treat if moderate to severe inflammation or significant fibrosis
Noncirrhotic HBeAg-negative CHB	>2000	>2× ULN	Treat. Histology not needed
		1–2× ULN	Rule out other causes of elevated ALT. Monitor every 3 months. Biopsy if ALT persistently elevated, or with family h/o HCC or cirrhosis. Treat if moderate to severe inflammation or significant fibrosis^a^
		Persistently normal	Monitor every 3 months. Biopsy if ALT persistently elevated, or with family h/o HCC or cirrhosis. Treat if moderate to severe inflammation or significant fibrosis^a^
	<2000	>ULN	Rule out other causes of elevated ALT. Monitor every 3 months. Biopsy if ALT persistently elevated, or with family h/o HCC or cirrhosis. Treat if moderate to severe inflammation or significant fibrosis^a^
		Persistently normal	Monitor ALT every 3 months and DNA 6–12 monthly. Biopsy if ALT persistently elevated, or with family h/o HCC or cirrhosis. Treat if moderate to severe inflammation or significant fibrosis^a^

^a^A family history of HCC may warrant treatment even in children with mild histological changes, as they are at increased risk of developing HCC

As the upper limit of normal (ULN) for ALT levels in pediatric age has not yet been established, it is advised that the normal limit should be as per the local laboratory ULN. In the presence of high ALT levels, assessment of serum HBV DNA levels is important, as high HBV DNA values warrant antiviral treatment, whereas low levels should instigate investigations to exclude other causes of liver disease.

As response to currently available antivirals in children is partial and limited to specific subgroups, histological assessment of the degree of inflammation and of the stage of fibrosis is recommended before considering treatment in certain groups (Table 12). Response to both interferon (IFN)-a and NA is more likely when at least moderate necroinflammation or moderate fibrosis is found at liver histology [541, 542]. Although the benefit of treatment has not been established for children with mild inflammation or fibrosis, a family history of HCC may warrant treatment even in children with mild histological changes, as they are at increased risk of developing HCC [71]. Although still not fully validated, noninvasive methods to assess the degree of hepatic fibrosis, such as FibroScan, could prove useful to avoid liver biopsy, especially during follow-up.

However, sufficient data is lacking in children, and at present, these noninvasive methods cannot substitute for liver biopsy in the decision to treat a child or an adolescent with CHB, as these methods evaluate more fibrosis than necroinflammatory activity.

In HBeAg-positive children with elevated serum ALT levels (>1× upper normal limit), an observation period of 12 months is recommended, as raised ALT levels and variable levels of HBV-DNA may indicate imminent seroconversion that would not require treatment.

In HBeAg-negative children, ALT and HBV DNA levels should be measured every 3 months within the first year to rule out HBeAg-negative hepatitis. After confirmation of the low replicative phase (normal ALT and HBV DNA <2000 IU/ml), patients should be monitored with ALT every 3 months and HBV DNA every 6–12 months.

HCC surveillance with liver ultrasound and AFP should be done every 6 months, as in adults.

##### Treatment options for children with chronic HBV infection

The US Food and Drug Administration (FDA) approved five medications for treatment of children with CHB: IFN-a, lamivudine, adefovir, entecavir, and recently, tenofovir. IFN-a can be used in children older than 12 months of age, lamivudine starting at 3 years of age, adefovir and tenofovir in children aged 12 years and older, and entecavir starting from 16 years of age.

*IFN*-*α* Results of a large, multinational, randomized, controlled trial of IFN-α in children with HBV infection showed a virological response (defined as negativeHBeAg and HBV-DNA) in 26 % of treated patients versus 11 % of controls (*p* = 0.03) after 24 weeks of therapy. Loss of HBsAg occurred in 10 % of treated patients versus 1.2 % of controls [542]. Various studies have shown that factors associated with response to treatment are elevated ALT levels (>2× upper normal limit), low-serum HBV-DNA levels, female gender, and age <5 years [543, 544]. However, long-term follow-up studies suggest that untreated children may have similar rates of HBeAg seroconversion as IFN-α-treated children, although the seroconversion may lag by 1–3 years [545, 546]. With respect to nucleoside analogs, IFN-α has the advantages of a long-lasting response and no risk of mutants induction; however, major disadvantages are the high-cost, frequent side-effects, and the need for thrice-weekly injections. The latter could be reduced by the use of pegylated IFN-α, which requires a single weekly administration because of its prolonged half-life. It is not yet approved for use in children, although studies in adults HBV patients have shown a higher efficacy with respect o IFN-α. In summary, HBeAg seroconversion occurs earlier in IFN-α-treated children with elevated ALT levels at the time of starting therapy compared with controls. It remains to be established whether shifting the time to seroconversion by 12–36 months reduces long-term damage to the liver [531].

*Lamivudine* A large multicenter trial of LAM in children [547] showed that 23 % of the children in the treatment group cleared HBV-DNA and HBeAg, compared to 13 % in the placebo group.

The response to treatment was especially in children with higher ALT values and histological activity (among children with ALT greater than five times the upper limit of normal, HBeAg loss occurred in 50 % vs. 24 % in the placebo group). However, 19 % of children developed LAM resistant mutants. Other smaller studies of LAM treatment in children have confirmed both the efficacy in reducing serum HBV DNA and the high mutation rate [548, 549].

*Adefovir* ADV dipivoxil is approved for the treatment of adolescents (>12 years) with CHB, but not in younger children after beneficial virological effects were not observed in children between 2 and 12 years of age in the primary efficacy, multicenter, randomized trial where 23 % of adolescents reached a virological response after 12 months of ADV treatment compared with 0 % in the placebo group [550]. ADV is safe and well tolerated in children, and no important resistance-associated mutations have been observed in the pediatric setting [551].

*Entecavir* ETV is more effective than LAM and ADV in the treatment of CHB in adults. On the basis of these encouraging results and a good safety profile, ETV has been approved by the FDA for treatment of adolescents over the age of 16. Clinical trials in children younger than 16 years are ongoing.

*Tenofovir* In a recent double-blind, placebo-controlled trial on the use of tenofovir (300 mg once daily for 72 weeks vs. placebo) in CHB adolescents 12 to <18 years of age, a virological response in 89 % of treated patients was seen regardless of previous HBV therapies [552]. Normalization of ALT levels occurred in 74 % of treated patients. No resistance to tenofovir developed through week 72. Tenofovir therefore appears to be a promising agent for the treatment of CHB in adolescents, although long-term studies are needed to evaluate the rate of seroconversion and the impact on the development of HCC.

#### Treatment strategy for chronic HBV infection in children

Currently, a finite-duration IFN-a therapy remains the treatment strategy of choice for HBeAg-positive children with elevated ALT levels, as seroconversion to anti-HBe is the main aim in this patient population. IFN-a is the only available treatment offering a chance of sustained off-treatment response. It is likely that, as soon as results of trials using Peg-IFN in children are available, it will become the recommended drug. The recommended regimen is 5–10 million units per square meter, three times weekly for 6 months. For Peg-IFN, studies in adults show the highest HBeAg seroconversion rate with 48-week treatment schedules. IFN-a is the only treatment licensed for treating children younger than 3 years of age, who, however, rarely require therapy. In case of non-response at the end of IFN treatment, wait for at least 12 months before considering other therapies, as response may be achieved during the 6 months following the end of IFN-a treatment.

The recent FDA approval of tenofovir and entecavir, which have high genotypic barriers to resistance, has made them the first-line NA treatments for adolescents. In patients older than 12 years of age, tenofovir (or entecavir for patients >16 years old) is the best choice, as response rate is high and resistance is less likely. The recommended dose for tenofovir is 300 mg once daily, and for entecavir is 0.5 mg once daily (for nucleoside-naïve patients). Although not yet approved for the treatment of CHB inpatients <12 years of age, the use of tenofovir might be safe in younger children, as it is already widely used (and FDA licensed) for patients older than 2 years of age with HIV infection. Since the approval of tenofovir for adolescents, adefoviris is no longer recommended because of the higher risk of resistance and the lower response rate.

A finite-duration treatment with tenofovir or entecavir is possible if seroconversion to anti-HBe is achieved on treatment. Duration of treatments with NA has not been established, but the recommendations should be as for the adults. Patients should be monitored after discontinuation because of the possibility of post-treatment flares.

Patients who do not undergo HBeAg seroconversion on treatment, the rare children with HBeAg-negative chronic hepatitis and cirrhotic patients need long-term treatment with NA.

Tenofovir or entecavir, if allowed by the age, are the first choice. Lamivudine is the only NA currently approved for younger children. Its use should be limited to the rare young children unresponsive to IFN-a and requiring immediate treatment, and to special populations with contraindications to IFN. The recommended treatment dose for lamivudine is 3 mg/kg/day (maximum 100 mg/day), administered orally once daily.

#### Treatment failure and antiviral resistance

The basic principles remain the same as for adults. Because of the low number of effective drugs that are approved, when resistance to an NA develops in children, the decision of therapy adjustment is based on the patient’s age (Table 13).

**Table 13:** Management of antiviral resistance in children with chronic HBV infection

Lamivudine resistance	Switch to tenofovir (for >12 years old) Switch to IFN (<12 years of age)
Adefovir resistance	If the patient was NA-naive before adefovir, switch to entecavir (for >16 years age) or tenofovir (for >12 years age); entecavir for (>16 years age) may be preferred in such patients with high viremia

3.13.9 Recommendations: chronic HBV infection in children
  3.13.9.1 Any person up to the age of 18 years will be considered as a child (A1).
  3.13.9.2 The need for treatment should be evaluated at each follow-up visit, in order to initiate antiviral drugs at the earliest signs of liver damage (C2).
  3.13.9.3 Children with decompensated cirrhosis and detectable HBV DNA require urgent antiviral treatment with NA(s). Liver transplantation should be considered if patients do not stabilize with medical management (A1).
  3.13.9.4 Patients with moderate to severe activity or significant fibrosis with any ALT level should be considered for treatment (A1).
  3.13.9.5 Children with severe reactivation of chronic HBV infection should be treated without delay and irrespective of HBV DNA levels (A1).
  3.13.9.6 As the upper limit of normal (ULN) for ALT levels in pediatric age has not yet been established, it is advised that the normal limit should be as per the local laboratory ULN (C2).
  3.13.9.7 In HBeAg-positive children with elevated serum ALT levels (>1× upper normal limit), an observation period of 12 months is recommended, as raised ALT levels and variable levels of HBV-DNA may indicate imminent seroconversion that would not require treatment (C1).
  3.13.9.8 In HBeAg-negative children, ALT and HBV DNA levels should be measured every 3-months within the first year to rule out HBeAg-negative hepatitis. After confirmation of the low replicative phase (normal ALT and HBV DNA <2000 IU/ml), patients should be monitored with ALT every 3 months and HBV DNA every 6–12 months (B1).
  3.13.9.9 Treatment may be started in pre-cirrhotic chronic HBV-infected patients if they have persistently elevated ALT levels >2 times upper limit of normal (ULN) (at least 1 month between observations) and HBV DNA >20,000 IU/ml if they are HBeAg-positive and >2000 IU/ml if HBeAg-negative, even without a liver biopsy (B1).
  3.13.9.10 Patients with compensated cirrhosis and detectable HBV DNA should be considered for treatment even if ALT levels are normal (B1).
  3.13.9.11 Patients who are not considered for treatment should be followed up regularly (Table 13) (B1).
  3.13.9.12 Although the benefit of treatment has not been established for children with mild inflammation or fibrosis, a family history of HCC may warrant treatment even in children with mild histological changes, as they are at increased risk of developing HCC (B2).
  3.13.9.13 No sufficient data are available for use of noninvasive markers in children and, at present, these noninvasive methods cannot substitute for liver biopsy in the decision to treat a child or an adolescent with CHB, as these methods evaluate more fibrosis than necroinflammatory activity (C2).
  3.13.9.14 The US FDA approved five medications for treatment of children with CHB: IFN-a, lamivudine, adefovir, entecavir, and recently, tenofovir. IFN-a can be used in children older than 12 months of age, lamivudine starting at 3 years of age, adefovir and tenofovir in children aged 12 years and older, and entecavir starting from 16 years of age (A1).
  3.13.9.15 Currently, a finite-duration IFN-a therapy remains the treatment strategy of choice for HBeAg-positive children with elevated ALT levels (A1).
  3.13.9.16 In case of no response at the end of IFN treatment, at least 12 months should elapse before considering other therapies, as response may be achieved during the 6 months following the end of IFN-a treatment (B1).
  3.13.9.17 Patients who do not undergo HBeAg seroconversion on treatment, the rare children with HBeAg-negative chronic hepatitis and cirrhotic patients need long-term treatment with NA (B1).
  3.13.9.18 The recent FDA approval of tenofovir (>12 years of age) and entecavir (for >16 year of age), which have high genotypic barriers to resistance, has made them the first-line NA treatments for adolescents (A1).
  3.13.9.19 Although not yet approved for the treatment of CHB in patients <12 years of age, the use of tenofovir might be safe in younger children, as it is already widely used (and FDA-licensed) for patients older than 2 years of age with HIV infection (B1).

### 3.14 Treatment of acute HBV infection

The natural course of HBV infection is determined by the interplay between virus replication and the host’s immune response. Upon exposure to HBV, individuals with a vigorous and broad immune response to the virus develop an acute self-limited infection that may result in acute hepatitis; an aberrant response can lead to fulminant hepatitis. Individuals who do not mount a broad and vigorous immune response do not clear the virus, but develop persistent infection and become chronically infected with HBV.

#### Clinical manifestations

During the acute phase of hepatitis B (AVH-B), manifestations range from subclinical or anicteric hepatitis to icteric hepatitis, and in some cases, fulminant hepatitis. Approximately 70 % of patients with acute hepatitis B have subclinical or anicteric hepatitis, while 30 % develop icteric hepatitis. The course of acute hepatitis B is divided into the incubation period, and preicteric, icteric and convalescence phases. From the incubation period to the onset of symptoms or jaundice, it averages 75 days (range 40–140 days). The onset of hepatitis B is typically insidious, with nonspecific symptoms of malaise, poor appetite, nausea and pain in the right upper quadrant. With the onset of the icteric phase, symptoms of fatigue and anorexia typically worsen. Jaundice can last from a few days to several months, the average being 2–3 weeks. Itching and pale stools may occur. The convalescent phase of hepatitis B begins with the resolution of jaundice. Fatigue is generally the last symptom to abate and may persist for many months into convalescence.

The physical signs of typical acute hepatitis B are not prominent. Variable degrees of jaundice are present. The only other common physical finding in acute hepatitis B is a mild and slightly tender hepatomegaly. Mild enlargement of the spleen or lymph nodes occur uncommonly.

#### Pathogenesis

It is clear that replication and persistence of HBV is not cytopathic per se. Studies in acutely HBV-infected chimpanzees and woodchucks showed that no host response to viral replication occurred during the incubation phase, as HBV infection does not stimulate the innate immune system, which recognizes pathogen-associated molecular patterns. In contrast, later in the infection period, most of the effector molecules associated with the adaptive cellular immune response are induced, followed by HBV antibodies. HBV elimination starts several weeks before onset of the disease with T-cell-dependent noncytolytic mechanisms, but later cytolytic immune responses follow and generate the symptoms of acute hepatitis [553].

High disease activity usually leads to clinical and serological resolution. However, even after serological resolution, small amounts of cccDNA persist in the liver for years, decades and possibly for life. T cell immunity suppresses viral replication originating from these cccDNA copies to very low levels [554]. Anti-HBc appears with the onset of the disease as the first anti-HBV antibody, then anti-HBe, anti-pre-S, and finally, anti-SHBs. These antibodies probably contribute neither to virus elimination from the liver nor to the pathogenesis of hepatitis. However, anti-HBs formed during convalescence and later may enhance opsonization of HBsAg and block de novo infection of hepatocytes by released HBV. In contrast to the other HBV antibodies, anti-HBc induction is partially T cell independent. This explains the presence of anti-HBc even in those patients who do not build up an efficient immune response. Serological resolution is defined by the disappearance of HBsAg, which may take months after onset.

In subjects who have been previously vaccinated, there is earlier engagement of innate and adaptive immunity at much lower viral loads, leading to blunted viral load increase and rapid clearance of virus, thus preventing development of clinically significant acute and chronic HBV infection [555].

#### Diagnosis

The differential diagnosis of HBsAg-positive acute hepatitis includes reactivation (flare or exacerbation) of hepatitis in chronic HBV-infected patients.

Laboratory testing during the acute phase of acute hepatitis B reveals elevations in the concentration of alanine and aspartate aminotransferase levels (ALT and AST); values up to 1000–2000 IU/l are typically seen during the acute phase, with ALT being higher than AST. The serum alkaline phosphatase and lactic dehydrogenase are usually only mildly elevated (less than threefold). The bilirubin is variably increased, in both direct and indirect fractions. The serum bilirubin concentration may be normal in patients with anicteric hepatitis. Serum albumin rarely falls except with protracted severe disease. The prothrombin time can increase and is the most reliable marker of severity of injury. In patients who recover, normalization of serum aminotransferases usually occurs within 1–4 months. Persistent elevation of serum ALT for more than 6 months may indicate progression to chronic hepatitis. Various auto-antibodies can appear during the course of acute hepatitis B, most typically to smooth muscle.

The diagnosis of acute hepatitis B is based upon the detection of HBsAg and IgM anti-HBc. During the initial phase of infection, markers of HBV replication, HBeAg and HBV DNA, are also present. Recovery is accompanied by the disappearance of HBV DNA, HBeAg to anti-HBe seroconversion, and subsequently HBsAg to anti-HBs seroconversion. During resolving acute hepatitis B, anti-HBe appears after anti-HBc, but before anti-HBs. It usually disappears earlier than anti-HBs.

Rarely, patients present during the window period when HBsAg has become negative but anti-HBs is not yet positive. In this setting, which is more common in patients with fulminant hepatitis B in whom virus clearance tends to be more rapid, IgM anti-HBc is the sole marker of acute HBV infection.

In acute infections, HBsAg concentrations rise logarithmically for weeks–months from undetectable to typical final concentrations of 10,000–100,000 ng/ml with 2–4 days of doubling time [556]. If the acute HBV infection is resolved, HBsAg decreases with an initial half-life of 8 days until it has been completely removed from serum after weeks–months. In about 25 % of acute resolving hepatitis B cases, the elimination of HBsAg proceeds much faster, with the consequence that samples taken in the late acute phase may be HBsAg negative [557]. A decrease in HBsAg concentration by more than 50 % within the first 4 weeks indicates resolving acute infection in >95 % of cases [558]. Hence, quantitative analysis of highly concentrated HBsAg is an excellent prognostic marker, indicating progression to chronicity if the values remain stable or increase.

Anti-HBc immunoglobulin (Ig)M (anti-HBc IgM) may be useful in two situations: (1) to distinguish an acute hepatitis caused by HBV from a hepatitis of different etiology in a chronic HBV-infected patient; and (2) to identify an acute hepatitis in some hepatitis B patients, particularly those with fulminant hepatitis B or HDV coinfection, where HBsAg may have been eliminated very rapidly. Predominant TH1 immune response in AVH-B favors cell-mediating viral clearance, while TH2-mediated immune response in chronic HBV infection favors antibody production. HBV antigens elicit immune-mediated liver injury in a dose-dependent manner; therefore, low viral antigen load and subsequent resolution of infection in AVH-B as compared to persistent viral antigenemia in chronic HBV infection leads to significantly increased production of HBV specific antibodies (mainly Anti HBe/Anti HBc) in chronic HBV infection or its exacerbation in comparison to AVH-B [559]. Tests should be quantitative because anti-HBc IgM is also positive in CHB and during convalescence. Levels >600 Paul–Ehrlich units/ml or IgM anti-HBc (>1:1000) suggest an acute HBV infection with high inflammatory activity. In all other situations, concentrations are lower or undetectable [23, 319]. In a study on patients with a protracted clinical course of >2 months with elevated liver enzymes and positive HBV DNA, it was found that peak bilirubin level, peak AST levels and least platelet count within the first 8 weeks had the highest predictive power for differentiating patients with CHB with acute flare from acute hepatitis B. Bilirubin, AST and platelet count (BAP) score was calculated, and a score of >2 strongly suggested an acute flare of CHB [560].

The meaning of the term anti-HBs is somewhat ambiguous. Some understand it to mean antibodies only against the small HBsAg protein (SHBs), others the entire antibody spectrum against all three surface proteins including pre-S1 and pre-S2. During acute infection, anti-pre-S antibodies appear before anti-SHBs, and they often coexist with HBsAg.

#### Outcome of acute hepatitis B

Fulminant hepatitis B is an atypical course for acute hepatitis B infection, occurring in <1 % of icteric cases. Typically, in fulminant disease, HBV DNA and HBeAg become undetectable as hepatic failure supervenes.

The rate of progression from acute to chronic hepatitis B is determined primarily by the age at infection. The rate is approximately 90 % for a perinatally acquired infection, 20–50 % for infections between the age of 1 and 5 years and <5 % for an adult-acquired infection [561]. Genotype A was an independent risk factor for progression to chronic infection following AVH-B in Japan [562]. In Japanese patients, high levels of HBsAg at 12 weeks and HBV DNA at 8 weeks were useful for discriminating between the patients who lost HBsAg within 12 months and those who did not. Only those who fail to clear HBV within 12 months from the onset may develop chronic infection [563].

#### Treatment

Treatment for acute HBV is mainly supportive. In addition, appropriate measures should be taken to prevent infection in exposed contacts.

Patients who have a coagulopathy, are deeply jaundiced, are encephalopathic or cannot tolerate oral intake should generally be hospitalized.

Whether patients should be treated with nucleos(t)ide therapy is unsettled since few studies have addressed the benefits of antiviral therapy during acute infection. One prospective case series treated 15 patients with severe AHB (INR >1.6, serum bilirubin levels >10 mg/dl or hepatic encephalopathy) with 100 mg of lamivudine, achieving a response rate of 86 % [564]. The first randomized clinical trial included a total of 71 patients with AHB (31 randomized to lamivudine for 3 months and 40 to placebo) and showed no biochemical or clinical benefit to lamivudine; the lack of response to therapy was also observed in the subset of patients with severe AHB. There was also no difference in HBsAg loss after 12 months (94 vs. 97 % in the groups that received lamivudine and placebo, respectively) [565]. However, another RCT that included 80 AVH-B patients showed statistically significant differences in mortality (7.5 % lamivudine vs. 25 % placebo) and incidence of acute liver failure (20 vs. 42.5 %). The study also showed that the sooner the treatment is initiated, the better the results obtained, and a rapid decline of HBV DNA load was a good predictor for the treatment outcome [566]. In a few other studies, patients with severe acute or fulminant hepatitis B were treated with lamivudine, demonstrating the safety and efficacy of this antiviral drug, with a capacity for improving the prognosis of these patients [567–569]. Antivirals other than lamivudine have been investigated so far, in small case reports or series of acute severe hepatitis B, with some promising preliminary results with the use of entecavir [570, 571], tenofovir [572, 573], and telbivudine [574].

Thus, antiviral therapy is not indicated in the vast majority of patients with acute hepatitis B, but may be indicated in certain subgroups of patients as follows: (a) patients with fulminant acute hepatitis B; (b) severe AVH-B: individuals who fulfill any two of the following criteria: (1) hepatic encephalopathy; (2) serum bilirubin >10.0 mg/dl; and (3) international normalized ratio (INR) >1.6, especially if it is increasing; and (c) a protracted course [such as persistent symptoms or marked jaundice (bilirubin >10 mg/dl) for more than 4 weeks after presentation].

These indications outline the limitations in differentiating AVH-B from reactivation of chronic HBV infection. An argument can be made for treating all of the above groups of patients using an NA, given its safety and the fact that many of these patients may ultimately need liver transplantation and reduction of HBV DNA levels would reduce the risk of recurrent hepatitis B after transplant.

Interferon should be avoided because of the increased risk of hepatic necro-inflammation. Telbivudine, lamivudine, adefovir, entecavir or tenofovir are acceptable options when given as monotherapy, as the duration of treatment should be short. Treatment can be stopped after confirmation that the patient has cleared HBsAg.

3.14 Recommendations (acute viral hepatitis B)
  3.14.1 Establishing a diagnosis of acute HBV is important, as majority of adult patients presenting as acute hepatitis B have reactivation of CHB. A definite history of exposure, positive HBeAg and IgM antiHBc with low HBV DNA levels and liver biopsy in doubtful cases can help to establish the diagnosis of acute HBV infection and exclude the diagnosis of HBV reactivation (B1).
  3.14.2 More than 95–99 % of adults with acute HBV infection will recover spontaneously and seroconvert to anti-HBs without antiviral therapy (A1).
  3.14.3 Patients with fulminant hepatitis B must be evaluated for liver transplantation (A1).
  3.14.4 Treatment is only indicated for patients with fulminant hepatitis B or for those with severe or protracted acute hepatitis B (C2).
  3.14.5 Tenofovir, entecavir, telbivudine, lamivudine or adefovir are acceptable options when given as monotherapy, as the duration of treatment should be short (C2).
  3.14.6 The duration of treatment is not established. However, treatment should be continued until HBsAg clearance is confirmed, or indefinitely in those who undergo liver transplantation (C2).
  3.14.7 Interferon is contraindicated (A1).
  3.14.8 When the distinction between true severe acute hepatitis B and spontaneous reactivation of chronic HBV infection is difficult, NA treatment should be administered (A1).

### 3.15 Antiviral prophylaxis before immunosuppressive therapy or chemotherapy

Chemotherapy-induced HBV reactivation and hepatitis flare is a common complication in HBsAg(+) cancer patients, with the incidence ranging from 20 to 70 % in previous reports [575]. Increased incidence of HBV reactivation was associated with cancer types (lymphoma, breast cancer, HCC), viral factors (high baseline HBV DNA, HBeAg positivity), and types of anti-cancer therapy (steroid, anthracyclines). All candidates for chemotherapy and immunosuppressive therapy should be screened forHBsAg and anti-HBc prior to initiation of treatment. Vaccination of HBV seronegative patients should be considered. Higher vaccine doses may be required to achieve anti-HBs response in immunocompromised patients.

The efficacy of prophylactic anti-viral therapy in preventing HBV reactivation in HBsAg(+) patients was firmly established by two randomized trials in lymphoma patients and meta-analysis involving clinical trials and cohort studies of various cancer types [576–578]. Lamivudine was used in all of these studies and was shown to reduce the risk of HBV reactivation [risk ratio (RR) 0.13, 95 % CI 0.07–0.24], reactivation-related mortality (RR 0.30, 95 % CI 0.1–0.94), and to reduce the delay/premature termination of chemotherapy (RR 0.41, 95 % CI 0.27–0.63) [579]. The optimal duration of lamivudine prophylaxis was not explored in these studies, and current recommendation for the duration of anti-viral prophylaxis is 6–12 months after completion of chemotherapy [25, 105]. It is not known whether more potent anti-viral agents, such as entecavir and tenofovir, can further improve the prophylactic efficacy in reducing the risk of HBV reactivation or reactivation-related mortality. However, these agents should be considered if prolonged anti-viral therapy is indicated, because of their lower rate of treatment-induced HBV resistance.

HBV reactivation has also been reported in HBsAg(+) cancer patients who received other molecular target therapies. In the case of mTOR (mammalian target of rapamycin) inhibitor, everolimus is approved for the treatment of neuroendocrine tumor and renal cell carcinoma (as single-agent), and breast cancer (in combination with hormonal therapy) [580, 581]. This may be due to the effects of everolimus (and other mTOR inhibitors) on immune suppression or on HBV synthesis [582].

Immunosuppressive therapy is required for patients who undergo solid organ transplantation, and long-term anti-viral therapy is recommended for HBsAg(+) organ transplant recipients [583]. Immunosuppressive therapy, including steroid, cytotoxics, and biological agents (e.g., tumor necrosis factor-α-blocking agents), is also commonly used in patients with inflammatory bowel disease and rheumatic diseases. Although prospective studies in these patient populations are lacking, the incidence and severity of HBV reactivation has generally correlated with the extent of immune suppression, and fatal HBV reactivation has been reported [584–586]. Therefore, despite the lack of randomized clinical trials, prophylactic anti-viral therapy is recommended for HBsAg(+) patients who received immunosuppressive agents for auto-immune and rheumatic diseases. However, the duration may be long-term, and its cost-effectiveness is not yet established.

Chemotherapy-induced HBV reactivation in patients with ‘resolved’ HBV infection (i.e., patients who are negative for HBsAg but positive for anti-surface (anti-HBs) or anti-core (anti-HBc) antibodies) is also mostly reported in lymphoma patients who received rituximab-containing regimens [587–592]. The cumulative risk of hepatitis-related mortality in these early, retrospective series, in which no preventive strategies were adopted, was about 1 %. Two prospective studies exploring different preventive strategies were recently reported. Hsu et al. reported prospective follow-up of HBV DNA and entecavir therapy upon HBV DNA reactivation in lymphoma patients who received rituximab-based chemotherapy [593, 594]. The incidence of HBV DNA reactivation was 10–40 %, depending on the sensitivity of the HBV DNA test and the diagnostic criteria for HBV reactivation. Huang et al. [595] compared prophylactic entecavir treatment and therapeutic (started when HBV DNA reactivation was confirmed) entecavir treatment in lymphoma patients who received rituximab-CHOP chemotherapy, and confirmed that prophylactic entecavir treatment significantly reduced the risk of HBV reactivation. In these studies, the incidence of HBV-related hepatitis flare in patients with HBV DNA reactivation was <50 %, and no HBV-related liver decompensation or death was noted. No risk factors for HBV reactivation were identified, though baseline anti-HBs titer was proposed. Physicians should be aware of the potential life-threatening consequence of HBV reactivation in this patient population. However, the optimal preventive strategy remains undetermined. RCT has clearly demonstrated the efficacy of prophylactic anti-HBV in high-risk lymphoma patients with resolved HBV infections. Further studies to identify host and viral risk factors for HBV reactivation and cost-effectiveness of different preventive strategies are clearly needed.

Incidence and severity of HBV reactivation in patients with resolved HBV infection who received other immunosuppressive agents are not well defined [596]. HBsAg-negative patients with positive anti-HBc antibodies should be tested for HBV DNA. HBsAg-negative, anti-HBc positive patients with detectable serum HBV DNA should be treated similarly to HBsAg positive patients. HBsAg-negative, anti-HBc positive patients with undetectable serum HBV DNA, and who receive chemotherapy and/or immunosuppression regardless of anti-HBs status, should be followed carefully by means of ALT and HBV DNA testing, and be treated with NA therapy upon confirmation of HBV reactivation before ALT elevation [25]. The frequency of monitoring can range from 1 to 3 months, depending on the type of immunosuppressive therapy and comorbidities. Some experts recommend prophylaxis in all HBsAg-negative, anti-HBc positive patients who receive rituximab and/or combined regimens for hematological malignancies, if they are anti-HBs negative and/or if close monitoring of HBV DNA is not guaranteed [597–599].

NA prophylaxis is also recommended for anti-HBc positive patients receiving bone marrow or stem cell transplantation [599, 600]. The optimal duration of prophylaxis for these indications is not known.

3.15 Recommendations: antiviral prophylaxis before immunosuppressive therapy or chemotherapy
  3.15.1 All candidates for chemotherapy and immunosuppressive therapy should be screened for HBsAg and anti-HBc prior to initiation of treatment (A1).
  3.15.2 Prophylactic anti-viral therapy should be given to HBsAg(+) cancer patients who receive cytotoxic or immunosuppressive therapy, both during therapy (regardless of HBV DNA levels) and for 12 months after cessation of therapy to reduce the incidence and severity of HBV reactivation (A1).
  3.15.3 Physicians should be aware of the risk of HBV reactivation in lymphoma patients with resolved HBV infection [HBsAg(−) and anti-HBc(+) who receive rituximab-containing chemotherapy]. Further studies are needed to compare the efficacy and cost-effectiveness of different preventive strategies (prophylactic antiviral therapy vs. regular HBV DNA monitoring) (B1).
  3.15.4 HBsAg-negative patients with positive anti-HBc antibodies should be tested for HBV DNA. HBsAg-negative, anti-HBc positive patients with detectable serum HBV DNA should be treated similarly to HBsAg-positive patients (C1).
  3.15.5 HBsAg-negative, anti-HBc positive patients with undetectable serum HBV DNA and who receive chemotherapy and/or immunosuppression regardless of anti-HBs status should be followed carefully by means of ALT and HBV DNA testing, and be treated with NA therapy upon confirmation of HBV reactivation before ALT elevation (C1).

### 3.16 Public health issues for HBV: prevention and management

#### Needles and other sharp instruments

It has been well established that HBV can be spread by contaminated needles, including intravenous drug use, accupuncture, tattoos, ear piercing and needle prick injuries in hospital situation. This can be prevented by raising awareness and by public education. In more developed countries, disposable needles are used for accupuncture and ear piercing. The use of disposable needles/instruments is more difficult to implement. The importance of implementing safe sharps practices in the hospital setting cannot be over emphasized. Other than the use of disposable needles and sharps boxes, education and surveillance concerning the disposal of sharps, the banning of recapping needles, the transfer of blood from syringes into containers, and needle disassembly should be enforced.

#### Transfusion services

There has been widespread implementation of screening for HBsAg (and anti-HCV as well as anti-HIV) in the transfusion services in most countries in Asia. However, with the use of potent immunosuppressors, especially anti-CD20s such as rituximab and ofatumumab, it becomes increasingly important for transfusion services to screen for occult hepatitis B, since such recipients may develop severe/fulminant hepatitis B. This would require the use of a nucleic acid test (NAT) to quantify small amount of HBV DNA [601]. The great expense for such testing is a potential limitation, but NAT has become mandatory in more developed countries.

#### Prevention of maternal to child transmission of the hepatitis B virus: vaccination and antiviral treatment

The risk of maternal to child transmission of HBV had been well documented, mostly from studies from Taiwan, prior to the development of the hepatitis B vaccine in 1981 [602]. Up to 63 % of infants born of HBsAg-positive mothers became HBsAg-positive during the first 6 months of life. Six percent of fathers and 67 % of siblings were also HBsAg-positive. Infants born of HBeAg-positive mothers have a higher chronic HBV positivity rate compared to those born of HBeAg-negative mothers, proving that transmission is related to high viral load. However, up to 25–30 % of infants born of HBeAg-negative mothers also become chronic HBsAg positive, showing that HBeAg-negative mothers can also have high viral load. It has subsequently also been shown by sequence analysis of HBV mutations that post-natal transmission can occur from HBsAg-positive fathers and even aunts [603]. With the availability of both hepatitis B immune globulin (HBIG) and hepatitis B vaccine (at first plasma-derived, later recombinant), there was marked reduction in the infant infection rate [125]. In one of the most carefully planned studies, the infant chronic HBV positivity state was reduced from 73.2 % in the control group to 21.0 % in the vaccine alone group, 6.8 % in the group receiving vaccine plus one dose of HBIG and 2.9 % in the group receiving vaccine plus multiple doses of HBIG (*p* ≤ 0.0001 for all groups) [604]. With increased knowledge of, and better assays for, HBV DNA, it has recently been shown in a retrospective study of 869 HBsAg-positive mothers and their infants who had received HBIG with three does of hepatitis B vaccine, that 27 infants (3.1 %) were HBsAg-positive at age 7–12 months [605]. Multivariate analysis showed that maternal HBV DNA levels and detectable HBV DNA in the cord blood were independent risk factors for immunoprophylaxis failure. All failures occurred in infants born of HBeAg-positive mothers with pre-delivery HBV DNA ≥6 log_10_ copies/ml. Other smaller studies also confirm that high maternal viral load (in the study of Wiseman et al. HBV DNA of >8 log_10_ copies/ml) is associated with failure of prophylaxis [126, 606]. Since it is possible that mothers with HBV DNA levels between 6 and 8 log_10_ copies/ml can still induce immuonprophylaxis failure in their infants, it is advisable to treat mothers with antiviral therapy when their HBV DNA levels are ≥6 log_10_ copies/ml. There have been long-term follow-up studies of vaccinated infants, one of which follow the vaccine recipients for 22 years [606]. Booster doses are probably not necessary for immune competent subjects, because of good anamnestic responses even after the anti-HBs titers have fallen to very low levels (<10 mIU/ml).

#### Increasing the awareness of the public and medical personnel

Education of public and health care professionals will help in identification of persons at risk for viral hepatitis, and ensure appropriate counseling, diagnosis, medical management, and treatment [607]. Appropriate training for medical personnel is important.

#### Shift in focus from tertiary care to community and primary care settings

The management of CHB requires a shift in focus from tertiary care to community and primary care settings. This could also include an exploration of alternative arrangements for care, including possible roles for nurse practitioners or hepatitis coordinators besides primary care doctors. Primary care services, particularly those working in high prevalence areas, and community organizations providing support and advice to priority populations will need to play an increasingly important role in hepatitis B screening, testing and monitoring. Better understanding of hepatitis B and C and its management is also required for some primary care practitioners and non-hepatology specialists such as those involved in antenatal care, where in some cases, maternal treatment can significantly reduce the risk of transmission of HBV to the baby. A 6-year study from China reported that the training of general practitioners (GPs) of village clinics in Hebei province improved their practice, for instance, the sterilization of needles, syringes and transfusion sets. The chronic HBV positivity rate of 2-year-old children (mothers are HBsAg negative) dropped from 11.6 to 2.1 %, which indicates that the training of GPs decreases the transinfection rate of HBV [608].

3.16 Recommendations: public health issues for HBV-prevention and management
  3.16.1 The general public should be educated concerning care in using needles and other sharp instruments (A1).
  3.16.2 Hospitals should strongly enforce the implementation of safe sharps practices (A1).
  3.16.3 Transfusion services should be encouraged to use NAT as screening tests (B1).
  3.16.4 Universal hepatitis B vaccination of newborns should be enforced (A1).
  3.16.5 Increasing the awareness of the public and medical personnel should be a priority (A1).
  3.16.6 Appropriate training for medical personnel at various levels is important (A1).
  3.16.7 A shift in focus from tertiary care to community and primary care settings is needed (A1).

### 3.17 Occult HBV infection

#### Definition and patient category

Occult hepatitis B (OBI) infection is defined by detectable HBV DNA in serum and/or liver in patients who are tested negative for serum HBsAg by the most sensitive commercial assays [609]. There are three groups of subjects in whom HBV DNA is detectable with concomitant undetectable serum HBsAg. 

For the first group, subjects are in the window phase of HBV infection, exposed recently. Depending on the immune status at the time of contacting HBV, the subjects may have acute hepatitis B with resolution of the disease or become chronically infected with hepatitis B. They are therefore regarded as subjects with past infection and subjects with chronic HBV infection, respectively, in subsequent follow-ups. In the former group, subjects would have positive or negative anti-HBs and anti-HBc in subsequent follow-ups. It is, however, important to note that studies have shown that HBV DNA may still be detectable in some of these subjects even after years of so-called acute HBV infection [610, 611]. These subjects may also be having OBI. More longitudinal studies are required to delineate the outcome of acute HBV infection in this regard.

For the second group, patients are considered as having primary OBI. These patients have been identified only by persistently detectable HBV DNA without prior documentation of HBsAg positivity before the presentation.

For the third group, patients have known chronic HBV infection with previous documentation of HBsAg positivity for at least 6 months and are undergoing subsequent HBsAg seroclearance, i.e., entering into the last phase of chronic HBV infection. Around 50–60 % of these patients are positive for anti-HBs [612].

OBI can also be serologically classified into sero-positive (anti-HBs and/or anti-HBc positive) or sero-negative (both anti-HBs and anti-HBc negative). It is estimated that upto a total of 20 % of OBI patients are negative for all serological markers of HBV infection [613]. These serologically negative OBI patients may likely be infected with minute amounts of HBV which are insufficient to mount intense and specific immune responses.

#### Prevalence of OBI

There is a wide range of estimation of the prevalence of OBI reported in different countries. It ranges from <1 to 18 % [614–618]. These data are grossly underestimated, and this is related to the fact that most of the OBI patients have extremely low HBV DNA levels in the serum (and liver tissues are generally not easily assessible). Although the viremia level (HBV DNA) is generally quoted as lower than 200 IU/ml [613], at least more than 90 % of OBI patients will have HBV DNA levels of <20 IU/ml in the serum [616]. These low levels of HBV DNA as well as their fluctuations make the detection of this condition difficult even when using existing standardized and sensitive HBV DNA assays.

#### Pathogenesis of OBI

Mechanisms leading to OBI remain obscure. Proposed mechanisms include mutations of viral genomes, especially over the surface gene (e.g., G145R), such that they escape detection by commercial HBsAg assays [619]. However, studies have shown that there is an absence of relevant mutations in the genomic HBsAg coding region [620, 621]. Another better accepted postulation is that in OBI patients, the HBV is replicating at an extremely low rate [622]. This can either be due to intrinsically low viral replicative activities or extrinsic factors; namely, an immense immune suppressive effect on the HBV. Several studies have found that there are significantly more genomic mutations and rearrangement in splice donor sites of the pre-S1, pre-S2, and S genes and their regulatory regions [623, 624]. Other studies reported greater nucleoside and amino acid diversities in OBI compared to those of overt chronic HBV infection [621, 623]. Additive effects from these mutations may restrain the virus replication capacity. Post-transcriptional mechanism involving the Pre-S2/S RNA splicing has also been proposed to explain the marked decrease in pre-S2/S transcript and HBsAg [625]. On the other hand, reactivation of HBV from OBI during and after immunosuppressive therapy (including anti-CD20) indirectly suggests that the OBI state is kept by immune-mediated suppression of virus replication [626]. In fact, it has been shown that human genomic constitutions, in particular, the HLA DP region as illustrated by studies using single nucleotide polymorphisms (SNP) affecting the immune responses, are associated with the chance of HBV chronicity [627], HBV disease activity [628] and also the chance of loss of HBsAg seroclearance [629].

#### Clinical scenarios of OBI

OBI is of particularly interest in three main clinical areas. First, whether HBV is transmissible from OBI patients. Second, what are the clinical manifestations of OBI, including liver function, histological features, and long-term complications, e.g., liver cirrhosis and HCC? Finally, what is the risk of reactivation of HBV from OBI patients who have undergone immunosuppressive therapy?

There are several studies addressing the issue of transmissibility of HBV through the blood products from OBI subjects. It has been shown that while HBV transmission is possible, the risk is relatively low (1–3 %) [630]. Factors affecting the chance of infection of recipients include the anti-HBs status in the donors and the recipients, the blood/product volume received by the recipients, and the immune status of the recipients [631]. There has been a practice of anti-viral prophylaxis being given to recipients receiving bone marrow or solid organ donations from OBI subjects. Many centers advocate the use of nucleos(t)ide analogs for recipients who received bone marrow/organs from donors who are anti-HBc positive with or without detectable HBV DNA.

There are many studies examining the possible pathogenic role of OBI. According to several studies, nearly all OBI patients will have normal liver biochemistry and minimal or no necroinflammation and fibrosis in liver histology [632, 633]. However, OBI may still be associated with the development of liver cirrhosis and HCC. OBI as the etiology for development of cirrhosis and HCC is well reported in the setting of coinfection with chronic hepatitis C infection [634]. The estimated frequency of OBI in patients with cryptogenic liver cirrhosis ranges from 4.8 to 40 % [635, 636].

45–80 % of patients with apparently unidentifiable cause of HCC have had HBV detected in the liver, suggesting that OBI is associated with increased risk of HCC [637, 638]. A longitudinal follow-up study conducted in Japan confirmed OBI increased the risk of HCC [639]. A recent meta-analysis recruiting 16 studies revealed that OBI increased the risk of development of HCC, with an adjusted odds ratio of 2.9 from five prospective studies [634]. This was confirmed by another meta-analysis that included 14 studies showing increased risk of HCC in OBI subjects with an OR of 8.9 [640]. Possible mechanisms for OBI leading to these complications include (1) persistent low-grade inflammation leading to or continuing with existing cirrhosis [641]; (2) persistent oncogenic role of the HBV genome with its possible integration into the human genome as well as with its free episome [642]; and (3) low levels of HBV transcriptional activities with viral protein synthesis (e.g., X protein and truncated preS–S protein) with transforming properties [643].

HBV reactivation in OBI subjects undergoing immunosuppressive therapy has recently gained increasing attention because of the potential fatal hepatic decompensation if the condition is not treated promptly (see “3.15 Antiviral prophylaxis before immunosuppressive therapy or chemotherapy” section).

Concerning the antiviral treatment, it is recommended that whenever HBV DNA is detectable in the serum of HBsAg-negative and anti-HBc/anti-HBs-positive patients at baseline, antiviral treatment should be given as in the case of HBsAg-positive patients. Patients who are negative for HBV DNA at baseline should have HBV DNA and liver function checked at regular intervals of 1–3 months unil at least 12 months after the last cycle of immunosuppressive therapy. The frequency of monitoring depends on which agents are being used (for example patients on rituximab should be checked more frequently). HBV DNA levels are more sensitive indices of reactivation than liver function since they become detectable before ALT levels start to increase. For those with undetectable HBV DNA at baseline, once HBV DNA is detectable on follow-up, patients should be treated with nucleos(t)ide analogues. It has also been suggested that patients should be treat pre-emptively if they are anti-HBs negative or if close follow-up cannot be assured [597, 598].

To date, there are insufficient data to recommend whether routine antiviral prophylaxis right at the initiation of immunosuppressive therapy or postponement of antiviral agents until HBV DNA becomes undetectable is more appropriate. In addition, there is no good data on the frequency of monitoring of HBV DNA and HBsAg during and after immunosuppressive therapy. According to a recent study adopting 4 weekly HBV DNA monitoring in HBsAg-negative, anti-HBc positive patients receiving rituximab and prompt entecavir treatment once the HBV DNA is detectable, all of the patients achieved excellent control [594].

3.17 Recommendations: occult HBV infection
  3.17.1 Occult hepatitis B infection is not an uncommon disease entity. Suspicion should be raised in all HBsAg-negative subjects with or without positive anti-HBs or anti-HBc (C1).
  3.17.2 Sensitive nucleic acid tests should be used to screen all blood donations from HBsAg-negative subjects. Transfusion products should be discarded if HBV DNA is detectable in these products (A1).
  3.17.3 HBV DNA measurement in serum and liver (if available) by highly sensitive assays should be performed in patients with cirrhosis and/or HCC in which no causes are identifiable (B1).
  3.17.4 Chronic hepatitis B patients with HBsAg seroclearance still require continuous follow-up for the development of cirrhosis-related complications and HCC (A1).
  3.17.5 HBsAg negative, anti-HBc positive subjects with or without positive anti-HBs should be closely monitored by HBV DNA during and at least 12 months after immunosuppressive therapy. Monitoring should be more frequent in patients receiving potent B cell depletion agents, e.g., anti-CD20. Antiviral treatment should be started once the HBV DNA is detectable (B1).

## 4 Newer therapies and future perspectives

### 4.1 Newer therapies and immunomodulatory therapies

The limited efficacy of the currently available antiviral treatments requires the development of new therapeutic tools for the treatment of CHB. Promising therapies have recently been developed that directly target HBV-infected hepatocytes by inducing cccDNA degradation or by inhibiting HBV entry or the expression of viral proteins. HBV-infected hepatocytes may also be targeted by immunotherapeutic approaches designed to either boost the HBV-specific T cell component of the immune response or to directly stimulate the intrahepatic innate response [644]. The efficacy and feasibility of these approaches will, however, need to be carefully evaluated in humans.

#### Antiviral therapies targeting hepatitis B virus-infected hepatocytes

The life cycle of the virus begins with its attachment to the appropriate hepatocyte receptor, which is now recognized to be a bile salt transporter known as sodium taurocholate co-transporting polypeptide (NTCP) [645]. The region between amino acids 21–47 of the Pre-S1 present in l-HBsAg in virus binds to the hepatocyte membrane. Cyclosporine (known to inhibit NTCP) analogues without its immunosuppressive properties and oxysterols [646] may thus constitute possible drugs for development against HBV for the future. Myrcludex-B, a synthetic lipopeptide ligand derived from the pre-S1 domain of l-HBsAg blocks de novo HBV infection both in vitro and in vivo, as demonstrated after pretreatment of human chimeric uPA/SCID mice [647]. Six weeks of Myrcludex administration, initiated either 3 days or 3 weeks post infection in the same animal model, efficiently blocked cell-to-cell virus spread and cccDNA amplification [648]. Although the above drugs appear to block HBV at the point of entry and therefore prevent the infection of new hepatocytes, their use as monotherapy regimens is unlikely to prove very effective unless there is an obvious effect on already infected hepatocytes harboring transcriptionally active cccDNA. Therefore, future regimens may include such drugs only in combination with others.

Following attachment, the processes of endocytosis, uncoating and delivery of the resulting naked nucleocapsids to the nuclear pores are initiated. Ezetimibe was tested using the HepaRG cell model and was shown to inhibit the establishment of intrahepatic cccDNA and expression of viral replication markers when the cells were infected with HBV. These findings indicate that the drug acts at early stages in the life cycle of the virus by modulating hepatic cholesterol uptake and interfering with lipid transport, pathways that may represent new targets for antiviral therapy in the case of HBV infection [649].

Nucleocapsid disassembly occurs at the nuclear pore, followed by translocation to the nucleoplasm of the released HBV-DNA. Within the nucleus, the rcDNA is converted into a double-stranded cccDNA molecule. This involves a number of stages. In this form, cccDNA is quite stable and behaves as a minichromosome, being the template for viral transcript synthesis by host RNA polymerase II. Most HBV-specific antiviral agents have thus far been unable to prevent the replenishment of the cccDNA pool from maturing HBV-DNA containing nucleocapsids, which are recycled to the nucleus from the cytoplasm, or to effect efficient clearance of cccDNA-containing hepatocytes. In the last few years, new strategies aimed at improving cccDNA clearance have been developed. These include lymphotoxin-β receptor (LT-β R) activation of HBV-infected cells [650], and cccDNA-specific transcription activator-like effector nucleases (TALENs) [651]. An alternative approach is to modulate the expression of viral proteins, such as HBsAg and HBeAg, which are believed to play a role in induction of T cell exhaustion. This could potentially be achieved by using RNA interference-based therapeutics that target expression of specific viral RNAs [652].

Viral messenger RNAs are translated in the cytoplasm to yield viral proteins. Once synthesized, the polymerase engages, an event that leads to recruitment of core protein dimers triggering encapsidation of the complex into the nucleocapsid. Three inhibitors that act at this stage in the life cycle of the virus are Bay 41-4109 [653], GLS4 [654] and NVR-1221 [655].

Following encapsidation of the polymerase and pgRNA complex, the subsequent steps in virus nucleic acid replication take place within the nucleocapsid and involve RNAse H. A potential drug targeting RNase H is b-thujaplicinol, which inhibited the enzyme from genotypes D and H in biochemical assays with IC50 values of 5.9 ± 0.7 and 2.3 ± 1.7 lM, respectively. It also blocked replication of HBV genotypes A and D in culture by inhibiting RNase H activity with an estimated EC50 of 5 lM and a CC50 of 10.1 ± 1.7 lM. Thus, if chemical derivatives of b-thujaplicinol with improved efficacy and reduced toxicity can be identified, such compounds could be used in future regimens of combined therapy with nucleos(t)ide analogues [656].

Maturing nucleocapsids in the final stages of morphogenesis bud through the endoplasmic reticulum membrane. Peptidomimetic compounds that would prevent HBsAg-nucleocapsid interaction and glucosidase inhibitors preventing glycosylation of HBsAg are potential drugs at this stage of the viral life cycle [657].

#### Immunotherapeutic approaches: restoration of adaptive immunity

During CHB infection, HBV-specific T cells are deleted or functionally exhausted, most likely due to the repeated exposure of these cells to large quantities of HBsAg and HBeAg. Exhausted virus specific T cells express inhibitory molecules, such as PD-1 (programmed cell death protein 1), CTLA-4 (cytotoxic T-lymphocyte-associated protein 4), SLAM (signalling lymphocyte activation molecule), and TIM-3 (T-cell immunoglobulin domain and mucin domain 3), and acquire a progressive and step-wise loss of their effector functions [658]. Blocking inhibitory receptors has been shown to partially recover the exhausted T cells of CHB patients in vitro [659], but the in vivo efficacy of this approach is still uncharacterized. Therapeutic vaccination aimed at eliciting the patient’s immune system represents another attractive therapy for HBV. Potential approaches include HBV therapeutic vaccines targeting different HBV proteins [660, 661], vaccine based on immunogenic complexes composed of HBsAg and antihuman HBsAg antibodies [662], or TLR-mediated or anti CD40-mediated stimulation of intrahepatic monocytes or dendritic cells [663, 664]. Improving HBV-specific T cell immunity by engineering HBV-specific T cells through the transfer of HBV-specific T cell receptors (TCR) or HBV-specific chimeric antigen receptors (CARs) represents another novel approach [665, 666].

#### Immunotherapeutic approaches: direct stimulation of innate intrahepatic immunity

Therapeutic strategies aimed at increasing innate immunity exploit the antiviral efficacy of distinct cytokines (tumour necrosis factor-a, IFN-α, IFN-γ and interleukin-1β), mimic the activation of innate immunity during the early phase of acute HBV infection and induce a correct maturation of the adaptive immunity [667]. Strategies include boosting intrahepatic IFN- α levels by TCR-like antibodies conjugated with IFN- α that specifically target HBV-infected hepatocytes [668], use of TLR7 agonists to induce IFN- α production in pDCs (plasmacytoid dendritic cells) [669], stimulating NK and NKT cells by IL-12 and IL-18 [670, 671], and use of TLR8 agonists [672].

However, these new therapeutic approaches have mainly been tested in animal models and await lrarge-scale human studies. A cure for chronic HBV infection requires agents that can target different stages in the life cycle of the virus. However, this requirement must deal effectively with the cccDNA pool by either inhibiting the cccDNA complex formation or destroying infected hepatocytes. The latter is only achievable through immune-mediated mechanisms, a fact that strongly suggests a combination therapy approach for the future [657].

### 4.2 Unresolved issues and unmet needs

The challenges in the management of hepatitis B are still very daunting, and despite significant advances, cure from HBV infection is a far cry. We need to improve our understanding of the natural history of chronic HBV infection, including the role of serum HBsAg levels in the evaluation of the natural history.

The role of noninvasive methods for the evaluation of the severity of liver disease and for the follow-up of treated and untreated patients needs to be established.

The future of hepatitis B treatment will involve personalized decisions regarding when to initiate treatment based on prognostic models/risk calculators that include host genetic and viral markers that predict cirrhosis and HCC.

There is need to assess the impact of long-term treatment in chronic Hepatitis B with normal ALT.

Identify markers that predict successful NA discontinuation.

Assess the safety and efficacy of the combination of Peg-IFN with a potent NA (entecavir or tenofovir) to increase anti-HBe and anti-HBs seroconversion rates.

The future of hepatitis B treatment will also involve personalized decisions regarding choice of treatment based on pharmacogenetics and predicted responses.

There is the need for novel therapies—antiviral agents with new targets in the HBV replication cycle combined with immunotherapies that can restore the host immune response to HBV.

The persistence of cccDNA in HBV-infected cells remains one of the main obstacles to complete eradication of the virus during chronic infection. In that respect, a better understanding of the biochemical steps of cccDNA biosynthesis and epigenetic control of cccDNA is needed. The characterization of the complex interaction between viral and host cellular proteins and/or genomes represent other research challenges that may pave way to identification of new treatment targets.

Further confirmatory studies need to be done on the use of potent NAs from the time of listing to provide a completely HBIG-free oral prophylaxis regimen and thus further improve the outcomes, tolerability and cost effectiveness of liver transplantation for CHB.
